# Red-Light-Induced Cysteine Modifications Suitable for Protein Labeling

**DOI:** 10.1021/acsorginorgau.5c00025

**Published:** 2025-04-10

**Authors:** Tomasz Wdowik, Egor Fedorov, Tina-Thien Ho, Patrick Duriez, Eugen Stulz, Dorota Gryko

**Affiliations:** † 154690 Institute of Organic Chemistry, Polish Academy of Sciences, Kasprzaka 44/52, Warsaw 01-224, Poland; ‡ School of Chemistry and Chemical Engineering & Institute for Life Sciences, 7423 University of Southampton, Highfield, Southampton SO17 1BJ, United Kingdom; § Centre for Cancer Immunology, University Hospital Southampton, Coxford Road, Southampton SO16 6YD, United Kingdom

**Keywords:** photocatalysis, red light, cysteine, proteins, porphyrin, thiol–ene reaction, bioconjugation

## Abstract

The naturally low abundance of cysteine in proteins, combined with its propensity to undergo thiol–ene reactions, makes it a preferred amino acid for various bioconjugations. However, most of these methods rely on the use of UV radiation, radical initiators, or heavy-metal-based photocatalysts, which limits their applicability in complex biological environments. Herein, we report a photocatalyzed thiol–ene radical reaction that overcomes these limitations by employing a porphyrin-based photocatalyst and low-energy red light. This method operates under mild reaction conditions and can be expanded to a cysteinyl desulfurization reaction. As this approach proceeds in aqueous media and facilitates selective transformations of both simple free cysteine and cysteine residues within complex protein, it significantly expands the existing toolbox for cysteine bioconjugation.

## Graphical abstract

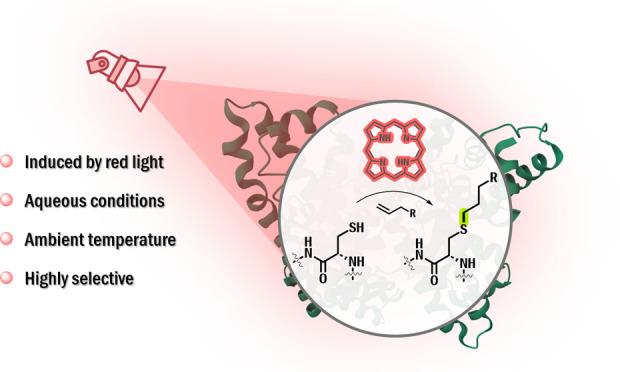

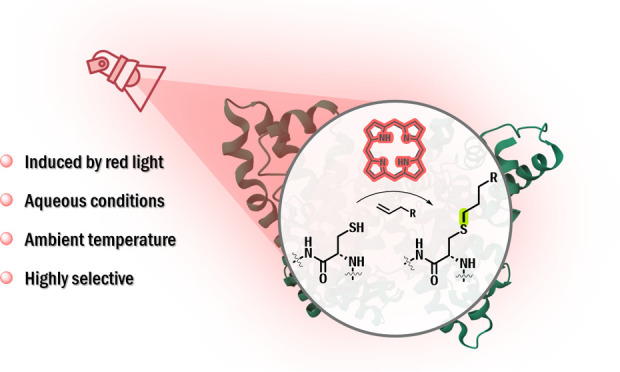

## Introduction

Multiple fields intersecting chemistry and biology, such as diagnostics, biocatalysis, and materials science, have benefited from the opportunities offered by bioconjugation. The formation of a stable covalent bond between a biomolecule and another compound, the essence of this technique, has been the central subject of numerous studies involving proteins, enzymes, nucleic acids, carbohydrates, and other biomolecules. Certain amino acid residues, such as lysine, cysteine, and tyrosine, have been used particularly frequently in this context due to their low abundance and/or their chemical properties.^1,2^ For cysteine, the thiol–ene reaction has been among the most commonly explored pathways of functionalization.^3^

The thiol–ene reaction represents a versatile synthetic tool widely employed in various fields such as polymerization, synthetic vaccine production, or peptide modification.^4–6^ Its efficiency aligns with the criteria of a click reaction, making it increasingly relevant in bioconjugate chemistry.^7,8^ Historically, photochemical radical thiol–ene processes have relied on UV radiation or radical initiators, which are not always biocompatible. As an alternative induction method, the application of visible light in thiol–ene transformations has recently become intensely explored.^9^ In this context, Yoon and co-workers pioneered blue-light photocatalyzed thiol–ene reactions to modify glutathione utilizing a Ru photocatalyst and *p*-toluidine as a redox mediator (Scheme 1A).^10^ More intricate peptide and protein conjugates were achieved using a water-soluble quinolinone derivative (*Q*_PEG_) serving as both the photocatalyst and the alkene coupling partner.^11^ While various protocols have been reported for visible-light-induced thiol–ene reactions, most are based on high-energy blue light.^9^ Although it is an improvement compared to the UV-based approach, blue light can still cause damage to sensitive proteins.^12^ The use of red light irradiation for biological systems applications is highly desired due to its low energy, minimizing side reactivity and compound degradation, and enabling deeper penetration in tissue,^13^ potentially facilitating *in vivo* reactions. Despite the growing interest in red and near-infrared radiation in photocatalysis, its extension to bioconjugation remains underexplored.^14,15^ A rare example of this approach was demonstrated by Schlau-Cohen and co-workers in the thiol–ene reaction of glutathione, which was catalyzed by a biohybrid photocatalyst composed of R-phycoerythrin, and a Ru complex that was effective in both green- and red-light-induced reactions.^16^ In this reaction, R-phycoerythrin is responsible for harvesting light that is subsequently transferred to the Ru-based photocatalyst. Very recently, two reports on the application of red light in protein labeling have been disclosed. One research explores fluoroalkylation using porphyrin or helical carbenium ion-based photocatalysts,^17^ while the other relies on the generation of carbenes from aryl­(trifluoromethyl)­diazo compounds upon red light irradiation in the presence of osmium-based photocatalyst.^18^

**Scheme 1:**
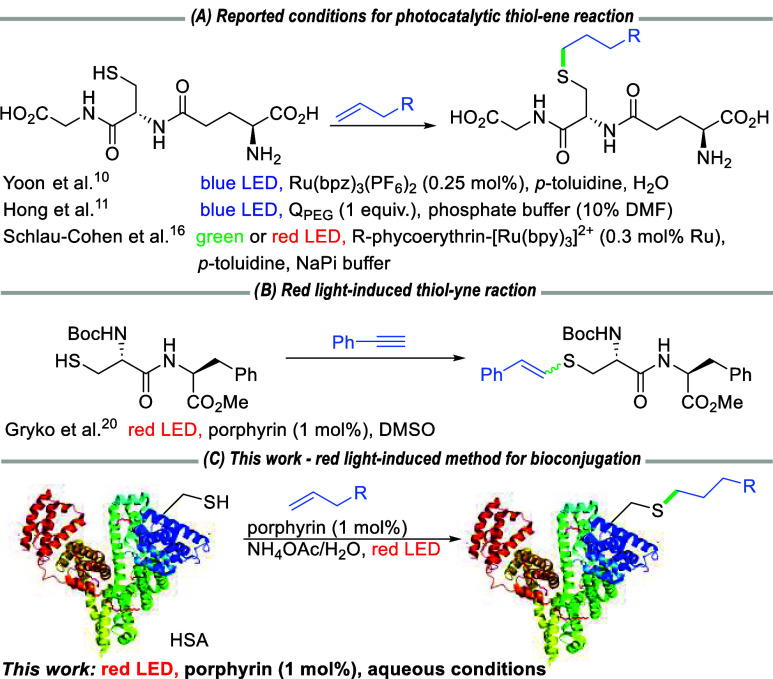
Visible-Light-Induced Thiol–Ene/Yne Reactions^21^

Our interest in porphyrinoids has shown that these compounds can act efficiently as both photoredox catalysts and photosensitizers.^19^ They are compatible with red light irradiation, facilitating biomolecule modifications in organic solvents through several means, including the thiol–yne reaction (Scheme 1B).^20^ Here, we present the red-light-induced thiol–ene reaction in aqueous media suitable for the functionalization of biologically relevant complex molecules (Scheme 1C).

## Results and Discussion

We initiated our investigation by examining the reaction of glutathione (GSH) and 2-methylbut-3-en-2-ol (**1**) as model substrates to ensure the complete solubility of the starting materials (Table 1). Tetraphenylporphyrin (H_2_TPP), employed in our previous studies, yielded product **2**, although in a poor yield (entry 2), likely due to the low solubility of H_2_TPP in water. Transitioning to a more polar tetrakiscarboxy-substituted phenyl porphyrin (TCPP) led to an improved yield (entry 4), further enhanced by the addition of ammonium acetate (0.15 M, 3 equiv), which improves the solubility of the photocatalyst (entry 1). However, the solubility of the porphyrin is not the only predictor of a successful outcome, as evidenced by the reaction in the presence of cationic TMPyP, (entry 3), which yielded only 7% of the desired sulfide. Moreover, after the addition of acidic substrates, some TCPP precipitation was observed. This indicates that charge also plays an important role, though the exact reason for it still needs to be established. Usually, product formation coincided with the generation of a small amount of an oxidized analogue (namely, sulfonic acid) and disulfide (GSSG). Sulfonic acid formation (possibly involving singlet oxygen generation facilitated by the porphyrin) can be mitigated by degassing the reaction mixture, while disulfide formation can be suppressed by reducing glutathione concentration to 50 mM. A further reduction in concentration (to match the intracellular/mitochondrial GSH content of 10 mM) resulted in a decrease in reactivity, which could be compensated by increasing photocatalyst loading (entries 5 and 6). With the optimized conditions established, we proceeded to explore the scope of this reaction (Scheme 2). Remarkably, we achieved high reactivity even with an alkene partner amount reduced to 1.1 equiv. The reaction demonstrated good performance with unprotected cysteine and homocysteine, as well as their derivatives, including captopril (angiotensin-converting enzyme inhibitor). A major challenge often raised in the development of bioconjugation reactions is the chemoselectivity of these processes, which aim to promote the reaction of a specific nucleophilic site over others present in the complex molecule.^22^ Notably, in our approach, cysteine residues within the peptide (glutathione) exhibited selective reactivity in the presence of various unprotected functional groups such as amine, alcohol, carboxylic acid, or (thio)­ether. Additionally, even with moderately water-soluble alkene, such as a biotin derivative, we obtained product **11** with respectable yield, likely due to the high solubility of the product. We were also pleased to see coenzyme A (CoA) reacting under our conditions. To the best of our knowledge, this represents the first reported example of the bioconjugation of CoA via the radial thiol–ene reaction.

**Table 1:**
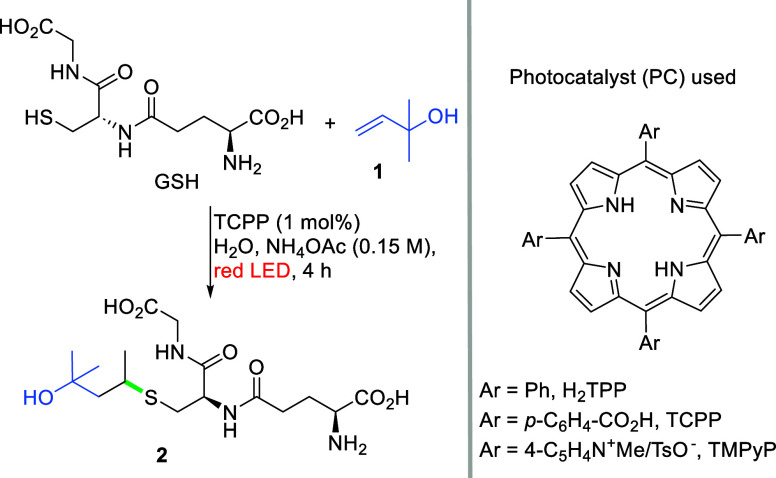
Optimization of the Red-Light-Induced Radical Thiol–Ene Reaction^a^

entry	variation from standard conditions	yield of **2** [%]^b^
1	none	87
2	H_2_TPP instead of TCPP	23
3	TMPyP instead of TCPP	7
4	no NH_4_OAc	65
5	K_2_CO_3_ (0.025 M) instead of NH_4_OAc	70
6	[GSH] = 10 mM	46
7	[GSH] = 10 mM, 5 mol % of PC	60

a Reaction conditions: GSH (c 50 mM), **1** (3 equiv), 660 nm irradiation.

b Yields determined by reversed-phase high-performance liquid chromatography (RP-HPLC).

**Scheme 2:**
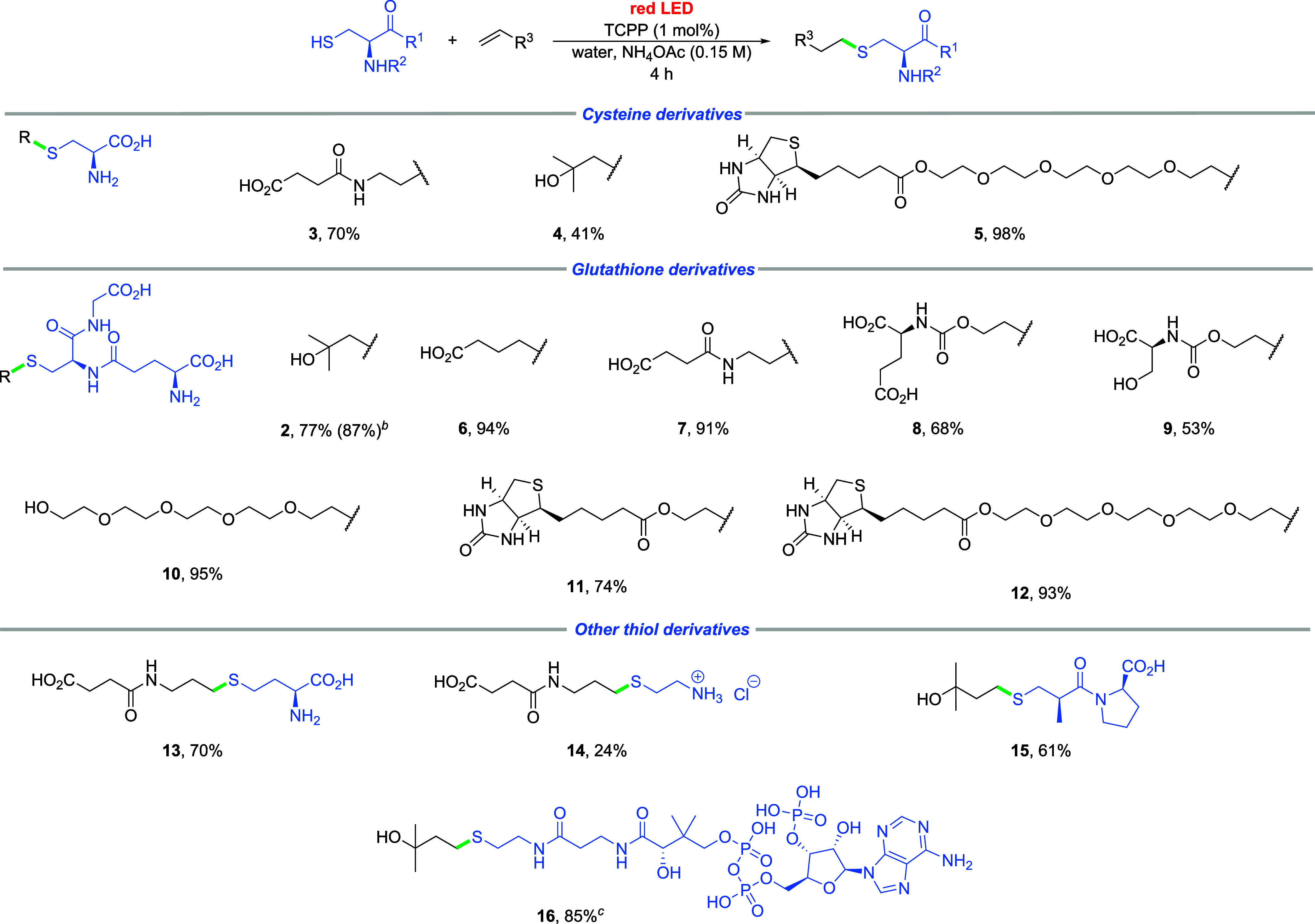
Scope of the Red-Light-Induced Radical Thiol–Ene Reaction^c^

b Three equiv of alkene used.
c 3.5 equiv of alkene used.
a Optimized reaction conditions: thiol (0.2 mmol, 1 equiv), alkene (0.22–0.24 mmol, 1.1–1.2 equiv), TCPP (0.002 mmol, 1 mol %) NH_4_OAc solution in water (0.15 M, 4 mL), red LED­(660 nm), 4 h. Yields determined by quantitative ^1^H NMR analysis with an internal standard.

To further verify the selectivity of our approach and its suitability in complex mixtures, we conducted our model reaction with the addition of a commercially available dietary supplement containing 10 essential amino acids (EAA, Table 2). Remarkably, even in the presence of these additional spectator components ranging from 0.04 to 1.79 equiv of each amino acid (a total of 2.5 mg of EAA/mg of GSH), we observed the formation of the thiol–ene reaction product in almost quantitative yield (see the Supporting Information for further details); thus, any free NH_2_, CO_2_H, OH, SMe, and guanidino groups do not affect the thiol–ene reaction described here.

**Table 2:** EAA for the Model Reaction

entry	amino acids	composition [mg/1 g]	amount added [equiv]
1	Leu	300	1.79
2	Val	150	1.00
3	Ile	150	0.89
4	Lys HCl	135	0.58
5	Thr	70	0.46
6	Phe	70	0.33
7	Met	45	0.24
8	Arg	40	0.18
9	His	30	0.15
10	Trp	10	0.04

Furthermore, the cysteine residue in a complex biomolecule, such as a protein, often forms a disulfide bond to stabilize the secondary structure, making it inaccessible for thiol–ene reactions.^23^ We were intrigued by the possibility of combining our protocol with disulfide reduction to render disulfides compatible with our method (Scheme 3). Using standard disulfide bond reducing agents such as dithiothreitol (DTT) or tris­(2-carboxyethyl)­phosphine (TCEP) to the disulfide of GSH as a model reaction, we successfully obtained the product, albeit in a lower yield compared to the direct reaction of free thiol (up to 64 vs 87%, Scheme 3, Table 3). Furthermore, we confirmed the unreactivity of the disulfide itself (Table 3, entry 1).

**Scheme 3:**
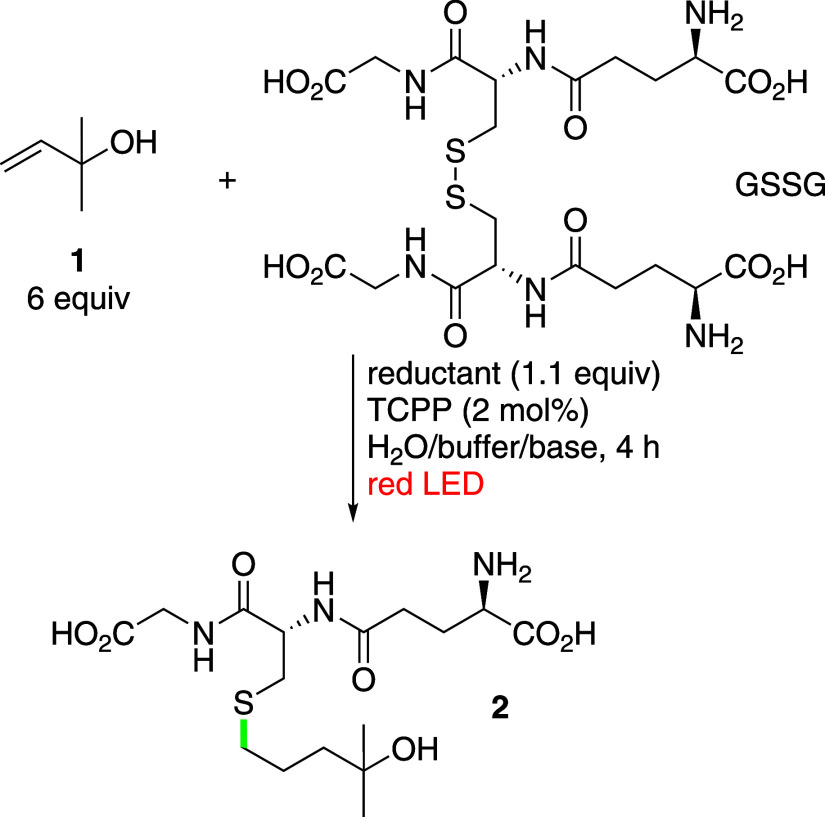
Red Light-Induced Radical Thiol–Ene Reaction of Disulfide

**Table 3:** Red-Light-Induced Radical Thiol–Ene Reaction of Disulfide^a^

entry	reductant	buffer/base	yield of **2** [%]^a^
1	none	NH_4_OAc (0.15 M)	0
2	TCEP-HCl	NH_4_OAc (1 M)	55
3	TCEP-HCl	KOAc (3 M)	64
4	TCEP-HCl	NaOH (pH = 8–9)	12
5	DTT	NH_4_OAc (1 M)	55

a Yields determined by RP-HPLC.

Encouraged by these results, we aimed to expand our methodology for protein bioconjugation. Human serum albumin (HSA) was selected as the model protein due to its excellent solubility in water and the presence of one free cysteine (in the reduced form) at position 34 available for conjugation, in addition to 17 disulfide bridges that are deemed unavailable. To facilitate this conjugation, HSA was reacted with an alkene that was decorated with a glycol linker to enhance the solubility in water and a biotin moiety to enable avidin–biotin complexation for analysis.

The Cys-34 residue on a commercially available HSA is mostly blocked with a cysteine; therefore, samples were initially treated with DTT to form an accessible thiol group following the protocol published by Seki et al.^24^ Initial attempts to achieve protein modification via the radical thiol–ene reaction under the conditions recommended for glutathione conjugation were unsuccessful. However, by increasing the equivalents of the allylated biotin moiety to 10 equiv and screening of different buffer conditions to find the best initial system, we successfully obtained the biotinylated protein; the results were confirmed by Dot blot analysis (Scheme 4). For this analysis, the samples were loaded onto a nitrocellulose membrane and labeled with ExtrAvidin-Peroxidase, which consists of avidin conjugated with a peroxidase that oxidizes Luminol to form 3-aminophthalate. The chemiluminescence signal is then detected in a ChemiDoc Imaging system. Notably, augmenting the allylated biotin equivalents for a further 5-fold markedly increased the signal, indicating a higher conjugation efficiency. Additionally, we determined that red light irradiation is crucial for the effective thiol–ene reaction and that the presence of the photocatalyst greatly enhances the degree of biotinylation of the protein. To corroborate these observations, size exclusion chromatography was performed, and for the biotinylated sample, two distinct peaks were present in the chromatogram, which are absent in the native protein (Figure 1). The second peak represents the excess of allylated biotin in the reaction. We attribute the first peak to the biotinylated HSA, which elutes earlier due to its larger size. Its analysis by MALDI-TOF MS confirms that it has a higher molecular weight (*m*/*z* 67759 Da) compared to free HSA (*m*/*z* 66488 Da). The mass difference suggests that more than one biotin could couple to HSA (between two and three, see the Supporting Information for further details); however, the resolution obtained at this stage does not allow for more detailed analysis.

**Figure 1:**
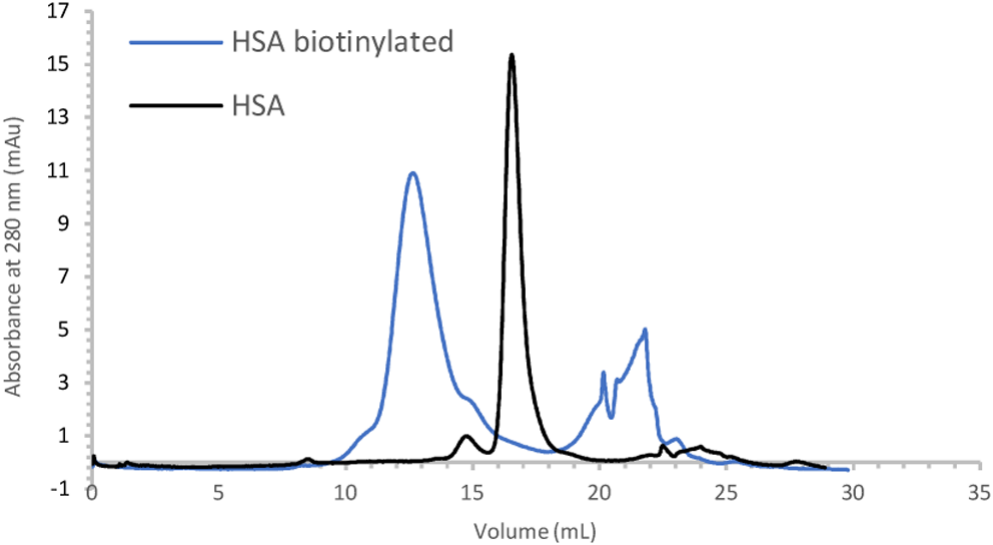
Chromatogram showing the biotinylation of HSA.

**Scheme 4:**
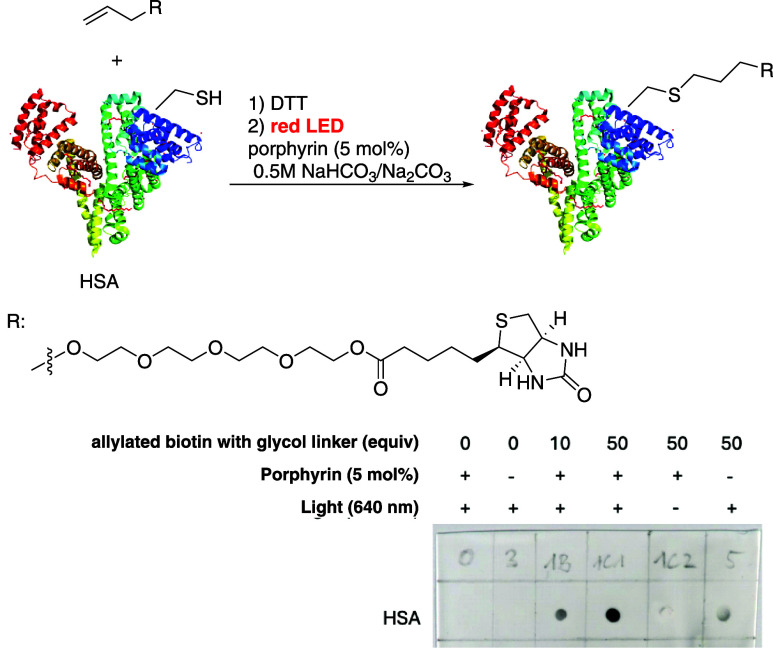
Red-Light-Induced Radical Thiol–Ene Reaction for Bioconjugation of the Protein

The analytical data using HPLC, MS, and Dot blot analysis confirm that the addition works best in the presence of porphyrin and light, and in their absence, no reaction occurs. In the presence of light only, a faint product spot can be seen, indicating that light-induced coupling is possible to some extent; this could also mean addition to other amino acids, for example, tyrosine through a radical reaction, which could lead to the higher *m*/*z* value observed. Based on our reactions described above, we assume that cysteine is labeled predominantly, but a more detailed mapping was out of scope for this investigation. This would help to determine which other amino acid residues can be labeled, for example, other cysteins that are embedded in disulfide bridges (see Scheme 3). However, as a preliminary experiment, these results clearly demonstrate that the functionalization of proteins is feasible using our system, and coupling efficiency is greatly enhanced. The system requires optimization in terms of reaction conditions, site-specificity analysis, and scope of the labeling, which is now part of ongoing work.

Furthermore, inspired by precedent studies on the application of visible light in a cysteinyl desulfurization reaction,^16,25^ we decided to evaluate our porphyrin-based system for this transformation (Scheme 5). To our delight, in the absence of olefin, desulfurized glutathione formed in a useful yield without the need for additional optimization.

**Scheme 5:**
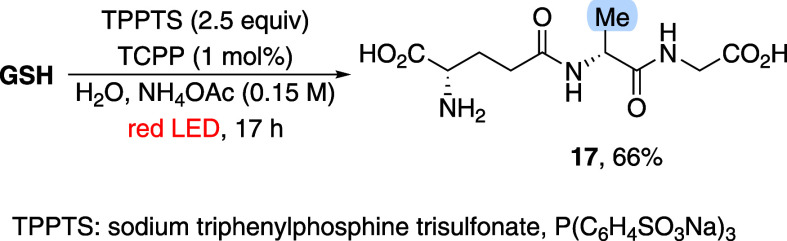
Red-Light-Induced Cysteinyl Desulfurization

## Conclusions

In conclusion, we have developed a radical thiol–ene and a cysteinyl desulfurization reaction utilizing low-energy red light in aqueous media. This process employs porphyrin as a photocatalyst and can effectively modify cysteine residues ranging from free amino acids to peptides and proteins. Key features of this method include compatibility with aqueous environments, selective reactivity in the presence of other amino acid residues, and the use of a biocompatible photocatalyst (free of heavy metals). Furthermore, the ability of red light to penetrate various media, including tissues, makes it highly suitable for further applications in complex biological environments.

## Experimental Section

### General Procedure for the Red-Light-Induced Radical Thiol–Ene Reaction

TCPP (1.58 mg, 0.002 mmol, 1 mol %) was placed in the 10 mL glass vial containing a stirring bar and dissolved in the 0.15 M solution of NH_4_OAc (4 mL) and degassed (Ar flow, sonication) for ca. 15 min. To this solution, thiol (0.2 mmol, 1 equiv) and alkene (0.22–0.24 mmol, 1.1–1.2 equiv) were added, and the mixture was flushed with Ar. The sealed vial was then irradiated (660 nm, 100% power of the UOSlab Miniphoto photoreactor) for 4 h. After that time, the mixture was analyzed by RP-HPLC and quantitative ^1^H NMR (the spectra were recorded at 298 K on Varian 600 MHz NMR instrument; acquisition parameters: suppression of the water signal PRESAT, number of scans: 16, acquisition time: 2 s, presaturation delay: 5 s, relaxation delay: 8 s, dummy scans before start of experiment: 4). For characterization, the products were isolated (as TFA salts) by preparative RP-HPLC followed by lyophilization.

## Supplementary Material



## Data Availability

The data underlying this study are available in the published article and the Supporting Information.

## Notes

Optimization details, experimental procedures, and characterization data for all new compounds (PDF)
