# Supplementary material for: Red-Light-Induced Cysteine Modifications Suitable for Protein Labeling
Source: ACS Org Inorg Au. 2025 Apr 10;5(4):238–43. doi: 10.1021/acsorginorgau.5c00025 (PMC12332779; doi:10.1021/acsorginorgau.5c00025)

SUPPORTING INFORMATION

RED-LIGHT-INDUCED CYSTEINE MODIFICATIONS SUITABLE FOR PROTEIN LABELING

Tomasz Wdowik,[a] Egor Fedorov,[a] Tina-Thien Ho,[b,c] Patrick Duriez,[c]

Eugen Stulz,[b*] and Dorota Gryko[a*]

[a] *Institute of Organic Chemistry, Polish Academy of Sciences, Kasprzaka 44/52, 01-224 Warsaw, Poland.*
[b] *School of Chemistry and Chemical Engineering & Institute for Life Sciences, University of Southampton, Highfield, Southampton SO17 1BJ, United Kingdom.*
[c] *Centre for Cancer Immunology, University Hospital Southampton, Coxford Road, Southampton SO16 6YD, United Kingdom.*

e-mail: dorota.gryko@icho.edu.pl
est@soton.ac.uk

TABLE OF CONTENTS

1. GENERAL INFORMATION 3

2. PHOTOREACTOR SETUPS 5

3. RED-LIGHT-INDUCED RADICAL THIOL-ENE REACTION 6

3.1 Reaction development: general procedure 6

3.2 Optimization studies 6

3.2 Scope of the red-light-induced radical thiol-ene reaction 9

3.3 Amino acids tolerance screening with EAA 19

3.4 Optimization of the red light-induced radical thiol-ene reaction of disulfide with olefin ... 20

3.5 Bioconjugation reaction with HSA 20

4. RED-LIGHT-INDUCED CYSTEINYL-DESULFURIZATION 23

5. NMR SPECTRA 24

Trifluoroacetate salt of (*R*)-4-((3-((2-amino-2-carboxyethyl)thio)propyl)amino)-4-oxobutanoic acid (3·TFA) 24

Trifluoroacetate salt of *S*-(3-hydroxy-3-methylbutyl)-L-cysteine (4·TFA) 26

Trifluoroacetate salt of (*R*)-2-amino-21-oxo-25-((3a*S*,4*S*,6a*R*)-2-oxohexahydro-1*H*-thieno[3,4-d]imidazol-4-yl)-8,11,14,17,20-pentaoxa-4-thiapentacosanoic acid (5·TFA) 27

Trifluoroacetate salt of *N*5-((*R*)-1-((carboxymethyl)amino)-3-((3-hydroxy-3-methylbutyl)thio)-1-oxopropan-2-yl)-L-glutamine (2·TFA) 29

Trifluoroacetate salt of N5-((R)-3-((4-carboxybutyl)thio)-1-((carboxymethyl)amino)-1-oxopropan-2-yl)-L-glutamine (6·TFA) 31

Trifluoroacetate salt of *N*5-((*R*)-1-((carboxymethyl)amino)-3-((3-(3-carboxypropanamido)propyl)thio)-1-oxopropan-2-yl)-L-glutamine (7·TFA) 32

Trifluoroacetate salt of (3*S*,12*R*,17*S*)-17-amino-12-((carboxymethyl)carbamoyl)-5,14-dioxo-6-oxa-10-thia-4,13-diazaheptadecane-1,3,17-tricarboxylic acid (8·TFA) 34

Trifluoroacetate salt of (2*S*,11*R*,16*S*)-16-amino-11-((carboxymethyl)carbamoyl)-2-(hydroxymethyl)-4,13-dioxo-5-oxa-9-thia-3,12-diazaheptadecanedioic acid (9·TFA) 35

Trifluoroacetate salt of (18*R*,23*S*)-23-amino-18-((carboxymethyl)carbamoyl)-1-hydroxy-20-oxo-3,6,9,12-tetraoxa-16-thia-19-azatetracosan-24-oic acid (10·TFA) 36

Trifluoroacetate salt of *N*5-((*R*)-1-((carboxymethyl)amino)-1-oxo-3-((3-((5-((3a*S*,4*S*,6a*R*)-2-oxohexahydro-1*H*-thieno[3,4-d]imidazol-4-yl)pentanoyl)oxy)propyl)thio)propan-2-yl)-L-glutamine (11·TFA) 37

Trifluoroacetate salt of (24*R*,29*S*)-29-amino-24-((carboxymethyl)carbamoyl)-5,26-dioxo-1-((3a*S*,4*S*,6a*R*)-2-oxohexahydro-1*H*-thieno[3,4-d]imidazol-4-yl)-6,9,12,15,18-pentaoxa-22-thia-25-azatriacontan-30-oic acid (12·TFA) 38

Trifluoroacetate salt of S-(3-(3-carboxypropanamido)propyl)-L-homocysteine (13·TFA) 40

Trifluoroacetate salt of 4-((3-((2-aminoethyl)thio)propyl)amino)-4-oxobutanoic acid (14·TFA) ... 42

((*R*)-3-((3-hydroxy-3-methylbutyl)thio)-2-methylpropanoyl)-D-proline (15·TFA) 44

Trifluoroacetate salt of CoA conjugate (16·TFA) 45

1. GENERAL INFORMATION

Materials

All solvents and commercially available reagents were purchased from Sigma-Aldrich, TCI, Ambeed, or Acros Organics as reagent grade and were used without further purification, unless otherwise stated. Porphyrins were purchased from PorphyChem. Essential amino acid (EEA) supplement was purchased from Ostrovit. All deuterated solvents used were purchased from Eurisotop.

General Procedures

Unless otherwise stated, photochemical reactions were performed in 10 ml glassy vials sealed with aluminum caps containing a rubber septum. Reactions were monitored by RP-HPLC equipped with UV detector, eluent: acetonitrile/water/formic acid or trifluoracetic acid.

Instrumentation

NMR spectra were recorded at ambient temperature on Bruker 400 or 500 MHz and Varian 500 or 600 MHz. Chemical shifts in deuterated dimethyl sulfoxide are reported in ppm relative to the solvent peak (2.50 ppm for ^{1}H and 39.52 ppm for ^{13}C). Chemical shifts in deuterated water were measured using TMSP-d_4 as the internal standard for both ^{1}H and ^{13}C NMR spectra, with chemical shifts referenced to its signals at 0.00 ppm. Multiplicities are given as: singlet (s), doublet (d), triplet (t), quartet (q), pentet (p), multiplet (m). All spectra were processed with the MestReNova 12.0.4-22023 software package, and coupling constants are reported as observed.

LR and HRMS Low-resolution mass spectra (LRMS) were recorded on an Applied Biosystems API 365 mass spectrometer using electrospray ionization (ESI) technique. High-resolution mass spectra (HRMS) were recorded on Waters SYNAPT G2-S HDMS instrument using electrospray ionization (ESI) or atmospheric pressure chemical ionization (APCI) with time-of-flight detector (TOF).

HPLC analyzes for reaction monitoring were performed on a KNAUER HPLC system equipped with Kromasil Eternity 5-C18, 250 x 4.6 mm column with precolumn, using UV-Vis detection (wavelength: 214 nm) at room temperature. Flow rate: 1 ml/min, elution: acetonitrile/water/formic acid or trifluoracetic acid. For the product isolation, HPLCs were performed on a KNAUER system equipped with Eurospher II 100-10 C18, 250 x 20 mm column with precolumn, using UV-Vis detection (wavelength: 214 nm) at room temperature, elution: acetonitrile/water/trifluoracetic acid, flow rate: 19 ml/min).

IR analyzes were performed using a JASCO FT/IR-6000 spectrometer.

Dot-blot imaging was performed on an Amersham ImageQuant™ 800.

Size exclusion chromatography was conducted on a Bio-Rad NGC Chromatography System using a Superdex™ 200 Increase 10/300 GL column with UV-VIS detection (wavelength: 280 nm). ChromLab (Version 6.0.0.35) was used to monitor and analyze the purification. Fractions were collected with a BioFrac™ Fraction Collector.

Protein MS were performed using a MaXis (Bruker Daltonics, Bremen, Germany) time-of-flight (TOF) and MALDI–TOF/TOF UltrafleXtreme MS (Bruker Daltonics, Bremen, Germany) mass spectrometers. Samples were introduced to the mass spectrometer via a Dionex Ultimate 3000 autosampler and uHPLC pump. Ultrahigh performance liquid chromatography was performed using a Waters, Acquity UPLC BEH C18 (50 mm x 2.1 mm 1.7um) column. Gradient elution from 20% acetonitrile (0.2% formic acid) to 100% acetonitrile (0.2% formic acid) was performed in five minutes at 0.6 ml/min. Positive / negative electrospray ionization mass spectra were recorded.

Nomenclature

Nomenclature follows the suggestions proposed by the ChemDraw 22.2.0.3300 software of Revvity Signals.

2. PHOTOREACTOR SETUPS

Light-mediated reactions were carried out in the UOSlab Miniphoto photoreactor. Red light (maximum at 660 nm) was supplied to each reaction vial with the use of 7 LUMINUS LED units (of overall 25 W intensity when 100% power applied) positioned a few mm away from the vial bottom. The ambient temperature of the LED block was maintained by cooling with Huber MiniChiller 300.
https://en.uoslab.com/download/Photochemical_reactors.pdf (available on January 8, 2025)

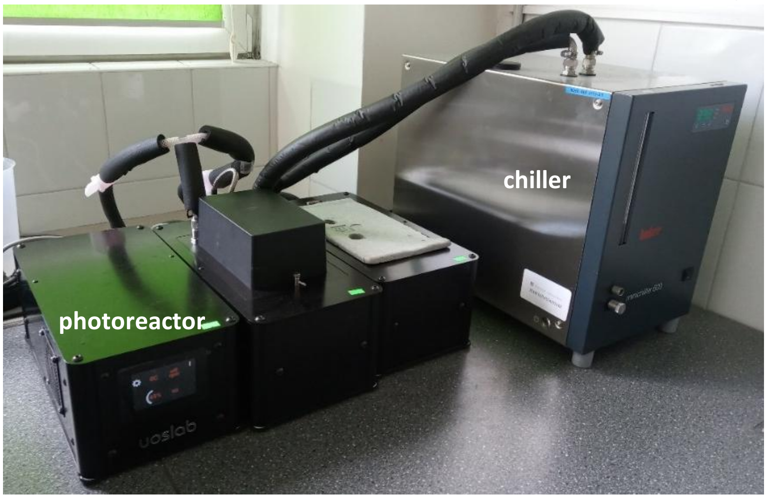

Figure S1. Commercially available UOSlab Miniphoto photoreactor setup.

Bioconjugation experiments were performed using a single Kessil 640 nm LED lamp.

3. RED-LIGHT-INDUCED RADICAL THIOL-ENE REACTION

3.1 Reaction development: general procedure

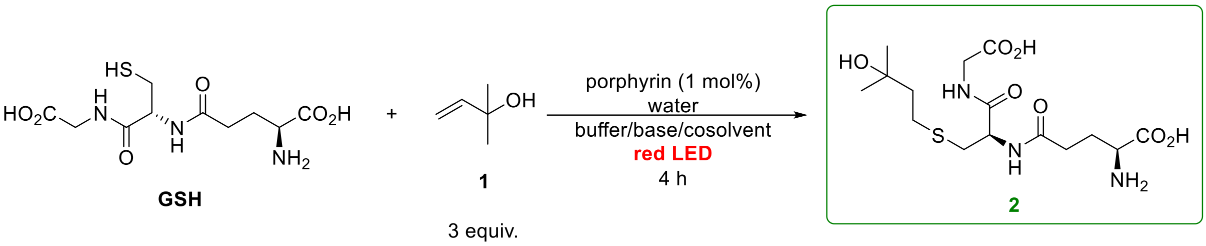

A porphyrin (0.002 mmol, 1 mol%) was placed in a 10 ml glass vial containing a stirring bar and dissolved/suspended in a buffer/base solution or a solvent (4 ml) and degassed (Ar flow, sonication) for ca. 15 min. To this mixture, glutathione (62mg, 0.2 mmol, 1.0 equiv.) and 2-methyl-3-buten-2-ol (52 mg, 0.6 mmol, 3.0 equiv.) were added, and the mixture was flushed with Ar. The sealed vial was then irradiated (660 nm, 100% power of the UOSlab Miniphoto photoreactor) for 4 h. After that time, the mixture was analysed by RP-HPLC and NMR.

3.2Optimization studies

3.1.1 Screening of photocatalysts

Commercially available porphyrins and chlorins were evaluated in the model reaction (performed according to the procedure described in Section 3.1.)

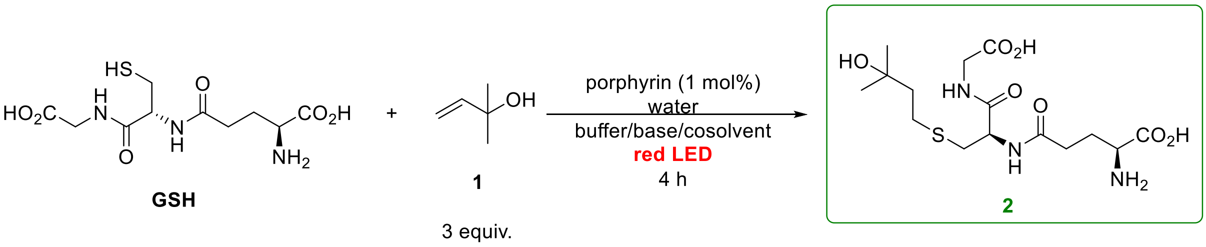

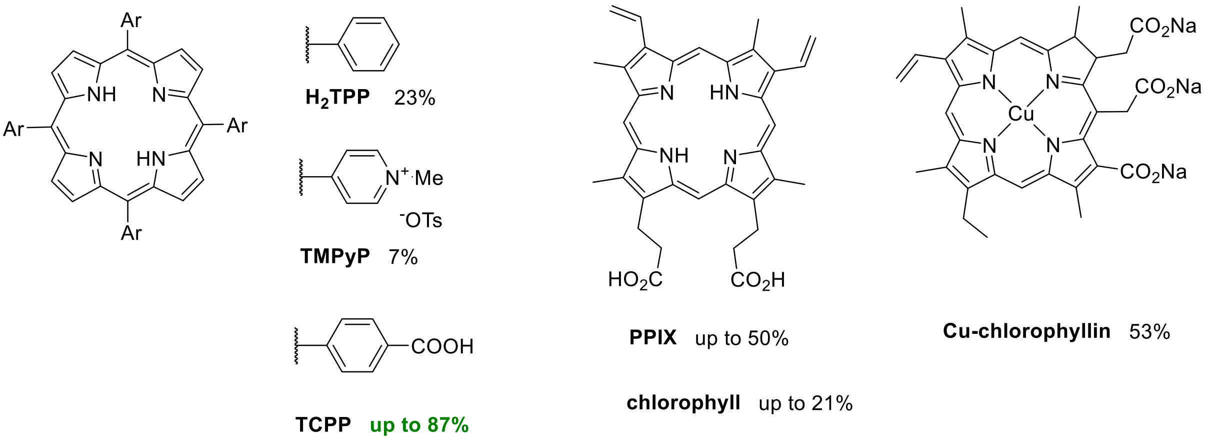

Conclusion: due to the highest reactivity, tetrakis(4-carboxyphenyl)porphyrin (TCPP) was selected for the subsequent experiments.

3.1.2 *Screening of buffer solutions*

Screening of buffer solutions was performed for 2-methyl-3-buten-2-ol and pent-4-enoic acid to ensure hight yield for both neutral and acidic starting materials.

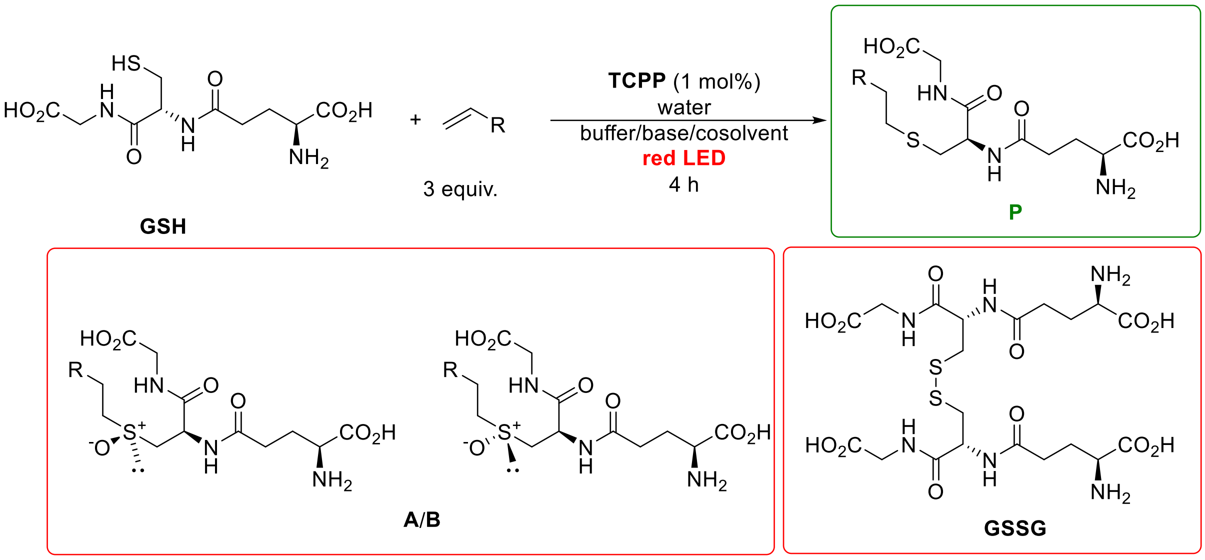

Entry	Alkene	Solvent/buffer	HPLC results				
			**GSH**	**P**	**A/B**	**A/B**	**GSSG**
1	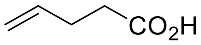	PBS (2 ml)	0%	80%	9%	8%	3%
2	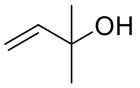	PBS (2 ml)	6%	16%	9%	70%[a]	
3	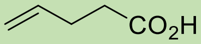	0.15 M NH_4OAc (4 ml)	4%	87%	4%	4%	1%
4	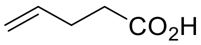	0.15 M K_2CO_3 (4 ml)	0%	66%	17%	14%	2%
5	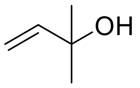	0.15 M NH_4OAc (4 ml)	0%	71%	7%	18%[a]	
6	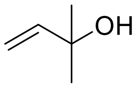	0.1 M K_2CO_3 (2 ml)	0%	48%	15%	37%[a]	
7	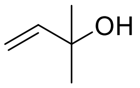	H_2O (5 ml) (*freeze-thaw*)	30%	65%	1%	4%[a]	
8	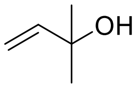	0.1 M K_2CO_3 (2 ml) (*freeze-thaw*)	14%	54%	0%	0%	32%
9	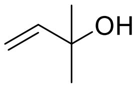	0.2 M NH_4OAc (2 ml)	2%	31%	13%	10%	44%
10	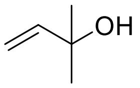	0.025 M K_2CO_3 (4 ml)	9%	70%	9%	8%	5%
11	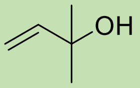	0.15 M NH_4OAc (4 ml) (Ar, sonication)	12%	87%	0%	0%	2%

[a] Mixture of **A/B** and **GSSG**

Conclusion: *due to the good solubility of TCPP in the NH_4OAc solution, as well as its compatibility with proteins, it was chosen as the preferred reaction medium.*

3.1.3 Optimization – miscellaneous parameters

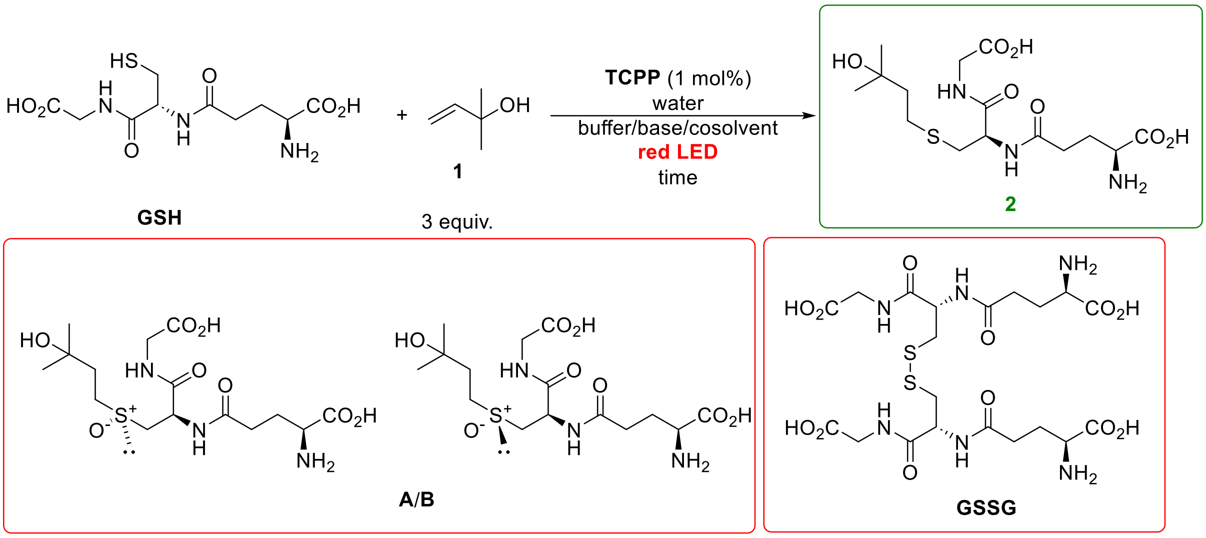

Entry	Solvent	Time	HPLC results					Comment
			**GSH**	**2**	**A/B**	**A/B**	**GSSG**	
1	0.15 M NH_4OAc (4 ml)	3 h	10%	78%	3%	4%	6%	
2	0.15 M NH_4OAc (4 ml)	3 h	13%	79%	2%	2%	4%	reaction under Ar
3	0.15 M NH_4OAc (4 ml)	4 h	15%	79%	1%	1%	4%	using a Kessil lamp
4	H_2O +5% DMSO (4 ml)	3 h	20%	46%	1%	33%[a]		
5	H_2O +5% HFIP (4 ml)	4 h	7%	59%	15%	20%[a]		

[a] Mixture of **A/B** and **GSSG**

Conclusions: *a) degassing reaction mixture and performing the reaction under Ar helps reducing the formation of the oxidized products A, B, and GSSG; b) the reaction can be performed using the Kessil lamp instead of the UOSlab Miniphoto photoreactor with no change in the reactivity; c) addition of organic cosolvent lowers the selectivity of the reaction resulting from the increased formation of oxidized products.*

3.1.4 Low concentration experiments

As part of development of the conditions compatible with the biological environment, reactions were performed at low concertation of GSH, matching its concertation in cells (1-10 mM) and mitochondria (10-14 mM).

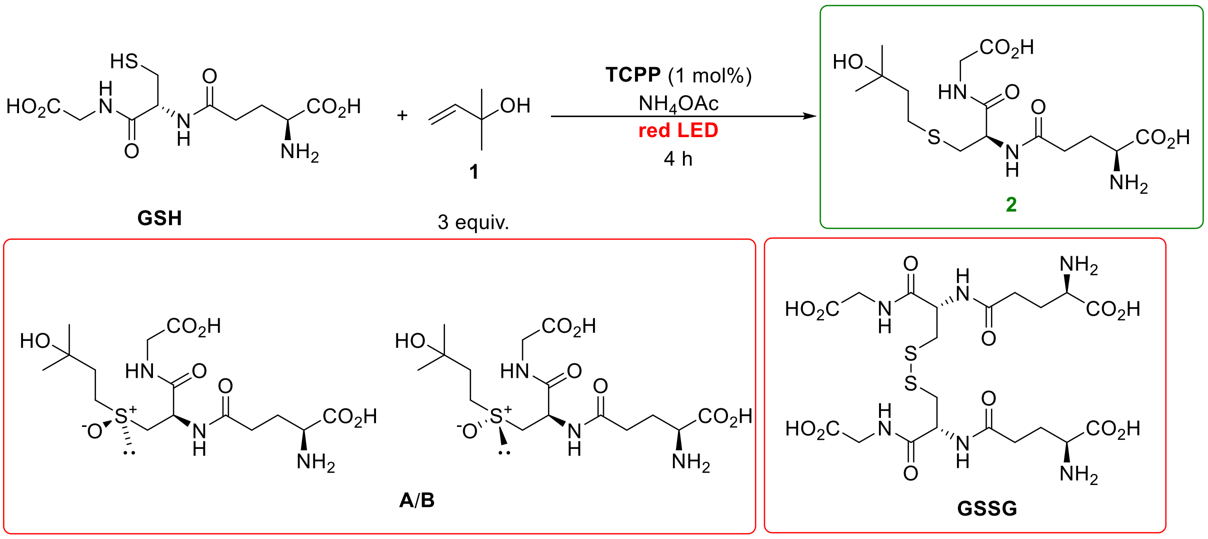

Entry	[GSH]	[porhyrin]	[NH_4OAc]	HPLC results				
				**GSH**	**2**	**A**	**B**	**GSSG**
1	50 mM	1 mol%	0.15 M	12%	87%	0%	0%	2%
2	10 mM	1 mol%	0.03 M	42%	46%	4%	1%	8%
3	10 mM	5 mol%	0.15 M	31%	60%	3%	3%	4%

Conclusion: *reactions with higher dilution proceed slower, with only a modest loss of selectivity. With the same glutathione concentration, this can be improved with a higher loading of the photocatalyst and NH_4OAc concentration.*

3.2 Scope of the red-light-induced radical thiol-ene reaction

3.2.1 General procedure

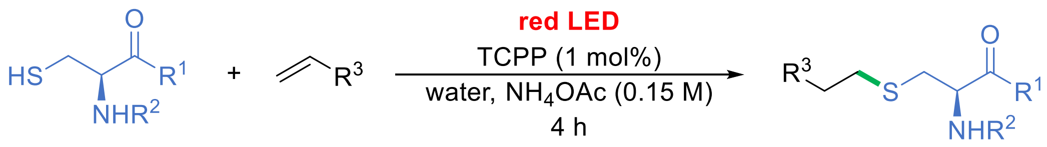

TCPP (1.58 mg, 0.002 mmol, 1 mol%) was placed in the 10 ml glass vial containing a stirring bar and dissolved in the 0.15 M solution of NH_4OAc (4 ml) and degassed (Ar flow, sonication) for ca. 15 min. To this solution, thiol (0.2 mmol, 1 equiv.) and alkene (0.22-0.24 mmol, 1.1-1.2 equiv.) were added, and the mixture was flushed with Ar. The sealed vial was then irradiated (660 nm, 100% power of the UOSlab Miniphoto photoreactor) for 4 h. After that time, the mixture was analyzed by RP-HPLC and quantitative ^{1}H NMR (the spectra were recorded at 298 K on Varian 600 MHz NMR instrument;

acquisition parameters: suppression of the water signal PRESAT, number of scans: 16, acquisition time: 2s, presaturation delay: 5s, relaxation delay: 8s, dummy scans before start of experiment: 4). For characterization, the products were isolated (as TFA salts) by preparative RP-HPLC followed by lyophilization.

3.2.2 Characterization of the products

(*R*)-4-((3-((2-amino-2-carboxyethyl)thio)propyl)amino)-4-oxobutanoic acid (3)

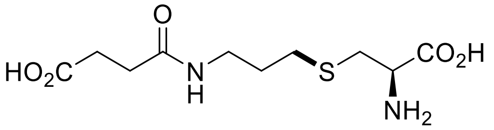

Following the general procedure described in Section 3.2.1, compound **3** was obtained from cysteine (24 mg, 0.20 mmol) and 4-(allylamino)-4-oxobutanoic acid[1] (35 mg, 0.22 mmol). The crude product was analyzed by RP-HPLC and NMR using an internal standard. For that purpose, 11.0 mg of dimethylmalonic acid (98% purity) was added to the reaction mixture and after syringe filtration (0.45 μm) an aliquot of the resulting solution was diluted with D_2O (to achieve a composition of 90% H_2O/10% D_2O) and analyzed by quantitative NMR with suppression of the water signal, NMR yield = **70%**, 39 mg).
After a preparative RP-HPLC (elution: gradient 2/98-80/20 acetonitrile/water containing 0.02% trifluoracetic acid, flow rate: 19 ml/min) the product was isolated as TFA salt in the form of a white solid.

^{1}H NMR (500 MHz, D_2O) δ 4.25 (dd, *J* = 7.3, 4.5 Hz, 1H), 3.25 (td, *J* = 6.7, 2.3 Hz, 2H), 3.25 (dd, *J* = 15.0, 4.5 Hz, 1H), 3.07 (dd, *J* = 15.0, 7.3 Hz, 1H), 2.68 – 2.55 (m, 4H), 3.07 (t, *J* = 6.8 Hz, 2H), 1.76 (p, *J* = 7.0 Hz, 2H) ppm.
^{13}C{H} NMR (126 MHz, D_2O) δ 179.8, 177.7, 173.6, 165.8 (q, $^2J_{C–F}$ = 35.5 Hz, CF_3**C**$O_2^–$), 119.2 (q, $^1J_{C–F}$ = 291.7 Hz, **C**$F_3CO_2^–$), 55.2, 40.8, 34.1, 33.2, 32.2, 31.7, 31.0 ppm.
^{19}F NMR (470 MHz, D_2O): δ –75.6 ppm.
HRMS (ESI): *m/z* calcd for $C_{10}H_{17}N_2O_5S$: 277.0857 $[M–H]^–$; found 277.0858.
IR (solution in MeCN, cm^{-1}): 3321, 2614, 1786, 1744, 1689, 1589, 1207, 1178, 957.

***S*-(3-hydroxy-3-methylbutyl)-L-cysteine (4)**

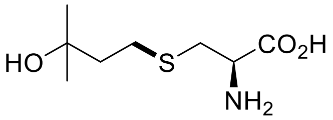

Following the general procedure described in Section 3.2.1, compound **4** was obtained from cysteine (24 mg, 0.20 mmol) and 2-methylpent-4-en-2-ol (21 mg, 0.24 mmol). The crude product was analyzed by RP-HPLC and NMR using an internal standard. For that purpose,10.7 mg of dimethylmalonic acid (98% purity) was added to the reaction mixture and after 0.45 μm syringe filtration an aliquot of the resulting solution was diluted with D_2O (to achieve a composition of 90% H_2O/10% D_2O) and analyzed by quantitative NMR with suppression of the water signal, NMR yield = **41%**, 17 mg).

[1] Synthesized according to the literature: Gülten, S. The synthesis and characterization of solvatochromic maleimide-fused *N*-allyl- and *N*-alkyl-substituted 1,4-dithiines and Diels–Alder reactions with anthracene. *J. Heterocycl. Chem.* **2010**, *47*, 188-193. Analytical data for the compound are in agreement with the literature data.

After a preparative HPLC (elution: gradient 2/98-80/20 acetonitrile/water containing 0.02% trifluoracetic acid, flow rate: 19 ml/min) the product was isolated as TFA salt.

^{1}H NMR (500 MHz, D_2O) δ 4.15 (ddd, *J* = 5.4, 4.4, 2.1 Hz, 1H), 3.19 (dd, *J* = 15.0, 4.4 Hz, 1H), 3.10 (dd, *J* = 15.0, 7.3 Hz, 1H), 2.74 – 2.58 (m, 2H), 1.79 (t, *J* = 8.4 Hz, 2H), 1.22 (s, 6H) ppm.

^{13}C{H} NMR (126 MHz, D_2O) δ 174.3, 165.6 (CF_3**C**O_2^-), 120.3 (**C**$F_3CO_2^-$), 73.9, 55.6, 45.1, 34.3, 30.4, 30.3, 29.3 ppm.

HRMS (ESI): *m/z* calcd for $C_8H_{16}NO_3S$: 206.0851 [*M–H*]$^-$; found 206.0852.

IR (solution in MeCN, cm^{-1}): 3226, 2642, 1737, 1684, 1598, 1200, 1140, 956.

Compound **4** (in a free acid form) has previously been reported;[2] however, full characterization has not been provided in the literature.

(*R*)-2-amino-21-oxo-25-((3a*S*,4*S*,6a*R*)-2-oxohexahydro-1*H*-thieno[3,4-d]imidazol-4-yl)-8,11,14,17,20-pentaoxa-4-thiapentacosanoic acid (5)

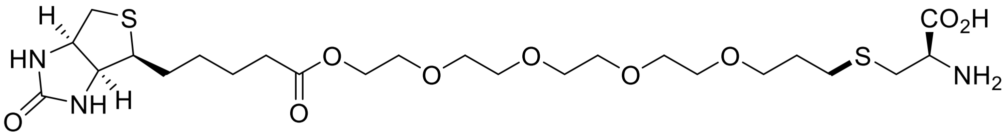

Following the general procedure described in Section 3.2.1, compound **5** was obtained from cysteine (25 mg, 0.2 mmol) and 3,6,9,12-tetraoxapentadec-14-en-1-yl 5-((3a*S*,4*S*,6a*R*)-2-oxohexahydro-1*H*-thieno[3,4-d]imidazol-4-yl)pentanoate (104 mg, 0.23 mmol).[3] The crude product was analyzed by RP-HPLC and NMR using an internal standard. For that purpose, dimethylmalonic acid (11 mg, 98% purity) was added to the reaction mixture and after 0.45 μm syringe filtration an aliquot of the resulting solution was diluted with D_2O (to achieve a composition of 90% H_2O/10% D_2O) and analyzed by quantitative NMR with suppression of the water signal, NMR yield = **98%**, 116 mg).

After a preparative HPLC (elution: gradient 2/98-80/20 acetonitrile/water containing 0.02% trifluoracetic acid, flow rate: 19 ml/min) the product was isolated as TFA salt in the form of a hygroscopic white solid.

^{1}H NMR (500 MHz, D_2O) δ 4.59 (dd, *J* = 8.0, 4.9 Hz, 1H), 4.41 (dd, *J* = 8.0, 4.5 Hz, 1H), 4.29 (dd, *J* = 7.2, 4.5 Hz, 1H), 4.27 – 4.22 (m, 2H), 3.79 – 3.74 (m, 2H), 3.73 – 3.63 (m, 13H), 3.61 (t, *J* = 6.3 Hz, 2H), 3.31 (dt, *J* = 9.6, 5.1 Hz, 1H), 3.21 (dd, *J* = 15.0, 4.5 Hz, 1H), 3.11 (dd, *J* = 15.0, 7.3 Hz, 1H), 2.98 (dd, *J* = 13.0, 5.0 Hz, 1H), 2.76 (d, *J* = 13.0 Hz, 1H), 2.67 (t, *J* = 7.1 Hz, 2H), 2.42 (t, *J* = 7.4 Hz, 2H), 1.87 (p, *J* = 6.8 Hz, 2H), 1.78 – 1.51 (m, 4H), 1.42 (p, *J* = 7.7 Hz, 2H) ppm.

^{13}C NMR (126 MHz, D_2O) δ 179.4, 173.5, 168.2, 165.7 (q, $^2J_{C\text{–}F}$ = 35.7 Hz, CF_3**C**O_2^-), 119.2 (q, $^1J_{C\text{–}F}$ = 292.5 Hz, **C**$F_3CO_2^-$), 72.60, 72.58, 72.56, 72.55, 72.5, 72.2, 72.1, 71.4, 66.7, 65.0, 63.2, 58.3, 55.2, 42.7, 36.5, 34.1, 31.3, 31.2, 30.9, 30.6, 27.1 ppm.

^{19}F NMR (470 MHz, D_2O) δ –75.6 ppm.

HRMS (ESI): *m/z* calcd for $C_{24}H_{44}N_3O_9S_2$: 582.2519 [*M+H*]$^+$; found 582.2520.

---

[2] Hendriks, W. H., Woolhouse, A. D., Tarttelin, M. F., and Moughan, P. J. Synthesis of Felinine, 2-Amino-7-hydroxy-5,5-dimethyl-4-thiaheptanoic Acid. *Bioorg. Chem*. **1995**, *23*, 89–100.

[3] Synthesized according to the literature: Weinrich, D.; Köhn, M.; Jonkheijm, P.; Westerlind, U.; Dehmelt, L.; Engelkamp, H.; Christianen, P. C. M.; Kuhlmann, J.; Maan, J. C.; Nü sse, D.; Schröder, H.; Wacker, R.; Voges, E.; Breinbauer, R.; Kunz, H.; Niemeyer, C. M.; Waldmann, H. Preparation of Biomolecule Microstructures and Microarrays by Thiol-Ene Photoimmobilization. *ChemBioChem* **2010**, *11*, 235−247. Analytical data for the compound are in agreement with the literature data.

***N*5-((*R*)-1-((carboxymethyl)amino)-3-((3-hydroxy-3-methylbutyl)thio)-1-oxopropan-2-yl)-L-glutamine (2)**

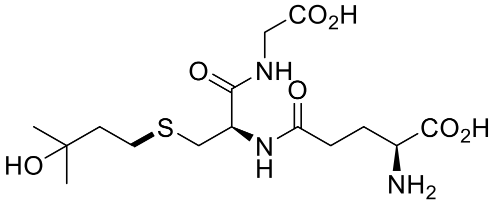

Following the general procedure described in Section 3.2.1, compound **2** was obtained from glutathione (62 mg, 0.20 mmol) and 2-methylpent-4-en-2-ol (20 mg, 0.24 mmol). The crude product was analyzed by RP-HPLC and NMR using an internal standard. For that purpose,10.4 mg of dimethylmalonic acid (98% purity) was added to the reaction mixture and after 0.45 μm syringe filtration an aliquot of the resulting solution was diluted with D_2O (to achieve a composition of 90% H_2O/10% D_2O) and analyzed by quantitative NMR with suppression of the water signal, NMR yield = **77%**, 61 mg).
After a preparative HPLC (elution: gradient 2/98-80/20 acetonitrile/water containing 0.02% trifluoracetic acid, flow rate: 19 ml/min) the product was isolated as TFA salt in the form of a white solid.

^{1}H NMR (500 MHz, D_2O) δ 4.57 (dd, *J* = 8.5, 5.3 Hz, 1H), 4.06 (t, *J* = 6.6 Hz, 1H), 4.01 (s, 2H), 3.06 (dd, *J* = 14.1, 5.3 Hz, 1H), 2.88 (dd, *J* = 14.1, 8.6 Hz, 1H), 2.68 – 2.53 (m, 4H), 2.31 – 2.15 (m, 2H), 1.83 – 1.66 (m, 2H), 1.20 (s, 6H) ppm.
^{13}C{H} NMR (126 MHz, D_2O) δ 177.3, 175.8, 175.8, 174.7, 165.8 (q, $^2J_{C\text{–}F}$ = 35.6 Hz, CF_3**C**O_2^-), 119.3 (q, $^1J_{C\text{–}F}$ = 291.7 Hz, **C**$F_3CO_2^-$), 74.1, 56.0, 55.3, 45.4, 44.0, 35.7, 33.9, 30.53, 30.47, 29.5, 28.5 ppm.
^{19}F NMR (470 MHz, D_2O): δ –75.6 ppm.
HRMS (ESI): *m/z* calcd for $C_{15}H_{28}N_3O_7S$: 394.1648 $[M+H]^+$; found 394.1647.
IR (KBr pellet, cm^{-1}): 3302, 2976, 1739, 1667,3 1537, 1421,1200, 1140, 722.

***N*5-((*R*)-3-((4-carboxybutyl)thio)-1-((carboxymethyl)amino)-1-oxopropan-2-yl)-L-glutamine (6)**

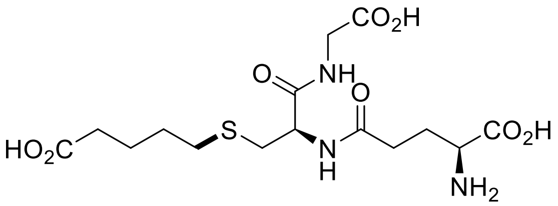

Following the general procedure described in Section 3.2.1, compound **6** was obtained from glutathione (62 mg, 0.20 mmol) and pent-4-enoic acid (23 mg, 0.22 mmol). The crude product was analyzed by RP-HPLC and NMR using an internal standard. For that purpose, dimethylmalonic acid (11 mg, 98% purity) was added to the reaction mixture and after 0.45 μm syringe filtration an aliquot of the resulting solution was diluted with D_2O (to achieve a composition of 90% H_2O/10% D_2O) and analyzed by quantitative NMR with suppression of the water signal, NMR yield = **94%**, 77 mg).
After a preparative HPLC (elution: gradient 2/98-80/20 acetonitrile/water containing 0.02% trifluoracetic acid, flow rate: 19 ml/min) the product was isolated as TFA salt in the form of a white solid.

^{1}H NMR (600 MHz, D_2O) δ 4.57 (dd, *J* = 8.7, 5.2 Hz, 1H), 4.03 – 3.98 (s+t, 3H), 3.05 (dd, *J* = 14.1, 5.2 Hz, 1H), 2.87 (dd, *J* = 14.1, 8.7 Hz, 1H), 2.67 – 2.52 (m, 4H), 2.40 (t, *J* = 7.2 Hz, 2H), 2.28 – 2.16 (m, 2H), 1.72 – 1.65 (m, 2H), 1.65 – 1.58 (m, 2H) ppm.
^{13}C{H} NMR (126 MHz, D_2O) 181.5, 177.2, 175.8, 175.8, 174.6, 165.8 (q, $^2J_{C-F}$ = 35.6 Hz, $CF_3\mathbf{C}O_2^-$), 119.3 (q, $^1J_{C-F}$ = 291.7 Hz, $\mathbf{C}F_3CO_2^-$), 56.0, 55.3, 44.1, 36.2, 35.7, 34.1, 33.9, 30.9, 28.5, 26.2 ppm.
HRMS (ESI): *m/z* calcd for $C_{15}H_{26}N_3O_8S$: 408.1441 $[M+H]^+$; found 408.1443.
IR (KBr pellet, cm^{-1}): 3087, 2943, 1731, 1665, 1536, 1415, 1347, 1195, 1138, 722.
Compound **6** (in a free acid form) has previously been reported,[4] however, there is no full characterization provided in the literature.

***N*5-((*R*)-1-((carboxymethyl)amino)-3-((3-(3-carboxypropanamido)propyl)thio)-1-oxopropan-2-yl)-L-glutamine (7)**

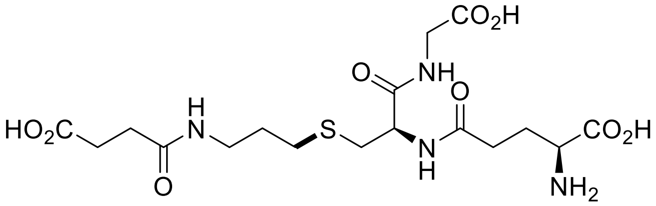

Following the general procedure described in Section 3.2.1, compound **7** was obtained from glutathione (62 mg, 0.20 mmol) and 4-(allylamino)-4-oxobutanoic acid[1] (35 mg, 0.22 mmol). The crude product was analyzed by RP-HPLC and NMR using an internal standard. For that purpose, dimethylmalonic acid (10 mg, 98% purity) was added to the reaction mixture and after 0.45 μm syringe filtration an aliquot of the resulting solution was diluted with D_2O (to achieve a composition of 90% H_2O/10% D_2O) and analyzed by quantitative NMR with suppression of the water signal, NMR yield = **91%**, 85 mg).
After a preparative HPLC (elution: gradient 2/98-80/20 acetonitrile/water containing 0.02% trifluoracetic acid, flow rate: 19 ml/min) the product was isolated as TFA salt in the form of a white solid.

^{1}H NMR (500 MHz, D_2O) δ 4.57 (dd, *J* = 8.6, 5.2 Hz, 1H), 4.01 (s, 2H), 3.95 (t, *J* = 6.5 Hz, 1H), 3.27 (t, *J* = 6.7 Hz, 2H), 3.04 (dd, *J* = 14.0, 5.2 Hz, 1H), 2.87 (dd, *J* = 14.0, 8.6 Hz, 1H), 2.69 – 2.64 (m, 2H), 2.62 – 2.50 (m, 6H), 2.20 (qd, *J* = 7.4, 2.3 Hz, 2H), 1.78 (p, *J* = 7.1 Hz, 2H) ppm.
^{13}C{H} NMR (126 MHz, D_2O) δ 182.5, 180.4, 180.2, 178.6, 178.5, 178.2, 58.7, 46.8, 43.6, 38.3, 36.6, 35.9, 34.9, 34.4, 33.8, 31.4. ppm. One carbon signal is missing due to overlap, no TFA anion signals visible.
^{19}F NMR (470 MHz, D_2O): –75.6 ppm.
HRMS (ESI): *m/z* calcd for $C_{17}H_{29}N_4O_9S$: 465.1655 $[M+H]^+$; found 465.1660.
IR (KBr pellet, cm^{-1}): 3291, 3075, 2936, 1718, 1666, 1548, 1414, 1202, 1138.

[4] Colak, B.; Da Silva, J. C. S.; Soares, T. A.; Gautrot, J. E. Impact of the Molecular Environment on Thiol–Ene Coupling For Biofunctionalization and Conjugation. *Bioconjugate Chem*. **2016**, *27*, 2111−2123.

(3*S*,12*R*,17*S*)-17-amino-12-((carboxymethyl)carbamoyl)-5,14-dioxo-6-oxa-10-thia-4,13-diazaheptadecane-1,3,17-tricarboxylic acid (8)

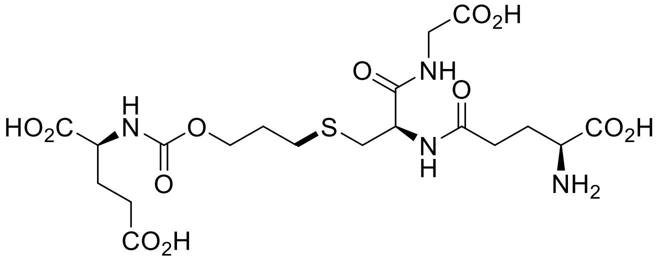

Following the general procedure described in Section 3.2.1, compound **8** was obtained from glutathione (62mg, 0.20 mmol) and ((allyloxy)carbonyl)-L-glutamic acid[5] (51 mg, 0.22 mmol). The crude product was analyzed by RP-HPLC and NMR using an internal standard. For that purpose, dimethylmalonic acid (10 mg, 98% purity) was added to the reaction mixture and after 0.45 μm syringe filtration an aliquot of the resulting solution was diluted with D_2O (to achieve a composition of 90% H_2O/10% D_2O) and analyzed by quantitative NMR with suppression of the water signal, NMR yield = **68%**, 73 mg). After a preparative HPLC (elution: gradient 2/98-80/20 acetonitrile/water containing 0.02% trifluoracetic acid, flow rate: 19 ml/min) the product was isolated as TFA salt.

^{1}H NMR (500 MHz, D_2O) δ 4.57 (dd, *J* = 8.6, 5.2 Hz, 1H), 4.23 (dd, *J* = 9.2, 4.9 Hz, 1H), 4.15 (t, *J* = 5.8 Hz, 2H), 4.05 – 3.98 (t+s, 3H), 3.04 (dd, *J* = 14.1, 5.3 Hz, 1H), 2.87 (dd, *J* = 14.0, 8.7 Hz, 1H), 2.70 – 2.62 (m, 2H), 2.62 – 2.47 (m, 4H), 2.29 – 2.14 (m, 3H), 2.03 – 1.95 (m, 1H), 1.91 (p, *J* = 6.7 Hz, 2H) ppm.
^{13}C{H} NMR (126 MHz, D_2O) δ 180.1, 178.6, 177.3, 175.8, 175.7, 175.0, 165.9 (q, $^2J_{C\text{–}F}$ = 35.5 Hz, $CF_3\mathbf{C}O_2^-$), 161.1, 119.3 (q, $^1J_{C\text{–}F}$ = 291.8 Hz, $\mathbf{C}F_3CO_2^-$), 67.1, 56.2, 56.0, 55.6, 44.1, 35.7, 33.9, 33.0, 31.1, 30.9, 28.9, 28.5 ppm.

Analytical data for compound **8** (in TFA salt form) are in agreement with the literature data reported for free acid form of compound **8**.[6]

(2*S*,11*R*,16*S*)-16-amino-11-((carboxymethyl)carbamoyl)-2-(hydroxymethyl)-4,13-dioxo-5-oxa-9-thia-3,12-diazaheptadecanedioic acid (9)

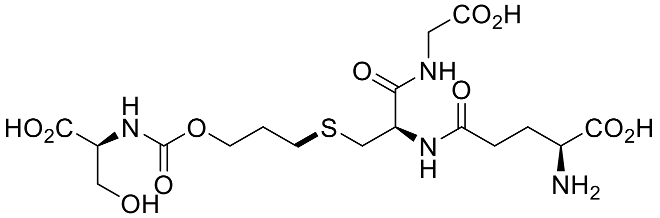

Following the general procedure described in Section 3.2.1, compound **9** was obtained from glutathione (62 mg, 0.20 mmol) and ((allyloxy)carbonyl)-L-serine[5] (42 mg, 0.22 mmol). The crude product was analyzed by RP-HPLC and NMR using an internal standard. For that purpose, dimethylmalonic acid (10 mg, 98% purity) was added to the reaction mixture and after 0.45 μm syringe filtration an aliquot of the

[5] Synthesized according to the literature: Yan, B.; Li, W.; Hackenberger, C. P. R. A silyl ether-protected building block for O-GlcNAcylated peptide synthesis to enable one-pot acidic deprotection. *Org. Biomol. Chem.* **2021**, *19*, 8014−8017. Analytical data for the compound are in agreement with the literature data.
[6] Tyson, E. L.; Niemeyer, Z. L.; Yoon, T. P. Redox Mediators in Visible Light Photocatalysis: Photocatalytic Radical Thiol–Ene Additions. *J. Org. Chem.* **2014**, *79*, 1427–1436.

resulting solution was diluted with D_2O (to achieve a composition of 90% H_2O/10% D_2O) and analyzed by quantitative NMR with suppression of the water signal, NMR yield = **53%**, 52 mg).
After a preparative HPLC (elution: gradient 2/98-80/20 acetonitrile/water containing 0.02% trifluoracetic acid, flow rate: 19 ml/min) the product was isolated as TFA salt.

^{1}H NMR (500 MHz, D_2O) δ 4.58 (dd, *J* = 8.6, 5.2 Hz, 1H), 4.39 – 4.31 (m, 1H), 4.24 – 4.14 (m, 2H), 4.01 (s, 2H), 3.96 – 3.87 (m, 3H), 3.07 (dd, *J* = 14.1, 5.2 Hz, 1H), 2.89 (dd, *J* = 14.1, 8.6 Hz, 1H), 2.74 – 2.63 (m, 2H), 2.63 – 2.49 (m, 2H), 2.25 – 2.15 (m, 2H), 1.93 (p, *J* = 6.7 Hz, 2H) ppm.
^{13}C{H} NMR (126 MHz, D_2O) δ 180.3, 179.8, 178.7, 178.55, 178.45, 163.9, 69.8, 66.9, 61.8, 58.9, 58.7, 46.8, 38.4, 36.7, 33.8, 33.6, 31.4 ppm.

Analytical data for compound **9** (in TFA salt form) are in agreement with the literature data reported for free acid form of compound **9**.[6]

(18*R*,23*S*)-23-amino-18-((carboxymethyl)carbamoyl)-1-hydroxy-20-oxo-3,6,9,12-tetraoxa-16-thia-19-azatetracosan-24-oic acid (10)

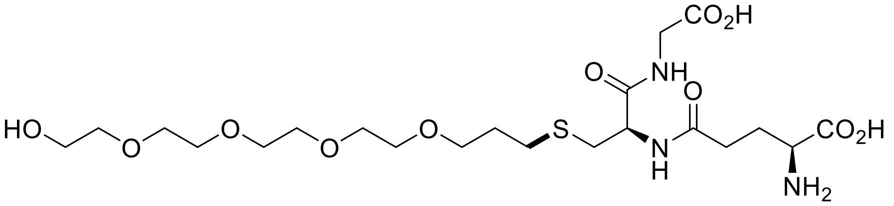

Following the general procedure described in Section 3.2.1, compound **10** was obtained from glutathione (0.20 mmol) and 3,6,9,12-tetraoxapentadec-14-en-1-ol[7] (0.22 mmol). The crude product was analyzed by RP-HPLC and NMR using an internal standard. For that purpose, dimethylmalonic acid (10 mg, 98% purity) was added to the reaction mixture and after 0.45 µm syringe filtration an aliquot of the resulting solution was diluted with D_2O (to achieve a composition of 90% H_2O/10% D_2O) and analyzed by quantitative NMR with suppression of the water signal, NMR yield = **95%**, 103 mg).
After a preparative HPLC (elution: gradient 2/98-80/20 acetonitrile/water containing 0.02% trifluoracetic acid, flow rate: 19 ml/min) the product was isolated as TFA salt.

^{1}H NMR (500 MHz, D_2O) δ 4.57 (dd, *J* = 8.6, 5.3 Hz, 1H), 4.06 (t, *J* = 6.6 Hz, 1H), 4.01 (s, 2H), 3.76 – 3.58 (m, 18H), 3.05 (dd, *J* = 14.1, 5.3 Hz, 1H), 2.88 (dd, *J* = 14.1, 8.7 Hz, 1H), 2.70 – 2.63 (m, 2H), 2.63 – 2.53 (m, 2H), 2.31 – 2.14 (m, 2H), 1.86 (p, *J* = 6.9 Hz, 2H) ppm.
^{13}C{H} NMR (126 MHz, D_2O) δ 177.3, 175.80, 175.76, 174.7, 165.9 (q, $^2J_{C\text{–}F}$ = 35.1 Hz, CF_3**C**O_2^-), 119.3 (q, $^1J_{C\text{–}F}$ = 292.0 Hz, **C**$F_3CO_2^-$), 74.7, 72.6, 72.53, 72.51, 72.49, 72.4, 72.2, 63.3, 56.0, 55.3, 44.0, 35.7, 33.9, 31.4, 31.1, 28.5 ppm.

Analytical data for compound **10** (in TFA salt form) are in agreement with the literature data reported for free acid form of compound **10**.[6]

[7] Synthesized according to the literature: Xu, W. Z.; Zhang, X.; Kadla, J. F. Design of Functionalized Cellulosic Honeycomb Films: Site-Specific Biomolecule Modification via “Click Chemistry”. *Biomacromolecules* **2012**, *13*, 350−357. Analytical data for the compound are in agreement with the literature data.

***N*5-((*R*)-1-((carboxymethyl)amino)-1-oxo-3-((3-((5-((3a*S*,4*S*,6a*R*)-2-oxohexahydro-1*H*-thieno[3,4-d]imidazol-4-yl)pentanoyl)oxy)propyl)thio)propan-2-yl)-L-glutamine (11)**

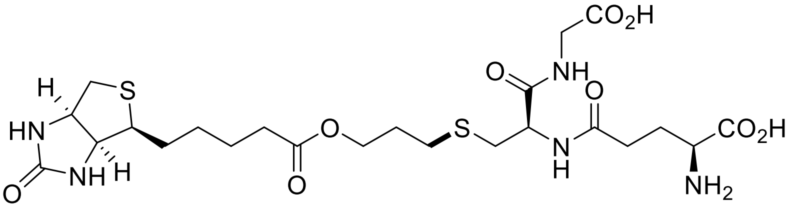

Following the general procedure described in Section 3.2.1, compound **11** was obtained from glutathione (62 mg, 0.2 mmol) allyl 5-((3a*S*,4*S*,6a*R*)-2-oxohexahydro-1*H*-thieno[3,4-d]imidazol-4-yl)pentanoate[8] (63 mg, 0.22 mmol). The crude product was analyzed by RP-HPLC and NMR using an internal standard. For that purpose, dimethylmalonic acid (10 mg, 98% purity) was added to the reaction mixture and after 0.45 μm syringe filtration an aliquot of the resulting solution was diluted with D_2O (to achieve a composition of 90% H_2O/10% D_2O) and analyzed by quantitative NMR with suppression of the water signal, NMR yield = **74%**, 88 mg).

After a preparative HPLC (elution: gradient 2/98-80/20 acetonitrile/water containing 0.02% trifluoracetic acid, flow rate: 19 ml/min) the product was isolated as TFA salt in the form of a white solid.

^{1}H NMR (600 MHz, DMSO-*d*$_6$) δ 8.42 (t, *J* = 5.9 Hz, 1H), 8.27 (d, *J* = 8.5 Hz, 1H), 6.42 (s, 1H), 6.37 (s, 1H), 4.47 (td, *J* = 8.9, 4.8 Hz, 1H), 4.31 (dd, *J* = 7.7, 5.0 Hz, 1H), 4.17 – 4.11 (m, 1H), 4.06 (t, *J* = 6.4 Hz, 2H), 3.92 (t, *J* = 6.4 Hz, 1H), 3.81 – 3.71 (m, 2H), 3.14 – 3.06 (m, 1H), 2.84 (ddd, *J* = 22.8, 13.1, 5.0 Hz, 2H), 2.67 – 2.51 (m, 4H), 2.44 – 2.34 (m, 1H), 2.35 – 2.25 (m, 3H), 2.10 – 1.91 (m, 2H), 1.81 (pd, *J* = 6.7, 2.5 Hz, 2H), 1.66 – 1.41 (m, 4H), 1.40 – 1.25 (m, 2H) ppm.

^{13}C{H} NMR (126 MHz, DMSO-*d*$_6$) δ 173.0, 171.1, 171.0, 170.8, 170.7, 162.8, 158.3 (q, $^2J_{C\text{–}F}$ = 33.9 Hz, $CF_3\mathbf{C}O_2^-$), 117.0 (q, $^1J_{C\text{–}F}$ = 292.0 Hz, $\mathbf{C}F_3CO_2^-$) 62.5, 61.1, 59.3, 55.4, 52.2, 51.7, 40.8, 33.64, 33.55, 33.3, 30.7, 28.2, 28.1, 28.0, 27.8, 26.1, 24.5 ppm.

Analytical data for compound **11** (in TFA salt form) are in agreement with the literature data reported for free acid form of compound **11**.[6]

(24*R*,29*S*)-29-amino-24-((carboxymethyl)carbamoyl)-5,26-dioxo-1-((3a*S*,4*S*,6a*R*)-2-oxohexahydro-1*H*-thieno[3,4-d]imidazol-4-yl)-6,9,12,15,18-pentaoxa-22-thia-25-azatriacontan-30-oic acid (12)

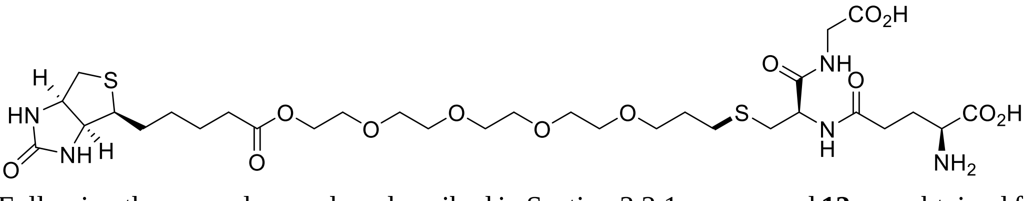

Following the general procedure described in Section 3.2.1, compound **12** was obtained from glutathione (62 mg, 0.20 mmol) and 3,6,9,12-tetraoxapentadec-14-en-1-yl 5-((3a*S*,4*S*,6a*R*)-2-oxohexahydro-1*H*-thieno[3,4-d]imidazol-4-yl)pentanoate (102 mg, 0.22 mmol).[3] The crude product was analyzed by RP-HPLC and NMR using an internal standard. For that purpose, dimethylmalonic acid (10 mg, 98% purity) was added to the reaction mixture and after 0.45 μm syringe filtration an aliquot of the resulting solution

[8] Synthesized according to the literature: Merbouh, N.; Wallner, F.; Cociorva, O. M.; Seeberger, P. H. 3-Mercaptopropanol as a Traceless Linker for Chemical and Enzymatic Synthesis of Oligosaccharides. *Org. Lett.* **2007**, *9*, 651– 653. Analytical data for the compound are in agreement with the literature data.

was diluted with D_2O (to achieve a composition of 90% H_2O/10% D_2O) and analyzed via quantitative NMR with suppression of the water signal, NMR yield = **93%**, 144 mg).
After a preparative HPLC (elution: gradient 2/98-80/20 acetonitrile/water containing 0.02% trifluoracetic acid, flow rate: 19 ml/min) the product was isolated as TFA salt.

^{1}H NMR (500 MHz, D_2O) δ 4.66 – 4.54 (m, 2H), 4.43 (dd, *J* = 7.9, 4.5 Hz, 1H), 4.32 – 4.25 (m, 2H), 4.09 – 3.99 (m, 3H), 3.83 – 3.76 (m, 2H), 3.75 – 3.65 (m, 13H), 3.63 (t, *J* = 6.3 Hz, 2H), 3.34 (dt, *J* = 9.8, 5.1 Hz, 1H), 3.06 (dd, *J* = 14.0, 5.3 Hz, 1H), 3.00 (dd, *J* = 13.1, 5.0 Hz, 1H), 2.89 (dd, *J* = 14.0, 8.7 Hz, 1H), 2.79 (d, *J* = 13.0 Hz, 1H), 2.72 – 2.63 (m, 2H), 2.63 – 2.52 (m, 2H), 2.45 (t, *J* = 7.4 Hz, 2H), 2.23 (p, *J* = 7.1 Hz, 2H), 1.88 (p, *J* = 6.8 Hz, 2H), 1.80 – 1.54 (m, 4H), 1.45 (p, *J* = 7.5 Hz, 2H) ppm.
^{13}C{H} NMR (126 MHz, D_2O) δ 182.2, 179.9, 178.5, 178.4, 177.5, 170.9, 168.5 (q, $^2J_{C-F}$ = 35.3 Hz, $CF_3\mathbf{C}O_2^-$), 121.9 (q, $^1J_{C-F}$ = 291.6 Hz, $\mathbf{C}F_3CO_2^-$), 75.28, 75.26, 75.23, 75.19, 74.89, 74.87, 74.1, 69.4, 67.7, 65.9, 60.9, 58.7, 58.2, 46.7, 45.3, 39.2, 38.4, 36.6, 34.1, 33.8, 33.5, 33.3, 31.2, 29.7 ppm.
^{19}F NMR (470 MHz, D_2O): δ –75.6 ppm.
HRMS (ESI): *m/z* calcd for $C_{32}H_{54}N_5O_{13}S_2$: 768.3160 $[M+H]^+$; found 768.3154.

S-(3-(3-carboxypropanamido)propyl)-L-homocysteine (13)

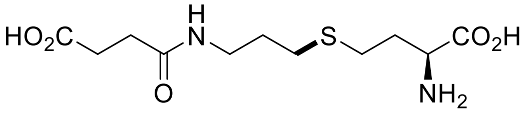

Following the general procedure described in Section 3.2.1, compound **13** was obtained from homocysteine (27 mg, 0.20 mmol) and 4-(allylamino)-4-oxobutanoic acid[3] (35 mg, 0.22 mmol). The crude product was analyzed by RP-HPLC and NMR using an internal standard. For that purpose, dimethylmalonic acid (10 mg, 98% purity) was added to the reaction mixture and after 0.45 μm syringe filtration an aliquot of the resulting solution was diluted with D_2O (to achieve a composition of 90% H_2O/10% D_2O) and analyzed by quantitative NMR with suppression of the water signal, NMR yield = **70%**, 41 mg).
After a preparative HPLC (elution: gradient 2/98-80/20 acetonitrile/water containing 0.02% trifluoracetic acid, flow rate: 19 ml/min) the product was isolated as TFA salt.

^{1}H NMR (500 MHz, D_2O) δ 4.15 – 4.09 (m, 1H), 3.29 (t, *J* = 6.7 Hz, 2H), 2.71 (t, *J* = 7.4 Hz, 2H), 2.68 – 2.64 (m, 2H), 2.60 (t, *J* = 7.3 Hz, 2H), 2.53 (t, *J* = 6.7 Hz, 2H), 2.32 – 2.22 (m, 1H), 2.22 – 2.11 (m, 1H), 1.79 (p, *J* = 6.9 Hz, 2H) ppm.
^{13}C{H} NMR (126 MHz, D_2O) δ 179.9, 177.7, 175.3, 165.9 (q, $^2J_{C-F}$ = 35.4 Hz), 119.3 (q, $^1J_{C-F}$ = 291.8 Hz), 55.3, 41.0, 33.3, 32.6, 32.3, 31.1, 30.9, 29.4 ppm.
^{19}F NMR (151 MHz, D_2O): –75.6 ppm.
HRMS (ESI): *m/z* calcd for $C_{11}H_{21}N_2O_5S$: 293.1171 $[M+H]^+$; found 293.1174.
IR (solution in MeCN, cm^{-1}): 3294, 2639, 2615, 1784, 1740, 1694, 1581, 1200, 1178.

2-((3-(3-carboxypropanamido)propyl)thio)ethan-1-aminium chloride (14)

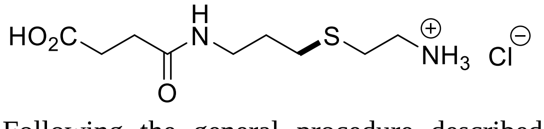

Following the general procedure described in Section 3.2.1, compound **14** was obtained from cysteaminium chloride (23 mg, 0.20 mmol) and 4-(allylamino)-4-oxobutanoic acid[3] (35 mg, 0.22 mmol). The crude product was analyzed by RP-HPLC and NMR using an internal standard. For that purpose, dimethylmalonic acid (10.8 mg , 98% purity) was added to the reaction mixture and after 0.45

μm syringe filtration an aliquot of the resulting solution was diluted with D_2O (to achieve a composition of 90% H_2O/10% D_2O) and analyzed by quantitative NMR with suppression of the water signal, NMR yield = **24%**, 14 mg).
After a preparative HPLC (elution: gradient 2/98-80/20 acetonitrile/water containing 0.02% trifluoracetic acid, flow rate: 19 ml/min) the product was isolated as TFA salt.

^{1}H NMR (500 MHz, D_2O) 3.29 (t, *J* = 6.7 Hz, 2H), 3.21 (t, *J* = 6.7 Hz, 2H), 2.85 (t, *J* = 6.6 Hz, 2H), 2.67 (t, *J* = 6.6 Hz, 2H), 2.60 (t, *J* = 7.3 Hz, 2H), 2.53 (t, *J* = 6.7 Hz, 2H), 1.80 (p, *J* = 6.9 Hz, 2H) ppm.
^{13}C{H} NMR (126 MHz, D_2O) δ 179.9, 177.7, 165.9 (q, $^2J_{C\text{–}F}$ = 36.0 Hz), 119.3 (q, $^1J_{C\text{–}F}$ = 291.6 Hz), 41.2, 40.9, 33.2, 32.2, 31.1 (two overlapped carbon signals), 30.8 ppm.
^{19}F NMR (470 MHz, D_2O): –75.6 ppm.
HRMS (ESI): *m/z* calcd for $C_9H_{19}N_2O_3S$: 235.1116 $[M+H]^+$; found 235.1120.
IR (solution in MeCN, cm^{-1}): 3374, 3233, 2641, 2612, 1729, 1693, 1201, 1181, 1136.

((*R*)-3-((3-hydroxy-3-methylbutyl)thio)-2-methylpropanoyl)-D-proline (15)

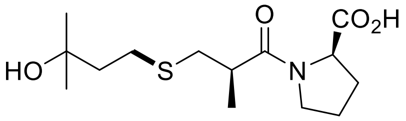

Following the general procedure described in Section 3.2.1, compound **15** was obtained from captopril (44 mg, 0.20 mmol) and and 2-methylpent-4-en-2-ol (22 mg, 0.25 mmol). The crude product was analyzed by RP-HPLC and NMR using an internal standard. For that purpose, dimethylmalonic acid (11 mg, 98% purity) was added to the reaction mixture and after 0.45 μm syringe filtration an aliquot of the resulting solution was diluted with D_2O (to achieve a composition of 90% H_2O/10% D_2O) and analyzed by quantitative NMR with suppression of the water signal, NMR yield = **61%**, 37 mg).
After a preparative HPLC (elution: gradient 2/98-80/20 acetonitrile/water containing 0.02% trifluoracetic acid, flow rate: 19 ml/min) the product was isolated.

Captopril is known to exist in equilibrium of two conformers,[9] below only signals of the major conformer of the product **15** are given.
^{1}H NMR (500 MHz, D_2O) δ 4.43 (dd, *J* = 8.7, 4.0 Hz, 1H), 3.73 (t, *J* = 6.6 Hz, 2H), 3.16 – 2.96 (m, 1H), 2.76 (dd, *J* = 13.3, 9.6 Hz, 1H), 2.64 (dd, *J* = 13.3, 5.2 Hz, 1H), 2.62 – 2.56 (m, 2H), 2.38 – 2.24 (m, 1H), 2.08 – 1.98 (m, 3H), 1.78 – 1.67 (m, 2H), 1.19 (s, 6H), 1.15 (d, *J* = 6.7 Hz, 3H) ppm.
^{13}C{H} NMR (151 MHz, D_2O) δ 179.6, 179.0, 74.1, 62.1, 50.8, 45.7, 41.2, 37.5, 31.9, 30.54, 30.48, 29.7, 27.3, 19.2 ppm.
HRMS (APCI): *m/z* calcd for $C_{14}H_{24}NO_4S$: 302.1426 $[M\text{–}H]^+$; found 302.1428.

[9] Casy, A. F.; Dewar, G. H. Captopril and its probable contaminants: NMR and MS features of analytical value, *J. Pharm. Biomed. Anal.* **1994**, 12, 855-861.

CoA conjugate (16)

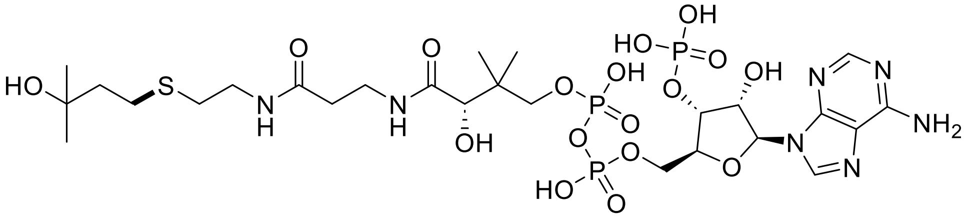

Following the general procedure described in Section 3.2.1, compound **16** was obtained from coenzyme A (77 mg, 85% purity, 0.085 mmol) and 2-methylpent-4-en-2-ol (26 mg, 0.30 mmol). The crude product was analyzed by RP-HPLC and NMR using an internal standard. For that purpose, dimethylmalonic acid (10 mg, 98% purity) was added to the reaction mixture and after 0.45 µm syringe filtration an aliquot of the resulting solution was diluted with D_2O (to achieve a composition of 90% H_2O/10% D_2O) and analyzed by quantitative NMR with suppression of the water signal, NMR yield = **85%**, 61 mg). After a preparative HPLC (elution: gradient 2/98-80/20 acetonitrile/water containing 0.02% trifluoracetic acid, flow rate: 19 ml/min) the product was isolated as TFA salt.

^{1}H NMR (500 MHz, D_2O) 8.59 (s, 1H), 8.40 (s, 1H), 6.17 (d, J = 5.8 Hz, 1H), 4.92 (td, J = 5.1, 2.6 Hz, 1H), 4.86 (td, J = 5.6, 5.2, 1.3 Hz, 1H), 4.62 – 4.54 (m, 1H), 4.32 – 4.17 (m, 2H), 3.97 (s, 1H), 3.86 (dd, J = 9.8, 4.6 Hz, 1H), 3.64 (dd, J = 9.8, 4.6 Hz, 1H), 3.44 (t, J = 6.4 Hz, 2H), 3.32 (t, J = 6.7 Hz, 2H), 2.63 (t, J = 6.7 Hz, 2H), 2.57 – 2.50 (m, 2H), 2.44 (t, J = 6.5 Hz, 2H), 1.76 – 1.62 (m, 2H), 1.15 (s, 6H), 0.91 (s, 3H), 0.80 (s, 3H) ppm.
^{13}C{H} NMR (126 MHz, D_2O) δ 180.3, 179.5, 168.3 (q, $^2J_{C\text{–}F}$ = 36.0 Hz), 155.4, 154.1, 150.3, 148.1, 124.1, 121.8 (q, $^1J_{C\text{–}F}$ = 291.1 Hz), 93.1, 80.1, 80.1 (d, $J_{C\text{–}P}$ = 4.9 Hz), 79.8, 79.3 (d, $J_{C\text{–}P}$ = 4.9 Hz), 77.9 (d, $J_{C\text{–}P}$ = 5.7 Hz, two overlapped carbon signals), 76.8, 70.8 (d, $J_{C\text{–}P}$ = 4.7 Hz), 48.2, 44.1, 44.0 (d, $J_{C\text{–}P}$ = 7.9 Hz), 41.1, 40.9, 35.9, 33.1 (two overlapped carbon signals), 31.4, 26.3, 24.0 ppm.
^{19}F NMR (470 MHz, D_2O): –75.7 ppm.
^{31}P NMR (202 MHz, D_2O) δ –0.81, –11.29, –11.77 ppm.
HRMS (ESI): *m/z* calcd for $C_{26}H_{47}N_7O_{17}P_3S$: 854.1962 $[M+H]^+$; found 854.1974.

3.3 Amino acids tolerance screening with EAA

The robustness of the developed thiol-ene reaction conditions in a complex mixture containing multiple amino acid residues was tested by performing the model reaction with the addition of a commercially available dietary supplement consisting of the following amounts of 10 essential amino acids (EAA).

Entry	Amino acid	Composition [mg/1 g]	Amount added [equiv.]
1	Leu	300 mg	1.79
2	Val	150 mg	1.00
3	Ile	150 mg	0.89
4	Lys HCl	135 mg	0.58
5	Thr	70 mg	0.46
6	Phe	70 mg	0.33
7	Met	45 mg	0.24
8	Arg	40 mg	0.18
9	His	30 mg	0.15
10	Trp	10 mg	0.04

Experiment description: two parallel reactions for the synthesis of compound **2** were set up. The first one according to the general procedure described in Section 3.2.1 using glutathione (62 mg, 0.20 mmol) and 2-methylpent-4-en-2-ol (52 mg, 22 mmol), and the second one using glutathione (62 mg, 0.20 mmol), 2-methylpent-4-en-2-ol (54 mg, 22 mmol) and 156 mg of commercially available essential amino acids mixture with the composition specified in the table above. The crude product in the two reactions was analyzed by RP-HPLC and NMR using an internal standard. For that purpose, dimethylmalonic acid (10.6 mg and 10.5 mg, 98% purity) were added to the reaction mixtures, correspondingly, and after 0.45 µm syringe filtration aliquots of the resulting solutions were diluted with D_2O (to achieve a composition of 90% H_2O/10% D_2O) and analyzed by quantitative NMR with suppression of the water signal. NMR yield for the reference experiment (without EAA) = **87**%, NMR yield for the experiment with EAA addition = **99**%.

3.4 Optimization of the red light-induced radical thiol-ene reaction of disulfide with olefin

As a part of development of the conditions compatible with proteins containing disulfide bonds, reactions of oxidized glutathione (GSSG) with common disulfide reducing agents such as dithiothreitol (DTT) or tris(2-carboxyethyl)phosphine (TCEP) were performed following the thiol-ene reaction.

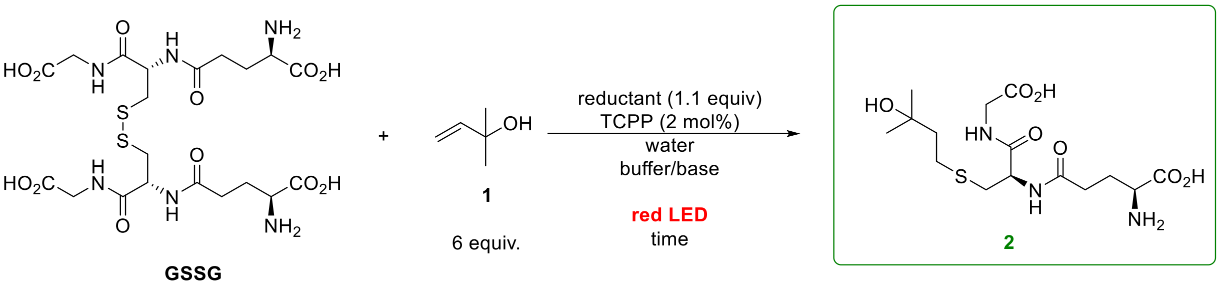

Entry	Reductant	Buffer/base	Time	HPLC results
				**2**
1	none	NH_4OAc (0.15 M)	4 h	0%
2	TCEP-HCl	NH_4OAc (1 M)	4 h	55%
3	TCEP-HCl	KOAc (3 M)	4 h	64%
4	TCEP-HCl	NaOH (pH = 8-9)	4 h	12%
5	DTT	NH_4OAc (1 M)	4 h	64%
6[a]	DTT	NH_4OAc (1 M)	4 h	51%
			24 h	64%

[a] *Kessil lamp used.*

Conclusions: *prior disulfide reduction is necessary for the thiol-ene reaction to proceed, DTT and TCEP are comparably effective reducing agents in this context.*

3.5 Bioconjugation reaction with HSA

3.5.1 Reduction of human serum albumin (HSA)

Following a literature protocol,[10] human serum albumin (HSA; purchased from Sigma Aldrich; product code A9731, 200 µL, 373 µM) dissolved in PBS (137 mM NaCl, 10 mM phosphate, 2.7 mM KCl; pH

[10] Seki, H.; Walsh, S. J.; Bargh, J. D.; Parker, J. S.; Carroll, J.; Spring. D. R. Rapid and Robust Cysteine Bioconjugation with Vinylheteroarenes. *Chem. Sci*. **2021**, *12*, 9060–9068.

7.4) and dithiothreitol (DTT, 11.8 μL, 13 mM) was added to the mixture, vortexed and incubated at 37 °C for 2 h. Excess reagents were removed by ultrafiltration (3x 50,000 MWCO, Amicon® Ultra, Millipore), yielding a reduced HSA protein with a free Cys-34, whose mass was confirmed by LC-MS-QTOF (data not shown).

3.5.2 Bioconjugation of HAS

To a reduced HSA solution in 0.5 M $NaHCO_3$/Na_2CO_3 (1200 μL, 37.3 μM) were added 3,6,9,12-tetraoxapentadec-14-en-1-yl-5-((3aS,4S,6aR)-2-oxohexahydro-1H-thieno[3,4-d]imidazol-4-yl)penta-noate (allyl-glycol-biotin, 50 eq., 143 μL, 15.7 mM) and TCPP (5 mol%, 45.7 μL, 2.5 mM), previously dissolved in 0.5 M $NaHCO_3$/Na_2CO_3 separately and sonicated for 1h. The mixture was stirred and irradiated (660 nm, 50% power of the UOSlab Miniphoto photoreactor) for 24h.

3.5.3 Dot-blot analysis of HSA conjugate

Samples of the reaction mixture (1 μL) were placed on a nitrocellulose membrane and left to dry for 10 min at room temperature. The membrane was blocked in phosphate buffered saline with 0.1% Tween 20 (PBST) and 5% milk powder for 5 min at room temperature and subsequently washed with PBST and PBS for 5 min at room temperature. ExtrAvidin®-Peroxidase was diluted 1:2000 in PBST and incubated with the membrane for 5 min, followed by washing with PBST for 5 min at room temperature and detected with chemiluminiscent substrate (SuperSignal™ West Femto Maximum Sensitivity, Thermo Scientific).

3.5.4 Purification and analysis of HSA conjugate

Prior to purification, the samples were concentrated by ultrafiltration (10,000 MWCO, Vivaspin® 2, Sartorius) and centrifuged for 5 min at 17,000 rcf to remove potential precipitates. Purification was performed in PBS at a flow rate 0.4 ml/min with an injection volume of 350 μL. The fractions of interest were pooled together and concentrated by ultrafiltration (10000 MWCO, Vivaspin® 2, Sartorius).
The fraction containing a potential product (the first peak in the chromatogram below) was analyzed by MALDI-TOF MS.

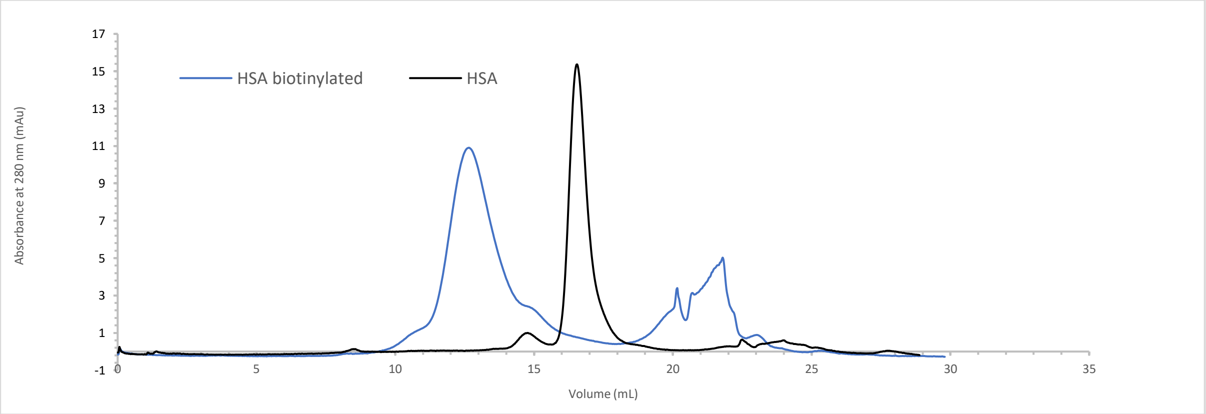

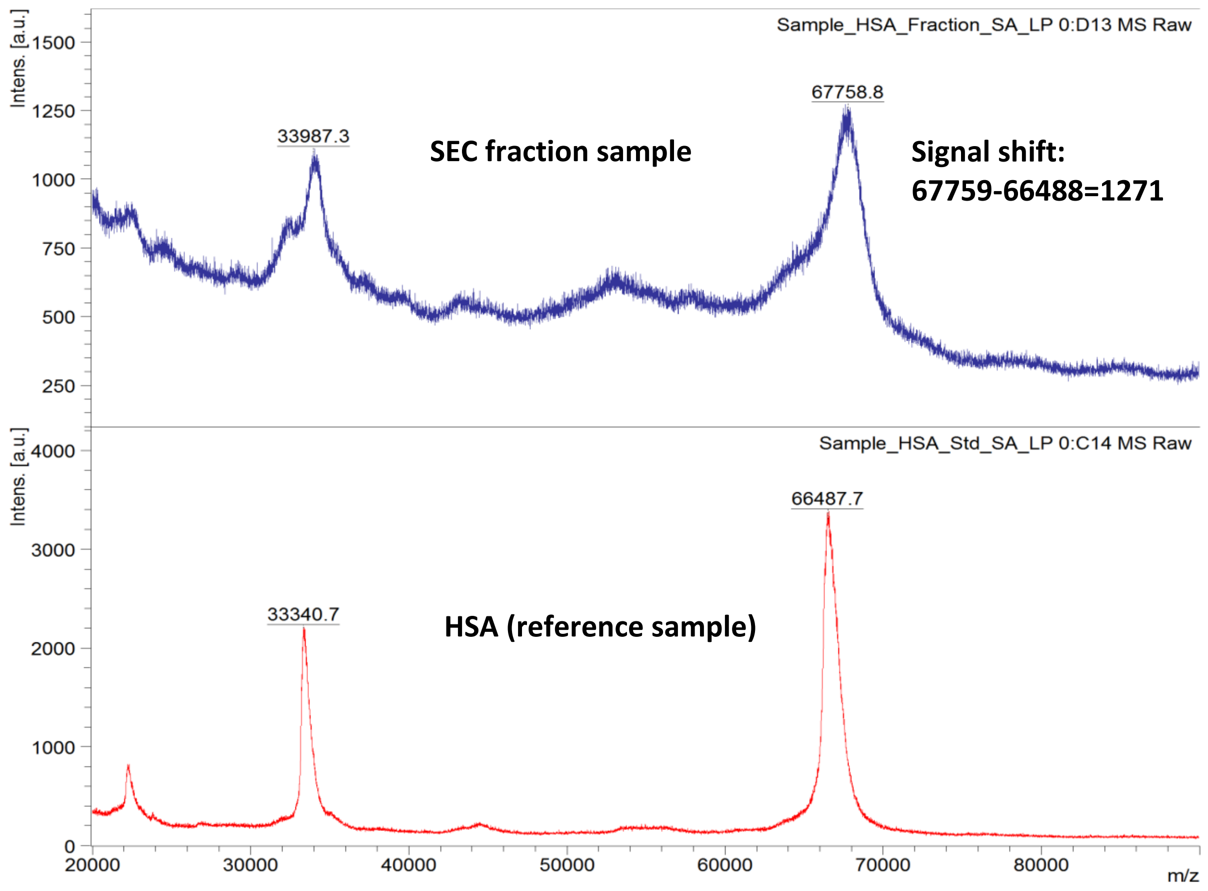
Sample_HSA_Fraction_SA_LP 0:D13 MS Raw
67758.8
33987.3
SEC fraction sample
Signal shift:
67759-66488=1271
Intens. [a.u.]
Sample_HSA_Std_SA_LP 0:C14 MS Raw
66487.7
33340.7
HSA (reference sample)
Intens. [a.u.]
m/z

4. RED-LIGHT-INDUCED CYSTEINYL-DESULFURIZATION

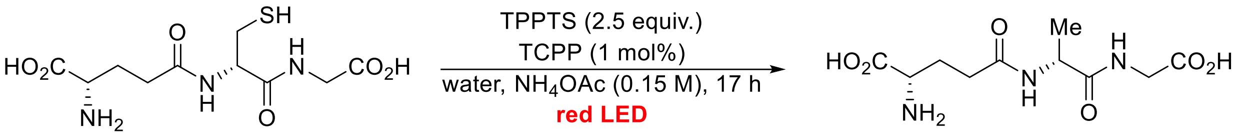

GSH **17**, 66%

Procedure: TCPP (1 mol%) was placed in the 10 ml glass vial containing a stirring bar and dissolved in the 0.15 M NH_4OAc solution and degassed (Ar flow, sonication) for ca. 15 min. To this solution glutathione (62 mg, 0.2 mmol, 1 equiv.) and sodium 3,3',3''-phosphanetriyltribenzenesulfonate (TPPTS, 335 mg, 85% purity, 0.5 mmol, 2.5 equiv.) were added, and the mixture was flushed with Ar. The sealed vial was then irradiated (660 nm, 100% power of the UOSlab Miniphoto photoreactor) for 17 h. After that time, the crude product **3** was isolated by preparative RP-HPLC (flow rate: 19 ml/min, elution: gradient 2/98-80/20 acetonitrile/water containing 0.02% trifluoracetic acid) and obtained as TFA salt (51.1 mg, **66%** yield).

^{1}H NMR (500 MHz, D_2O) δ 4.36 (q, J = 7.2 Hz, 1H), 3.87 – 3.69 (m, 3H), 2.51 (td, J = 7.4, 2.2 Hz, 2H), 2.16 (q, J = 7.2 Hz, 2H), 1.41 (d, J = 7.3 Hz, 3H) ppm.
^{13}C{H} NMR (126 MHz, D_2O) δ 187.1, 182.1, 180.4, 180.2, 168.6 (q, $^2J_{C\text{–}F}$ = 35.4 Hz, $CF_3\mathbf{C}O_2^-$), 122.0 (q, $^1J_{C\text{–}F}$ = 291.8 Hz, $\mathbf{C}F_3CO_2^-$), 59.7, 55.3, 48.9, 36.9, 31.8, 28.9, 22.3 ppm.
^{19}F NMR (470 MHz, D_2O): –75.6 ppm.

Analytical data for compound **17** (in TFA salt form) are in agreement with the literature data reported for free acid form of compound **17**.[11]

[11] Gao, X.-F.; Du, J.-J.; Liu, Z.; Guo, J. Visible-Light-Induced Specific Desulfurization of Cysteinyl Peptide and Glycopeptide in Aqueous Solution. *Org. Lett.* **2016**, *18*, 1166–1169.

5. NMR SPECTRA

Trifluoroacetate salt of (*R*)-4-((3-((2-amino-2-carboxyethyl)thio)propyl)amino)-4-oxobutanoic acid (3·TFA)

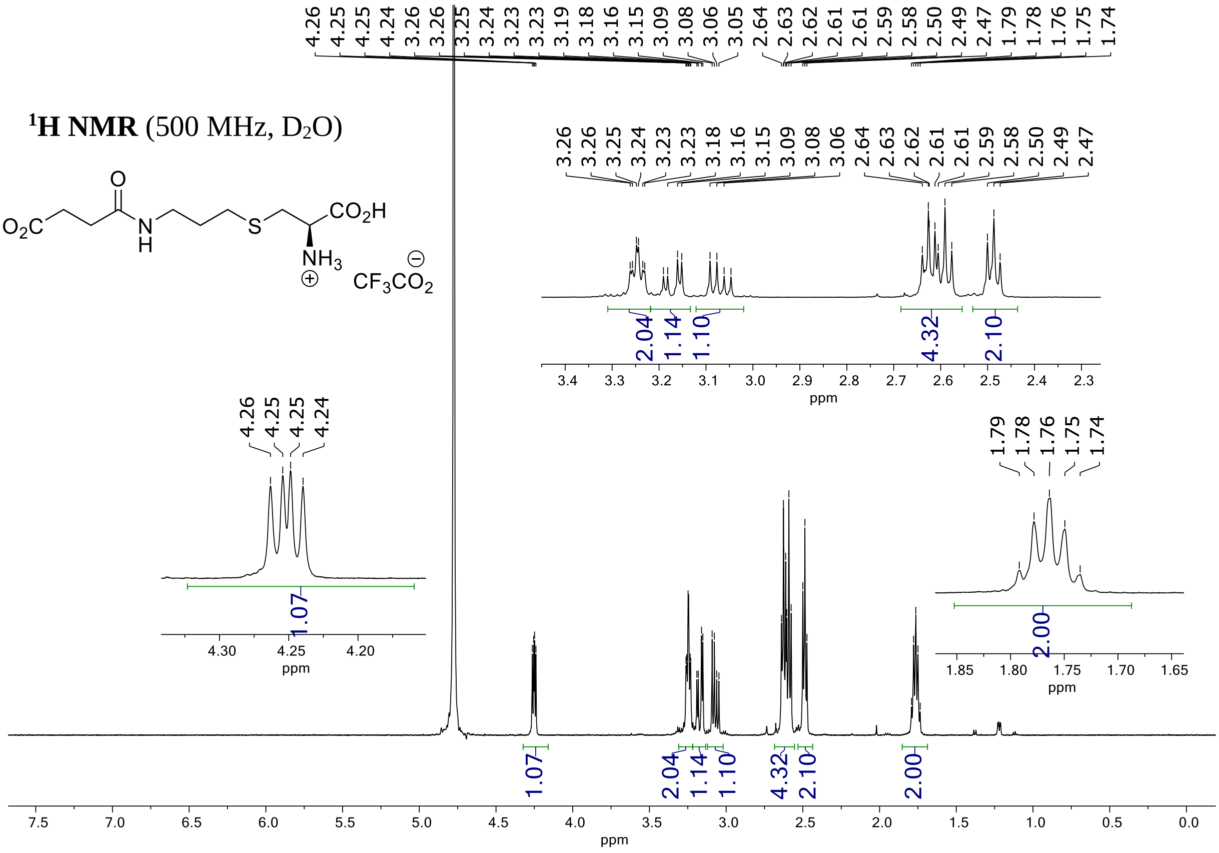

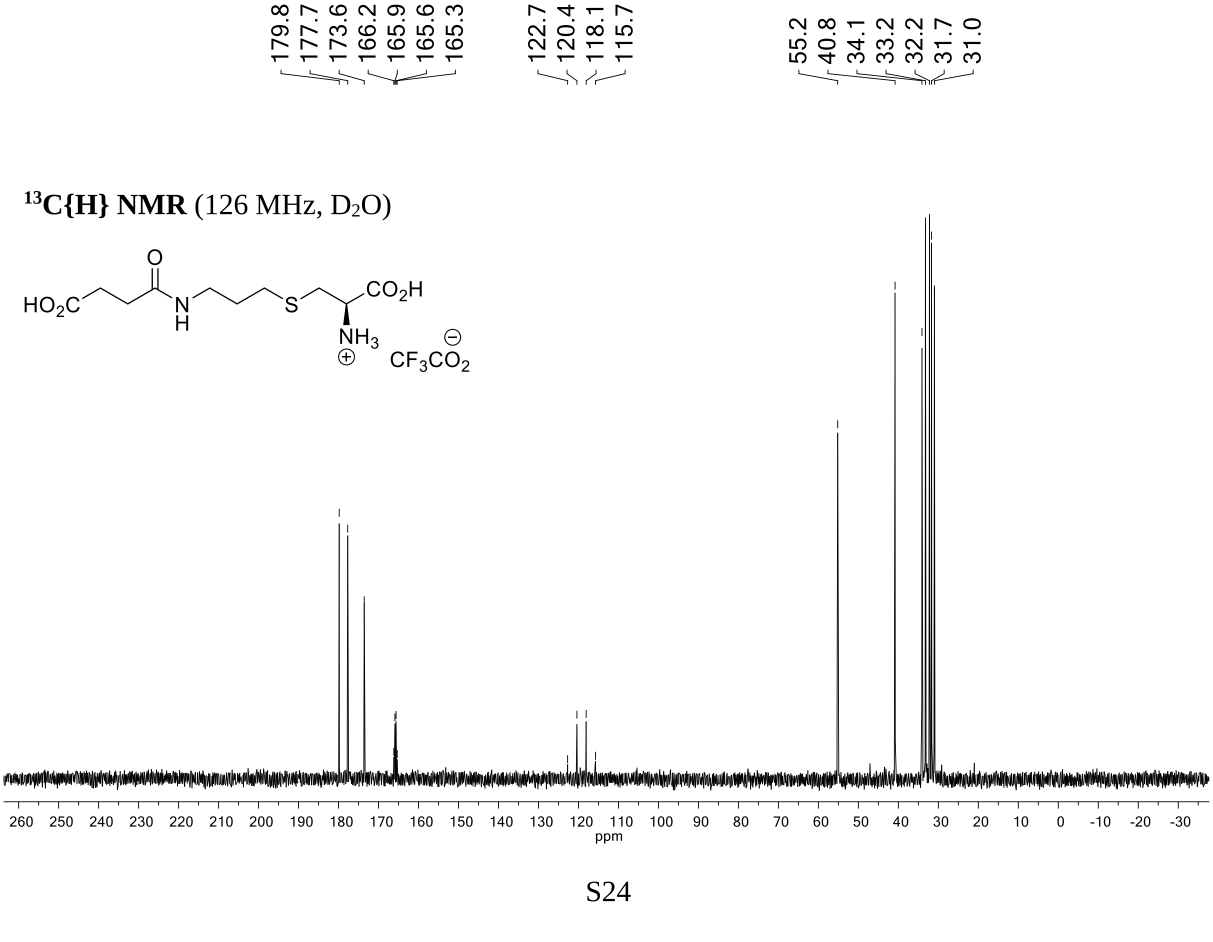

^{19}F NMR (470 MHz, D_2O)

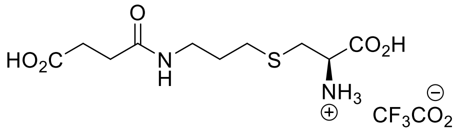

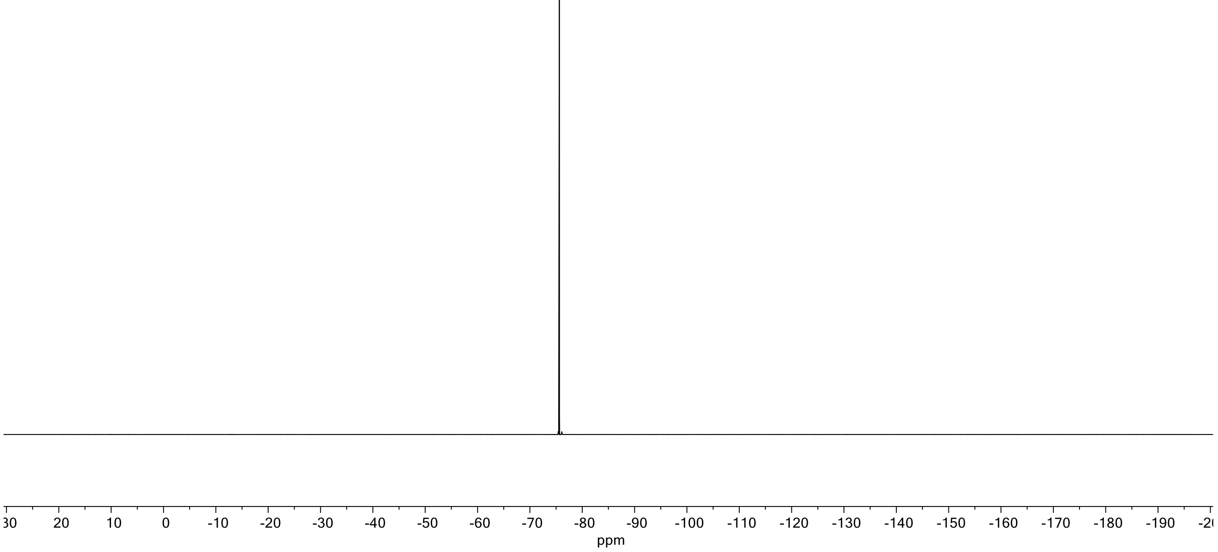

Trifluoroacetate salt of *S*-(3-hydroxy-3-methylbutyl)-L-cysteine (4·TFA)

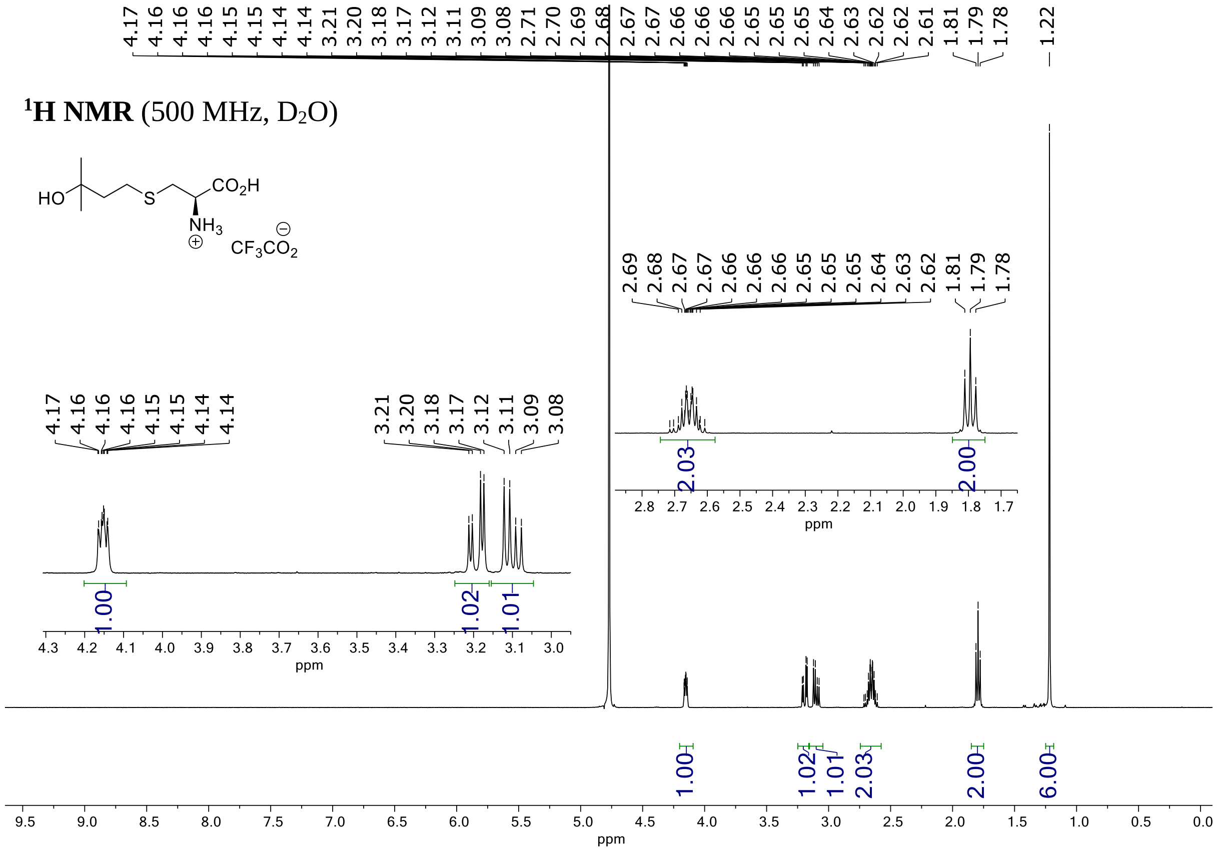

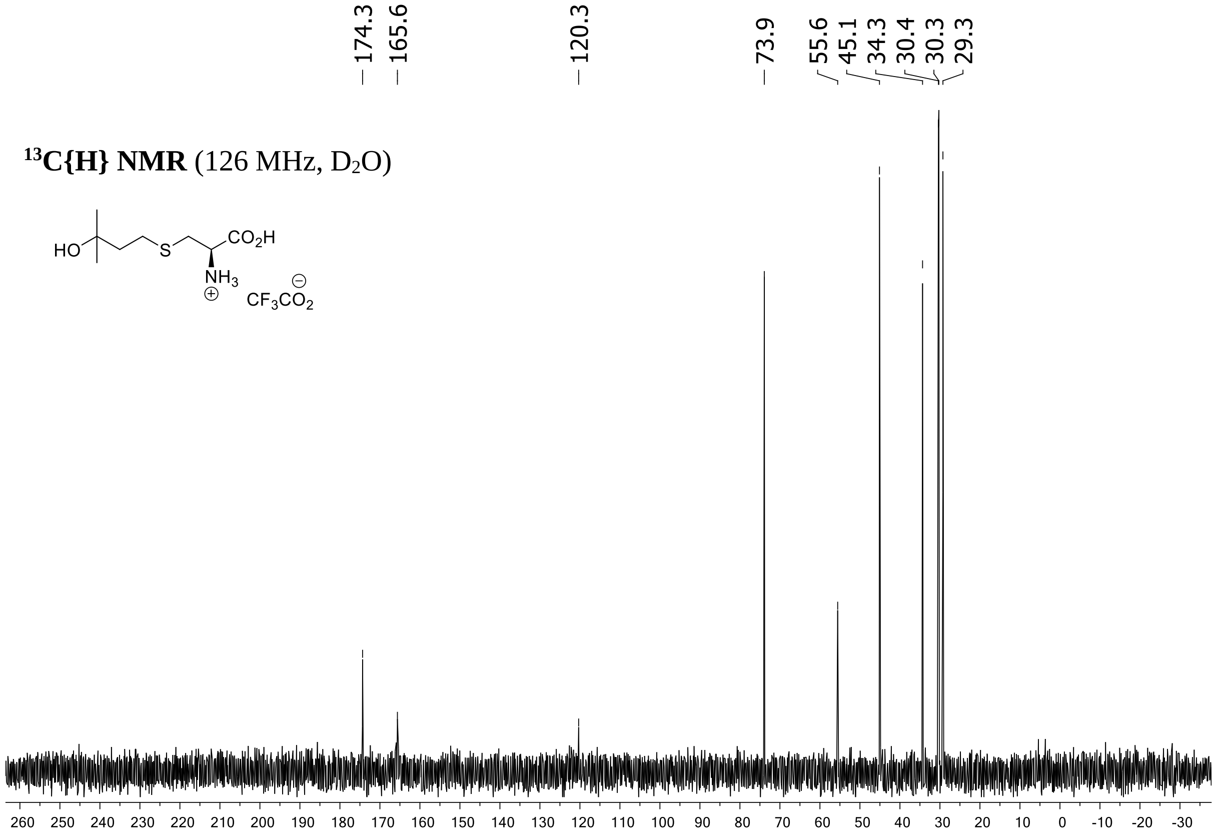

Trifluoroacetate salt of (*R*)-2-amino-21-oxo-25-((3a*S*,4*S*,6a*R*)-2-oxohexahydro-1*H*-thieno[3,4-d]imidazol-4-yl)-8,11,14,17,20-pentaoxa-4-thiapentacosanoic acid (5·TFA)

^{1}H NMR (500 MHz, D_2O)

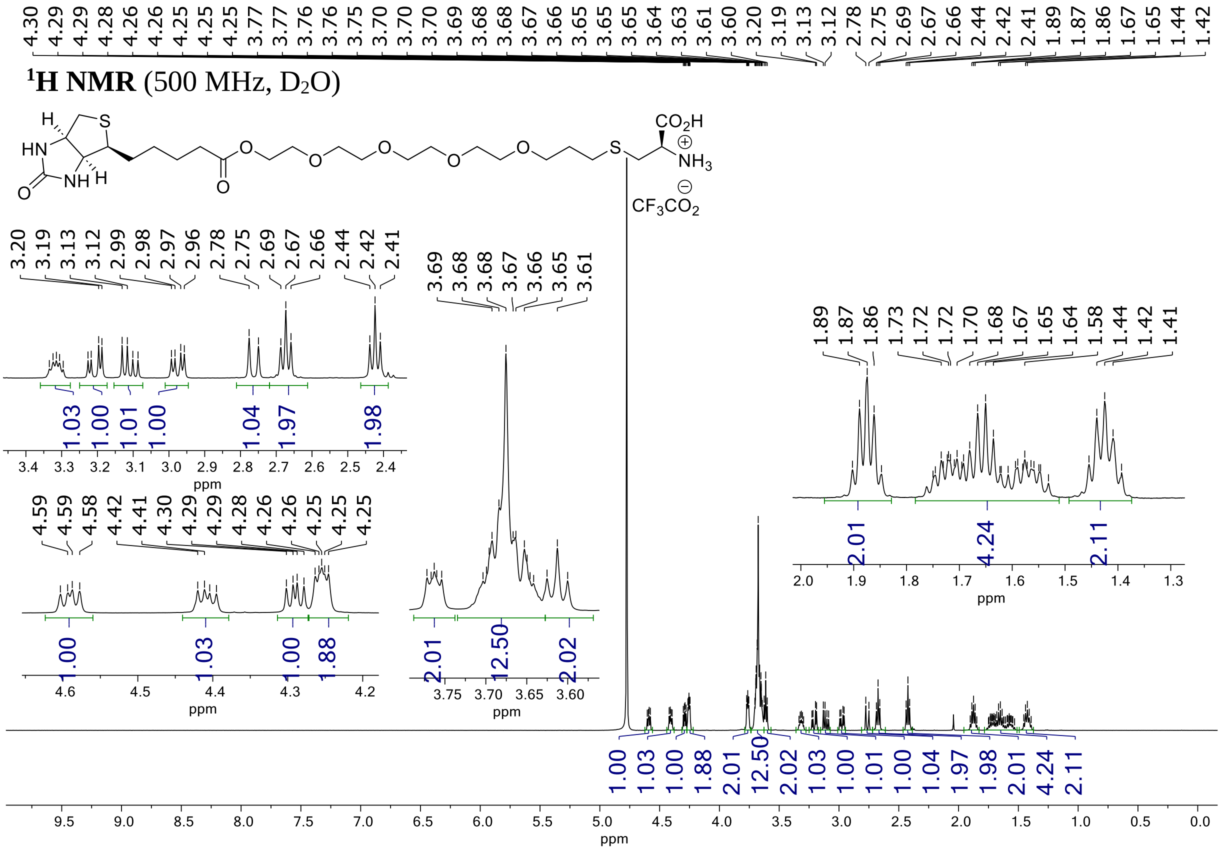

^{13}C{H} NMR (126 MHz, D_2O)

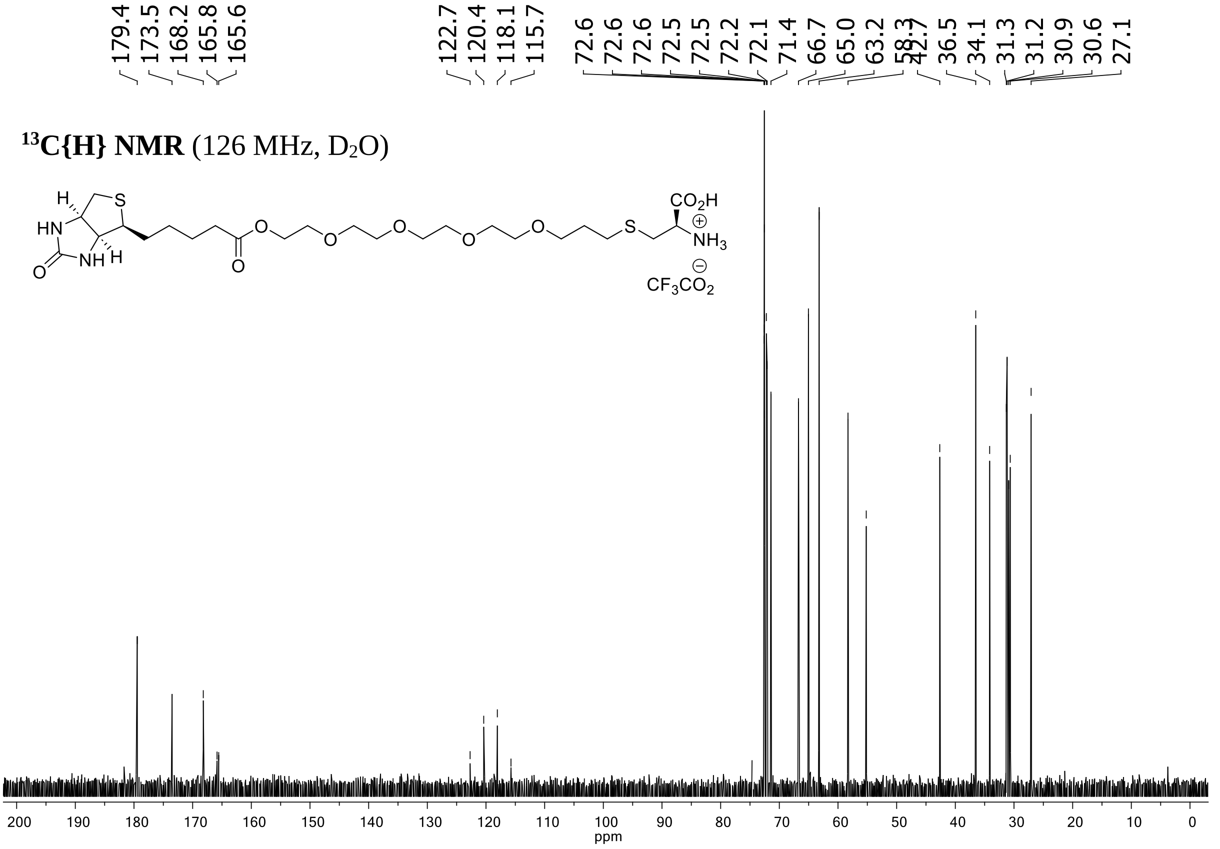

^{19}F NMR (470 MHz, D_2O)

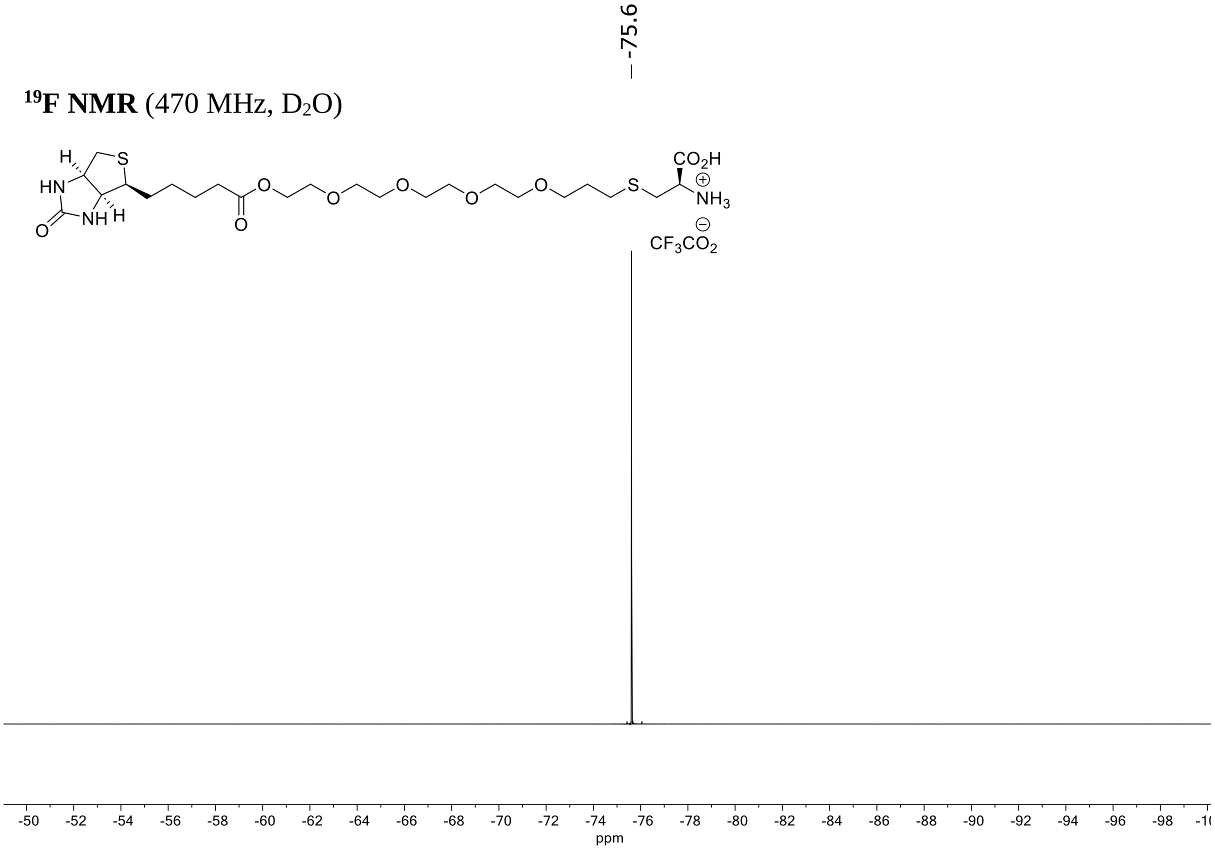

Trifluoroacetate salt of *N*5-((*R*)-1-((carboxymethyl)amino)-3-((3-hydroxy-3-methylbutyl)thio)-1-oxopropan-2-yl)-L-glutamine (2·TFA)

^{1}H NMR (500 MHz, D_2O)

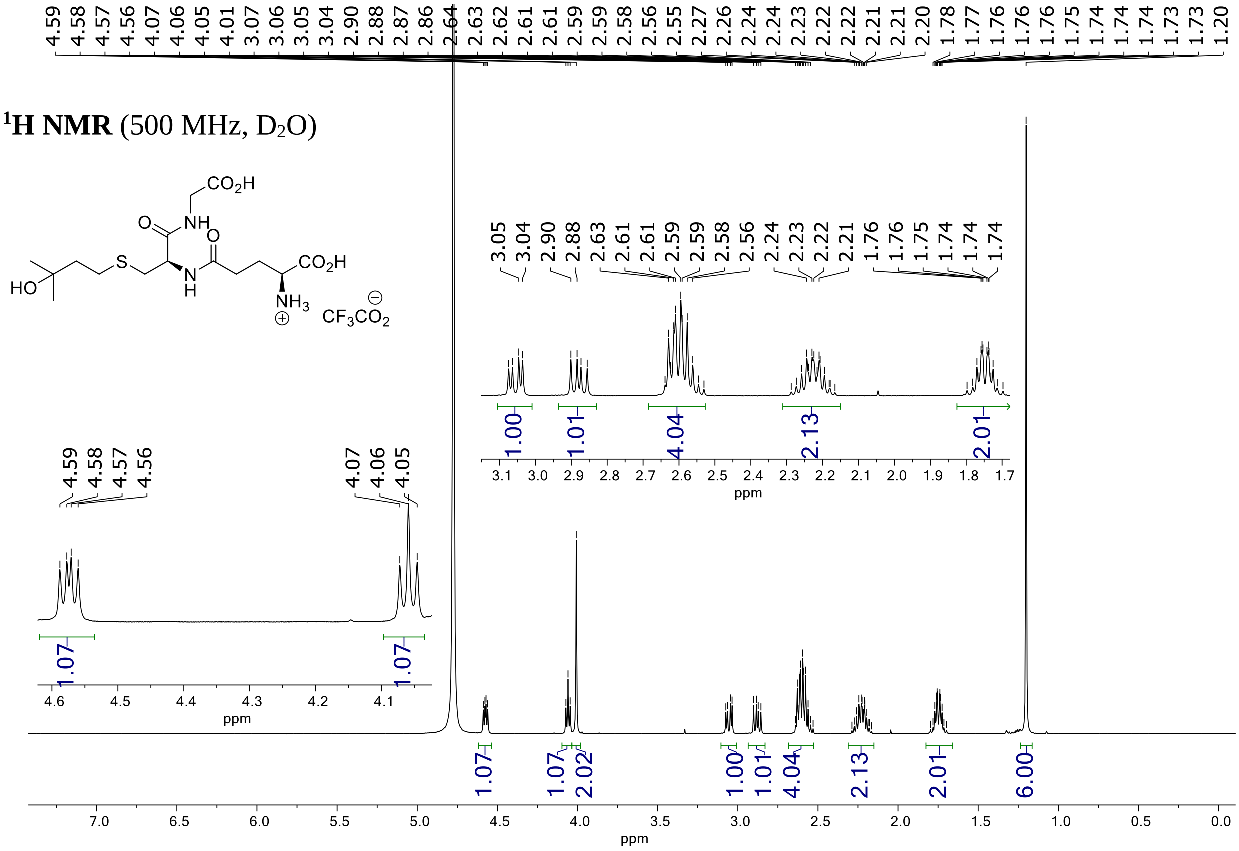

^{13}C{H} NMR (126 MHz, D_2O)

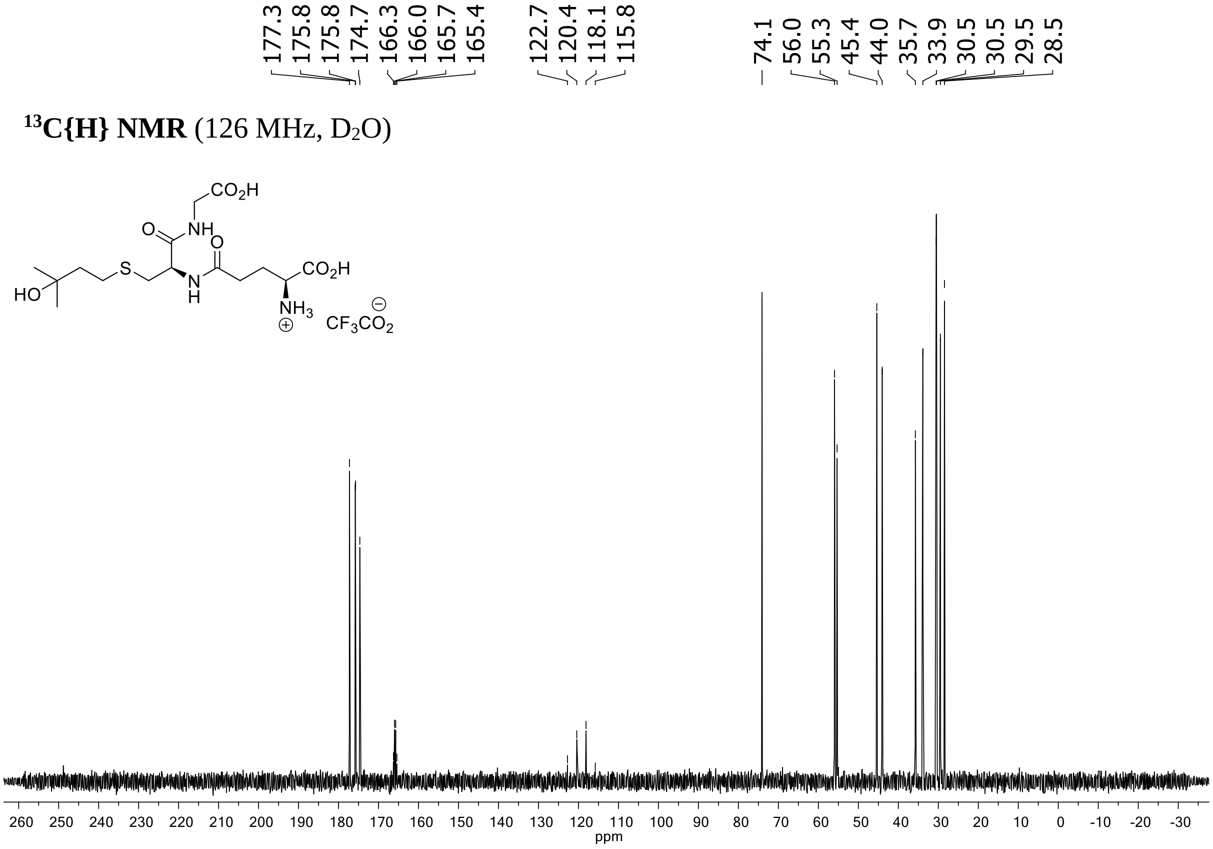

^{19}F NMR (470 MHz, D_2O)

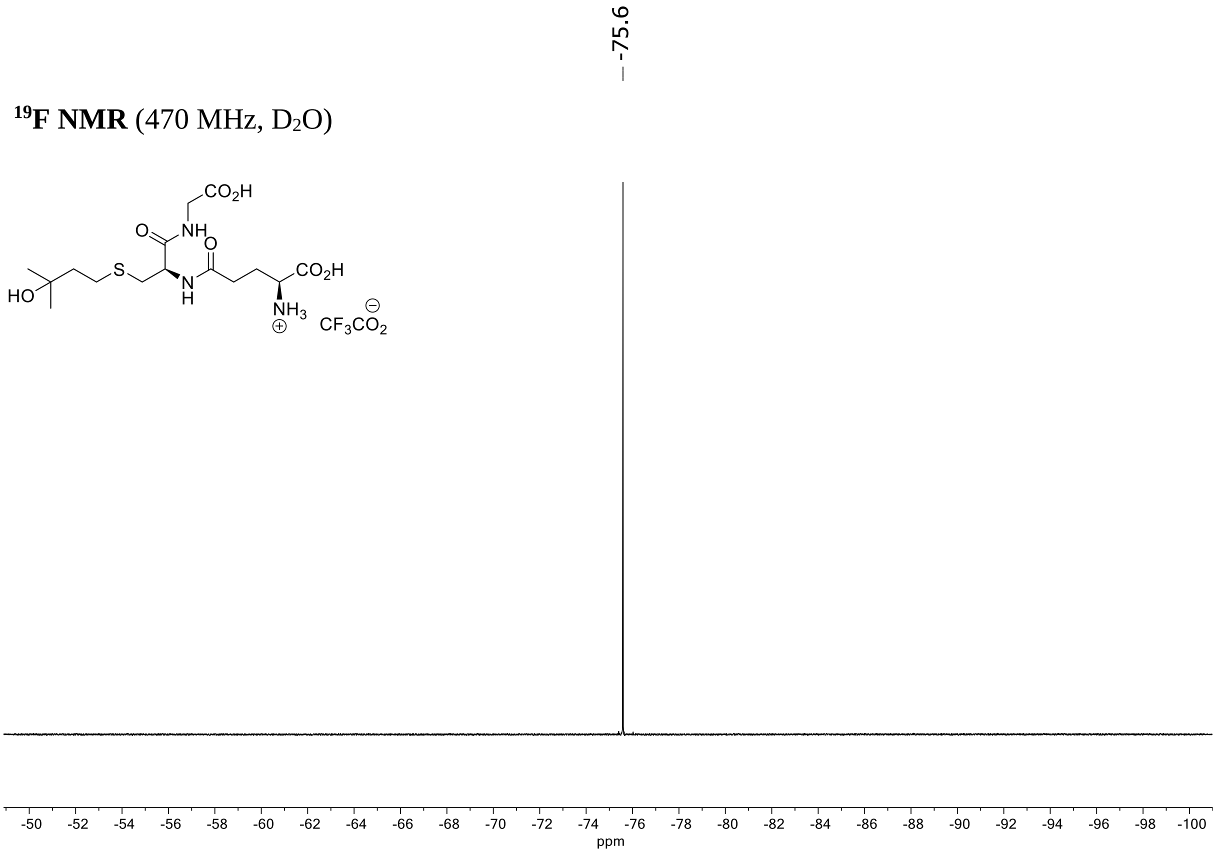

Trifluoroacetate salt of N5-((R)-3-((4-carboxybutyl)thio)-1-((carboxymethyl)amino)-1-oxopropan-2-yl)-L-glutamine (6·TFA)

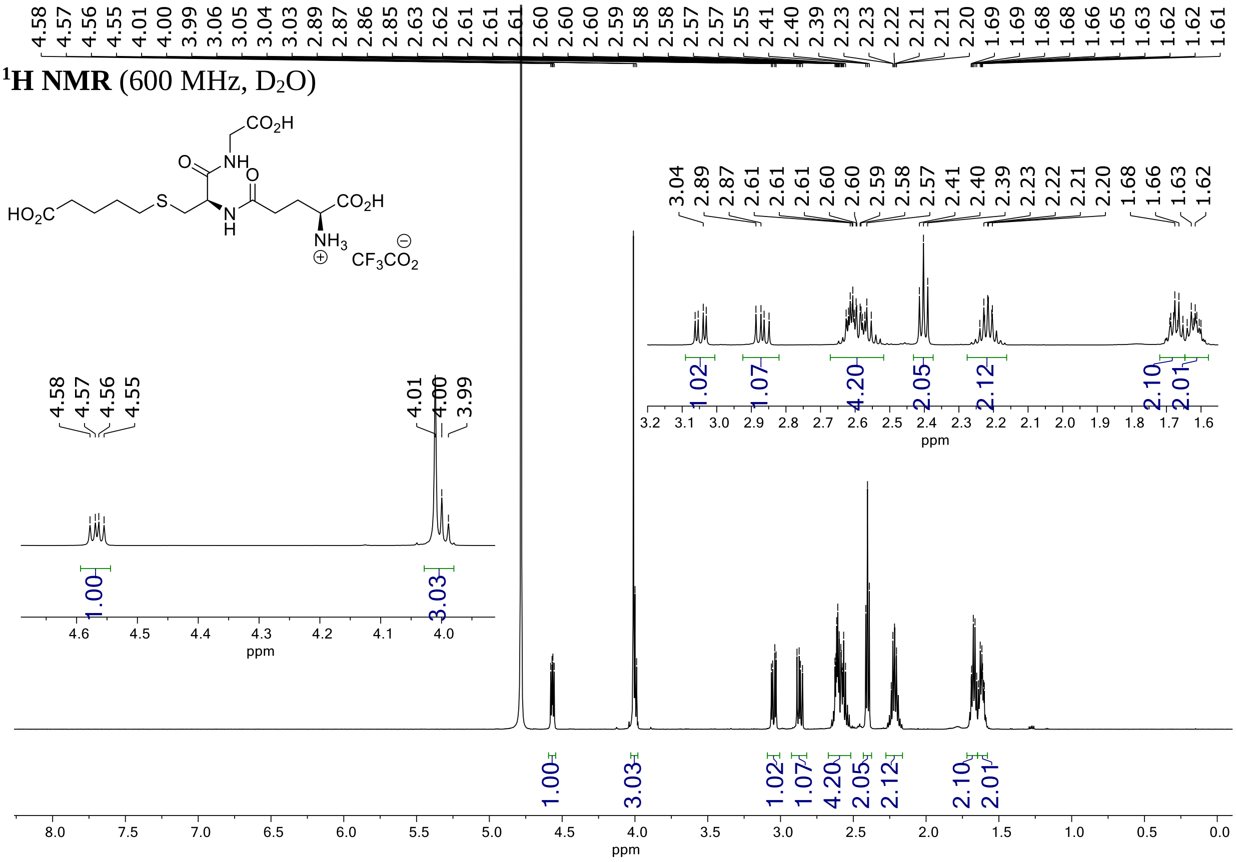

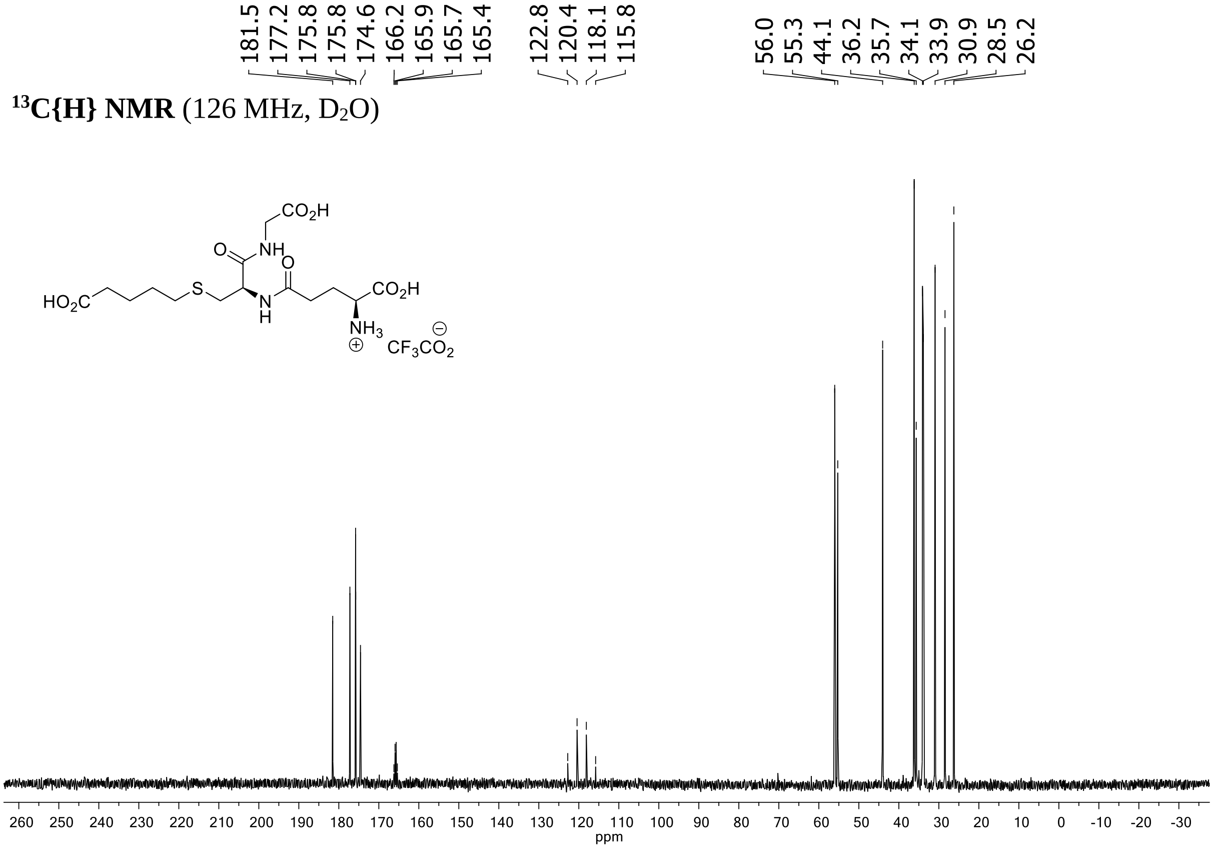

Trifluoroacetate salt of *N*5-((*R*)-1-((carboxymethyl)amino)-3-((3-(3-carboxypropanamido)propyl)thio)-1-oxopropan-2-yl)-L-glutamine (7·TFA)

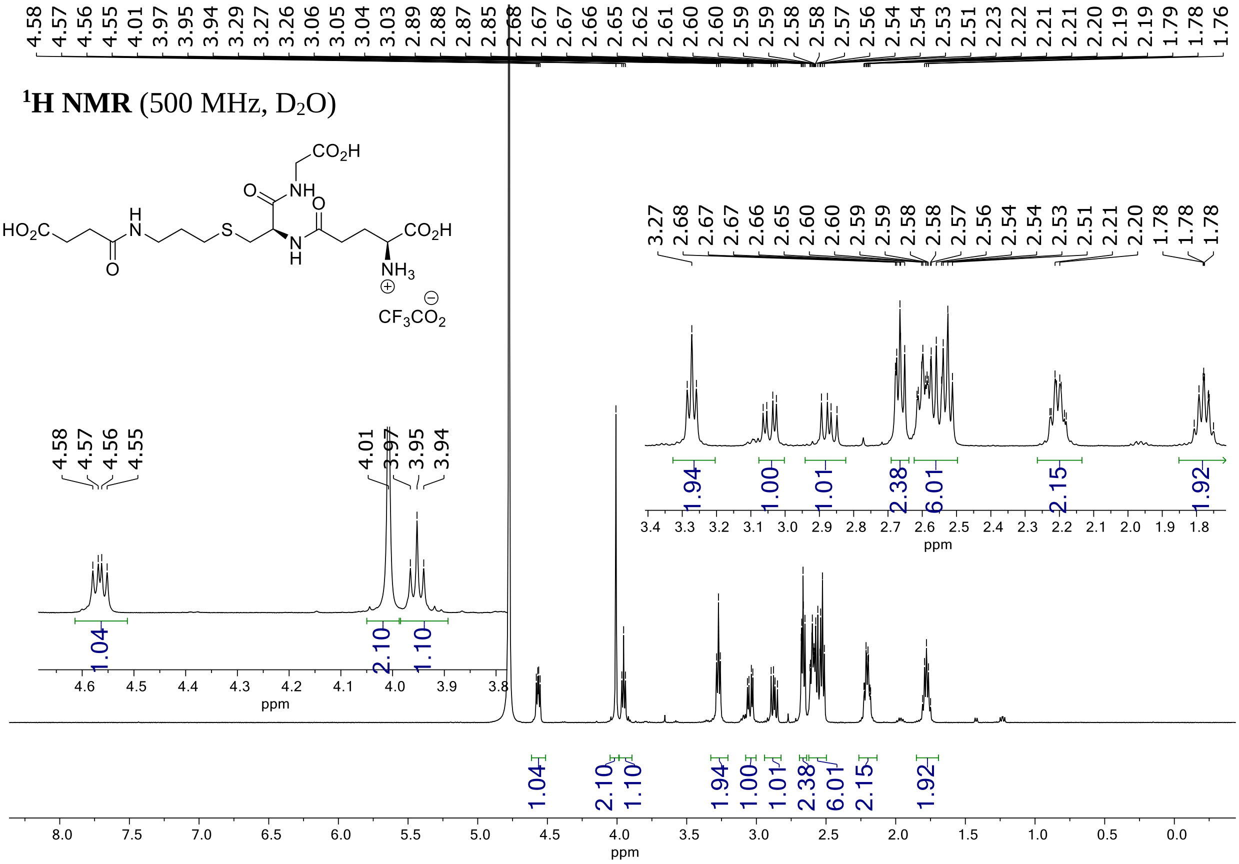

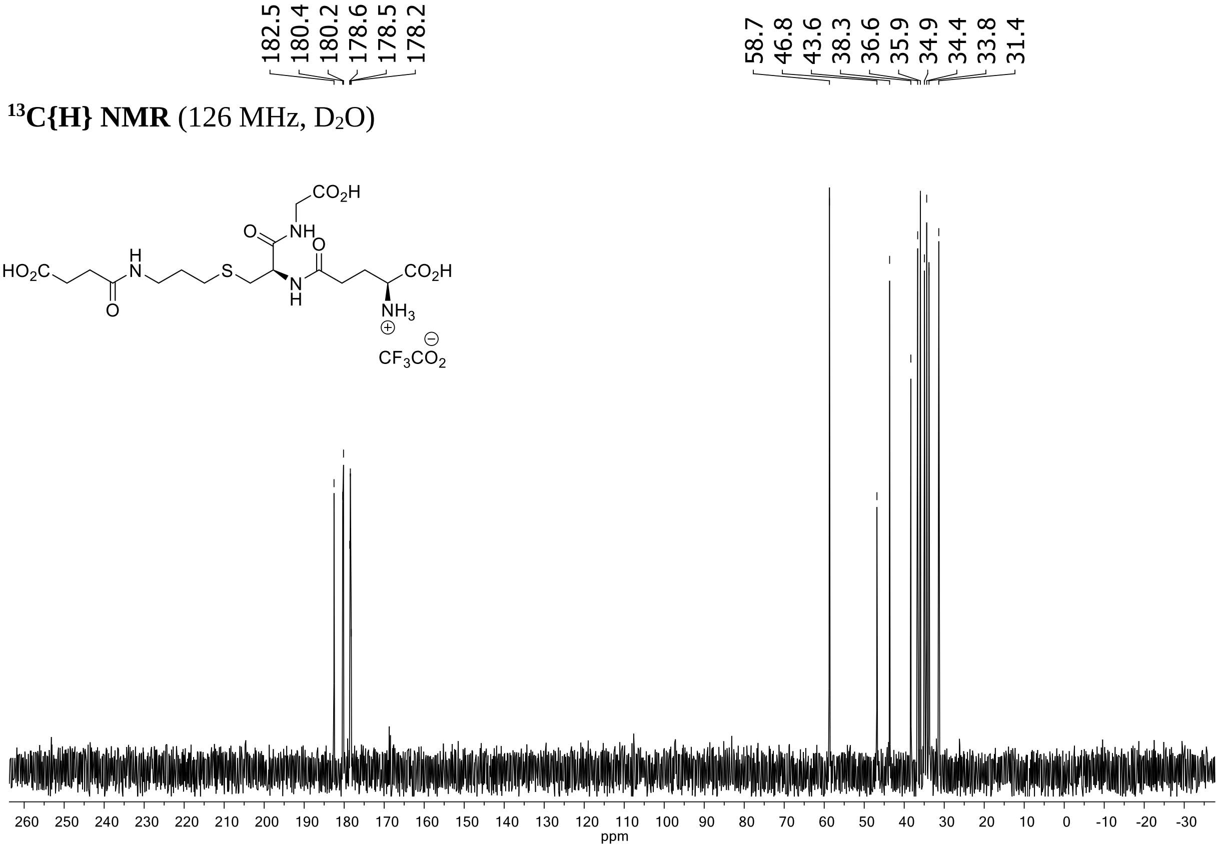

^{19}F NMR (470 MHz, D_2O)

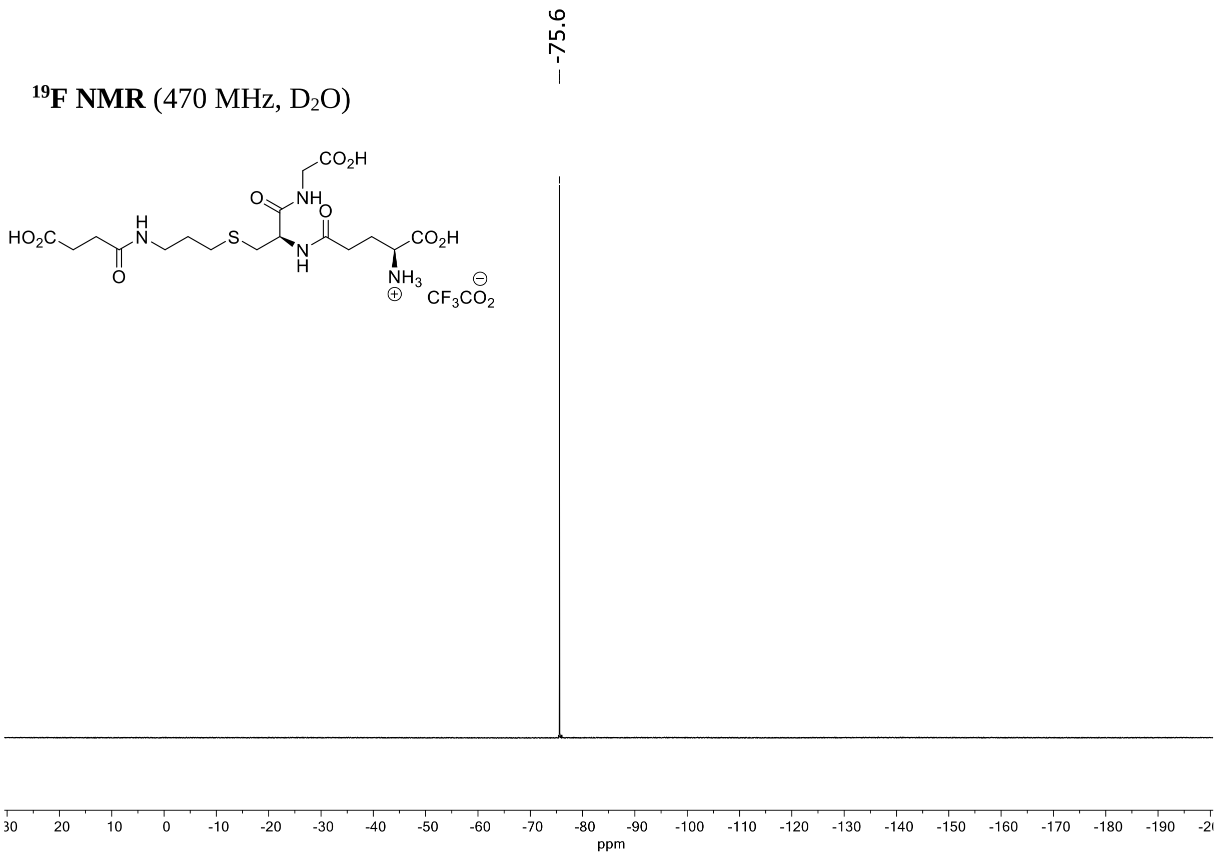

Trifluoroacetate salt of (3*S*,12*R*,17*S*)-17-amino-12-((carboxymethyl)carbamoyl)-5,14-dioxo-6-oxa-10-thia-4,13-diazaheptadecane-1,3,17-tricarboxylic acid (8·TFA)

^{1}H NMR (500 MHz, D_2O)

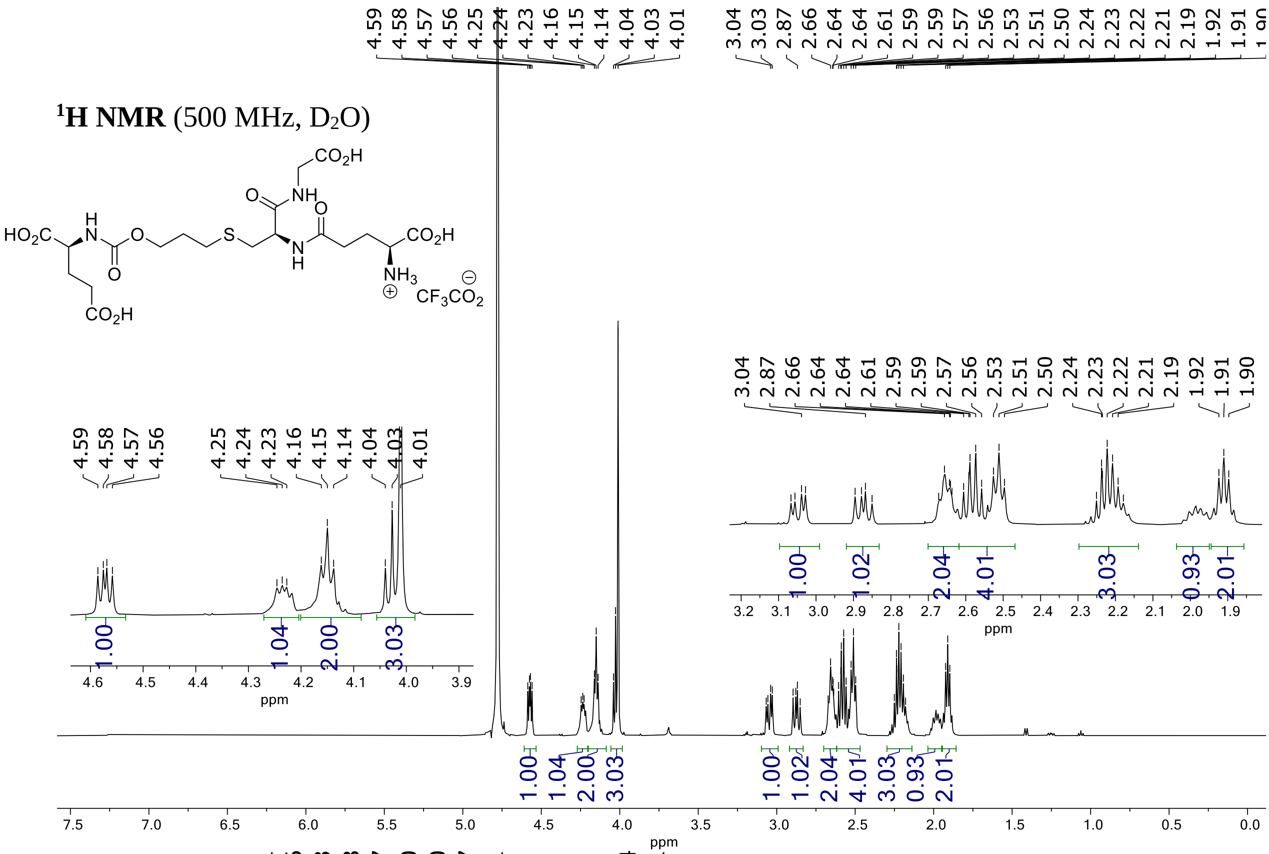

^{13}C{H} NMR (126 MHz, D_2O)

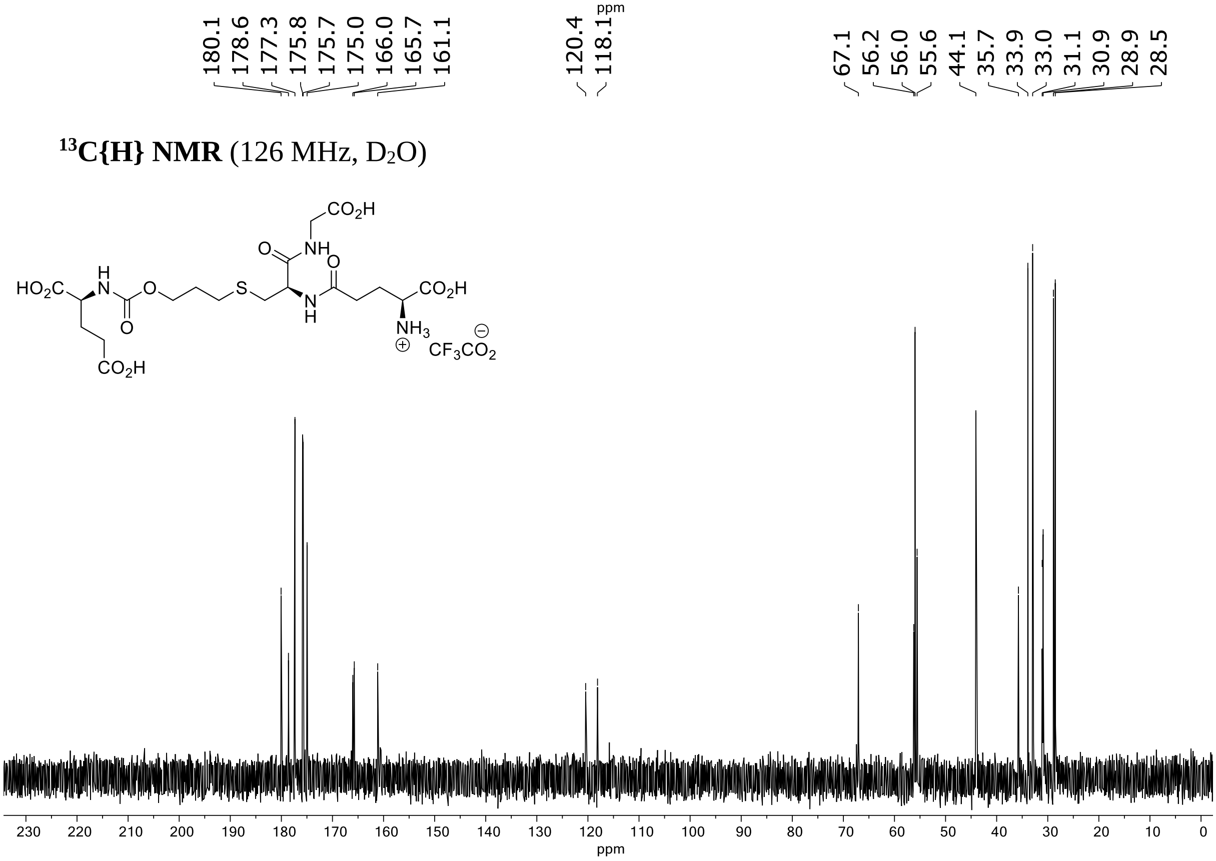

Trifluoroacetate salt of (2*S*,11*R*,16*S*)-16-amino-11-((carboxymethyl)carbamoyl)-2-(hydroxymethyl)-4,13-dioxo-5-oxa-9-thia-3,12-diazaheptadecanedioic acid (9·TFA)

^{1}H NMR (500 MHz, D_2O)

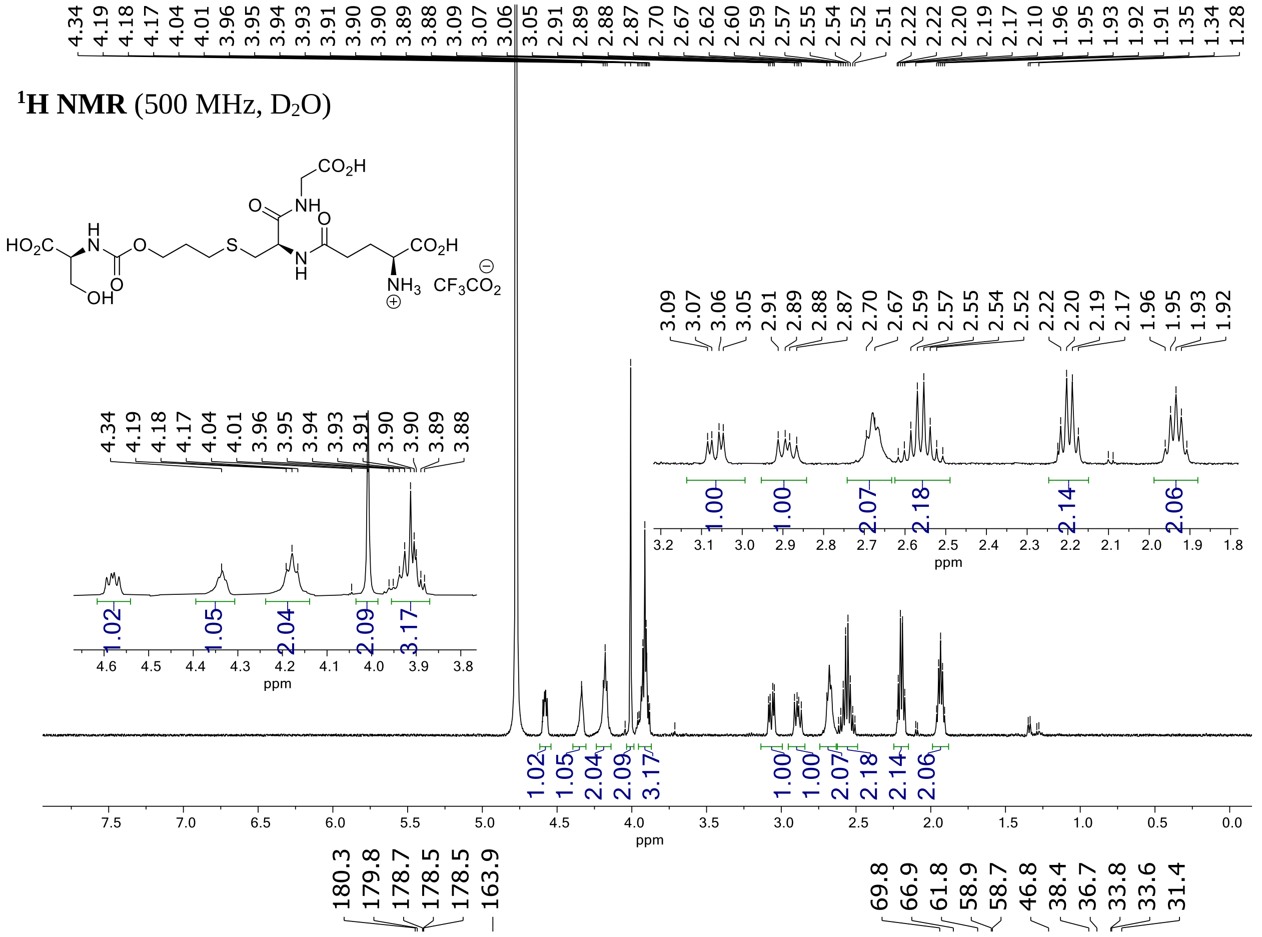

^{13}C{H} NMR (126 MHz, D_2O)

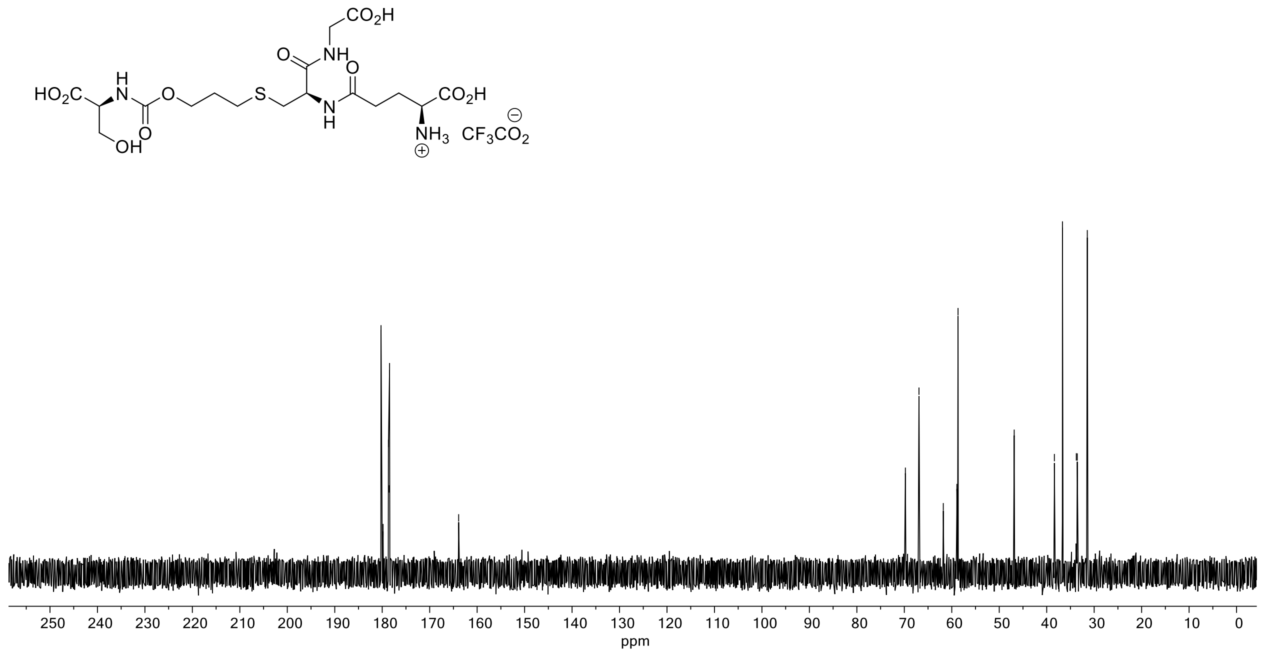

Trifluoroacetate salt of (18*R*,23*S*)-23-amino-18-((carboxymethyl)carbamoyl)-1-hydroxy-20-oxo-3,6,9,12-tetraoxa-16-thia-19-azatetracosan-24-oic acid (10·TFA)

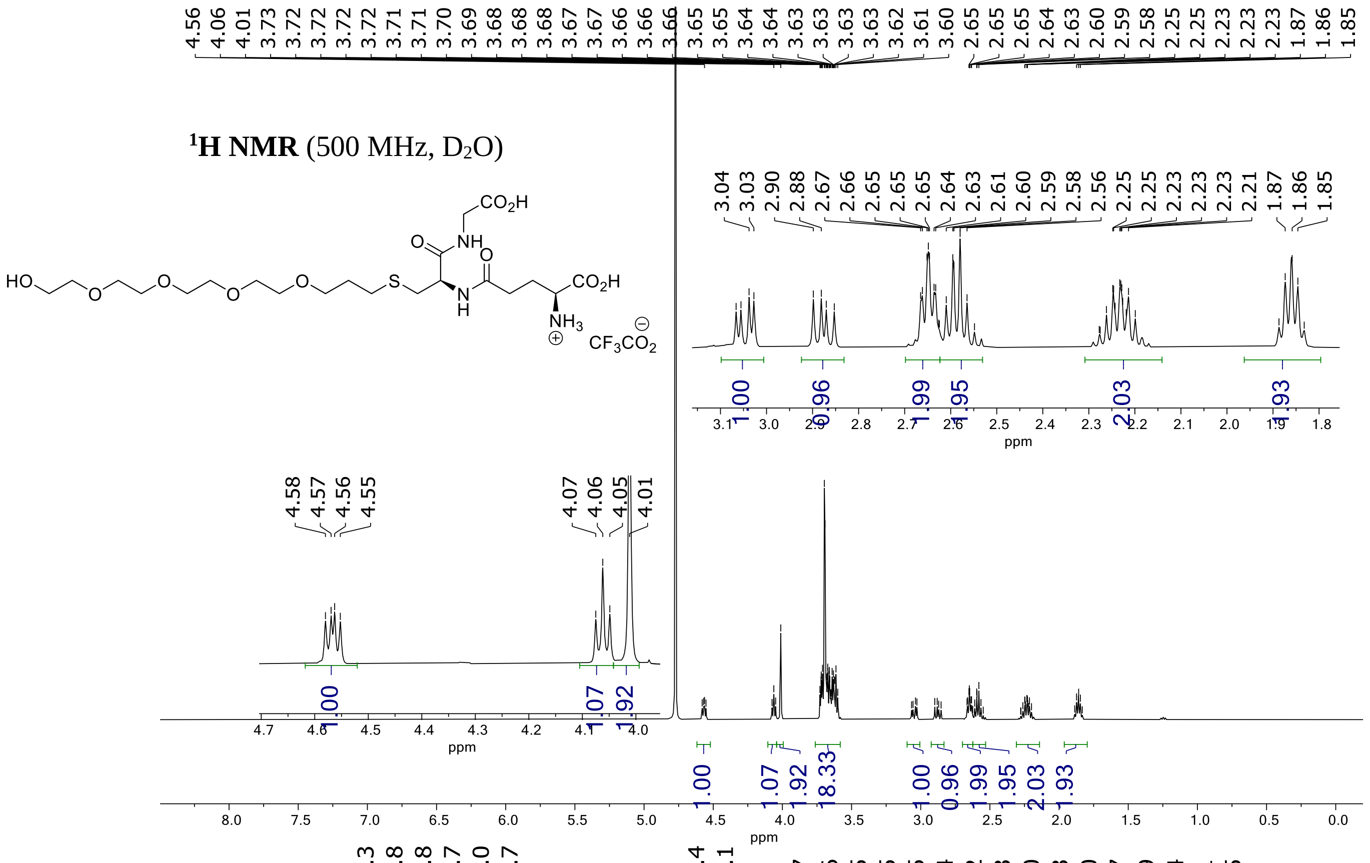

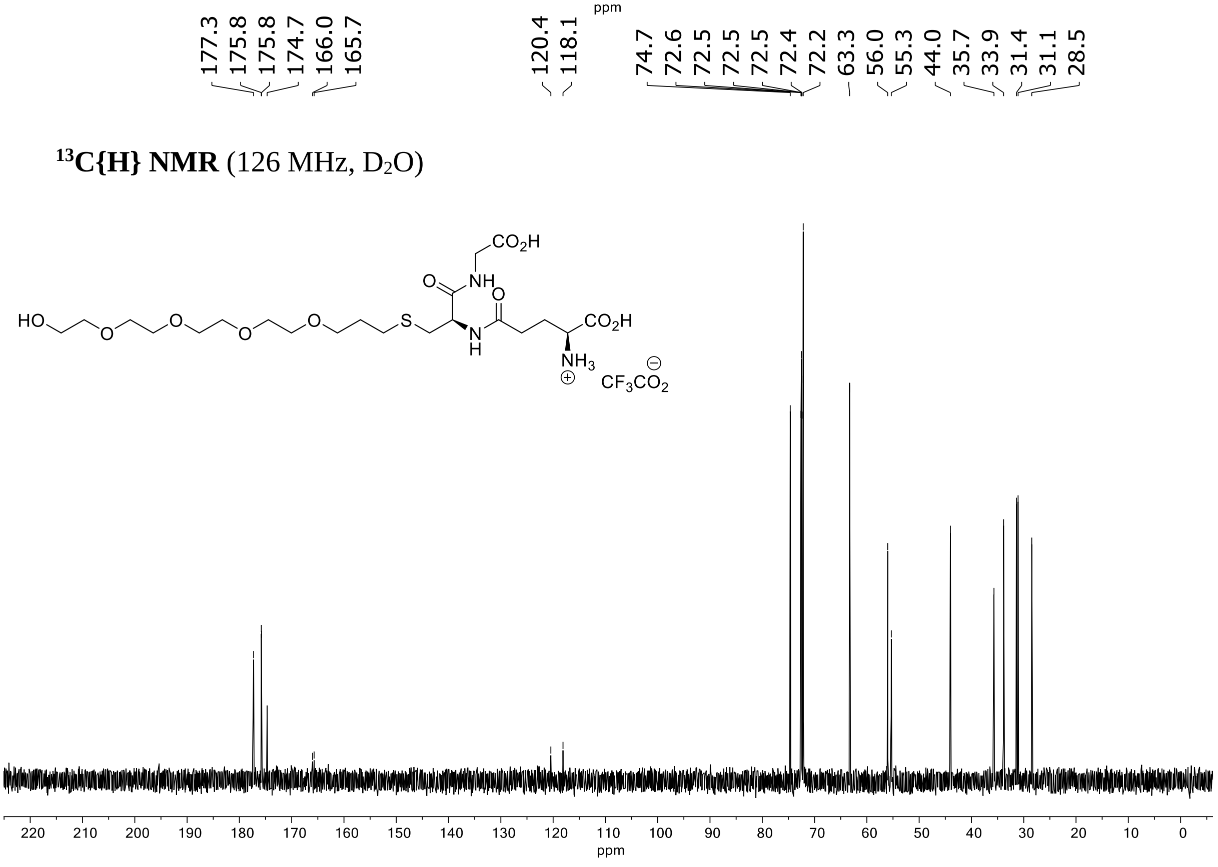

Trifluoroacetate salt of *N*5-((*R*)-1-((carboxymethyl)amino)-1-oxo-3-((3-((5-((3a*S*,4*S*,6a*R*)-2-oxohexahydro-1*H*-thieno[3,4-d]imidazol-4-yl)pentanoyl)oxy)propyl)thio)propan-2-yl)-L-glutamine (11·TFA)

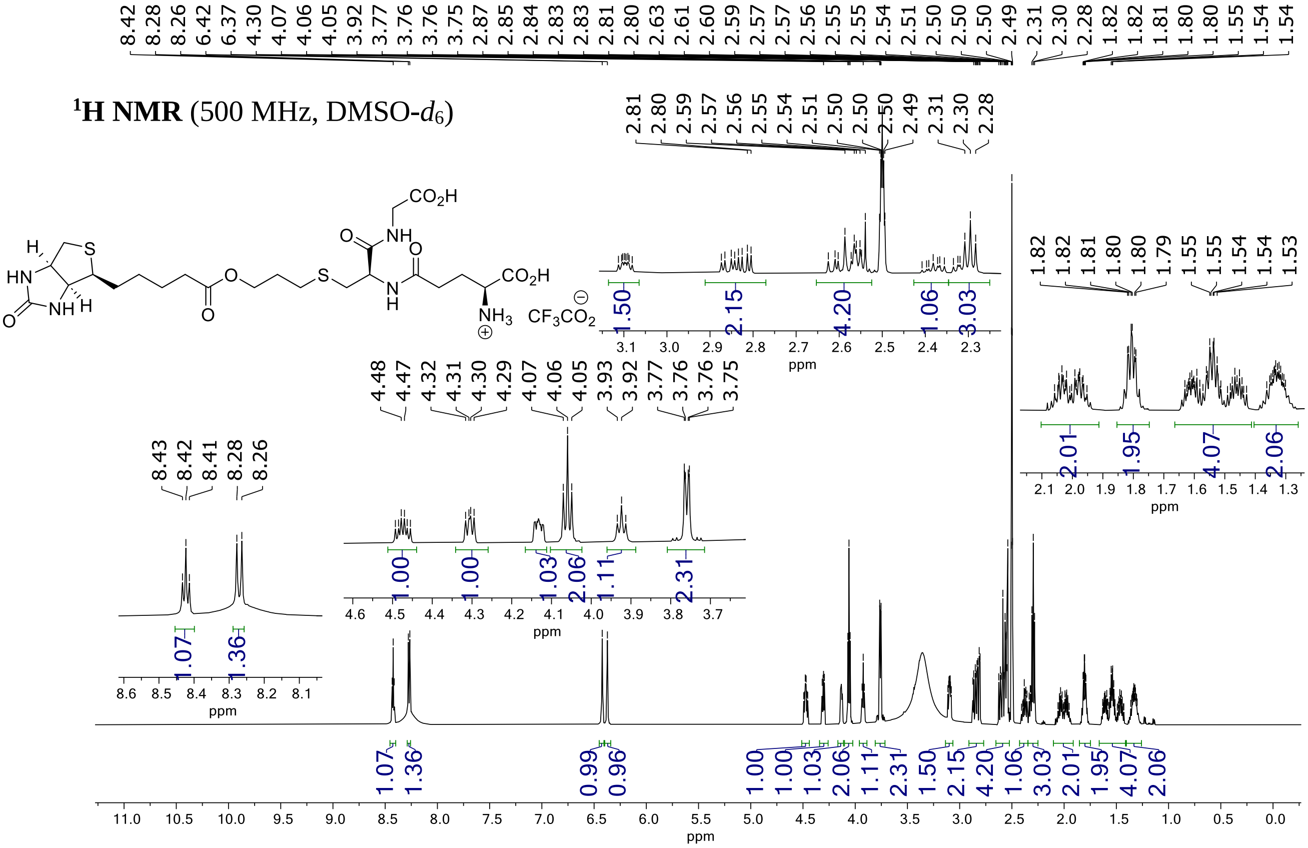

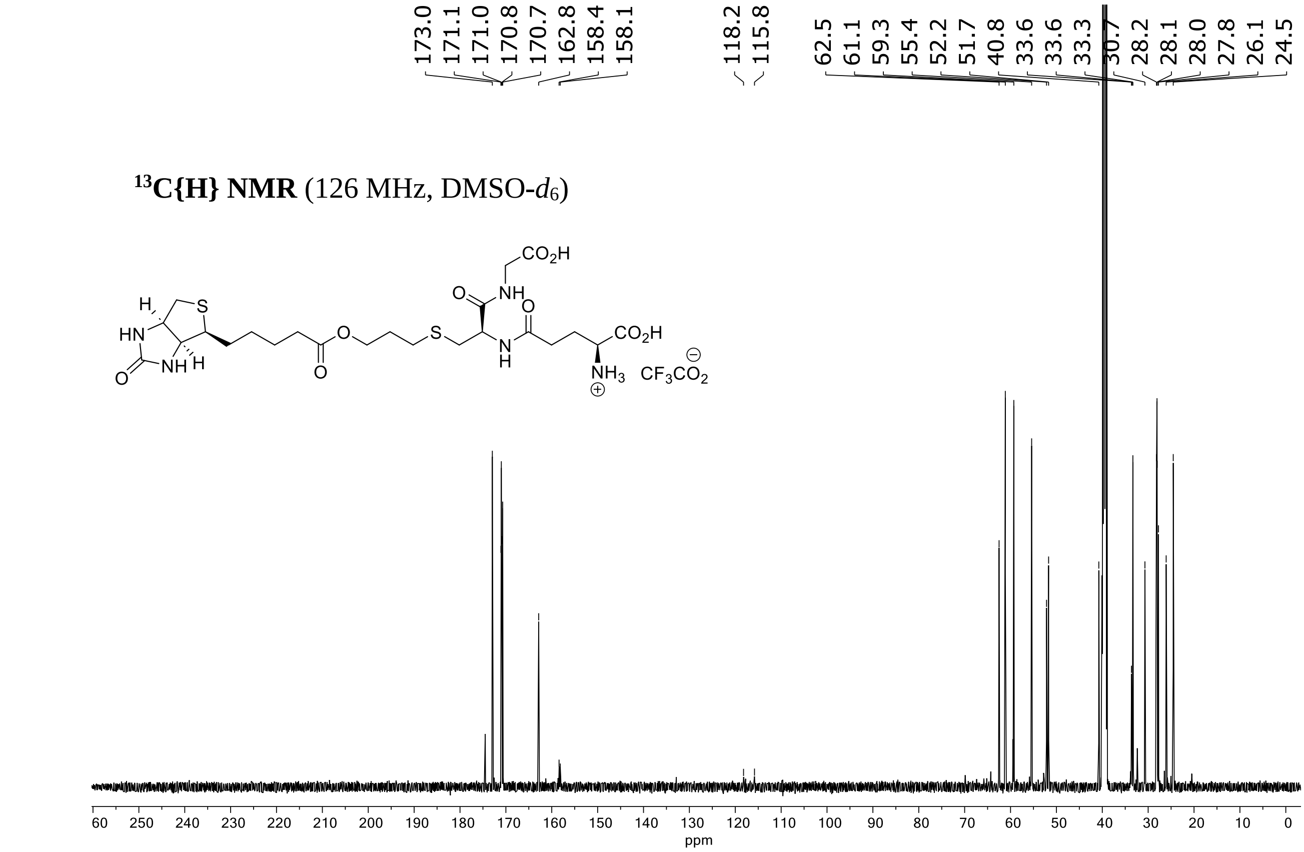

Trifluoroacetate salt of (24*R*,29*S*)-29-amino-24-((carboxymethyl)carbamoyl)-5,26-dioxo-1-((3a*S*,4*S*,6a*R*)-2-oxohexahydro-1*H*-thieno[3,4-d]imidazol-4-yl)-6,9,12,15,18-pentaoxa-22-thia-25-azatriacontan-30-oic acid (12·TFA)

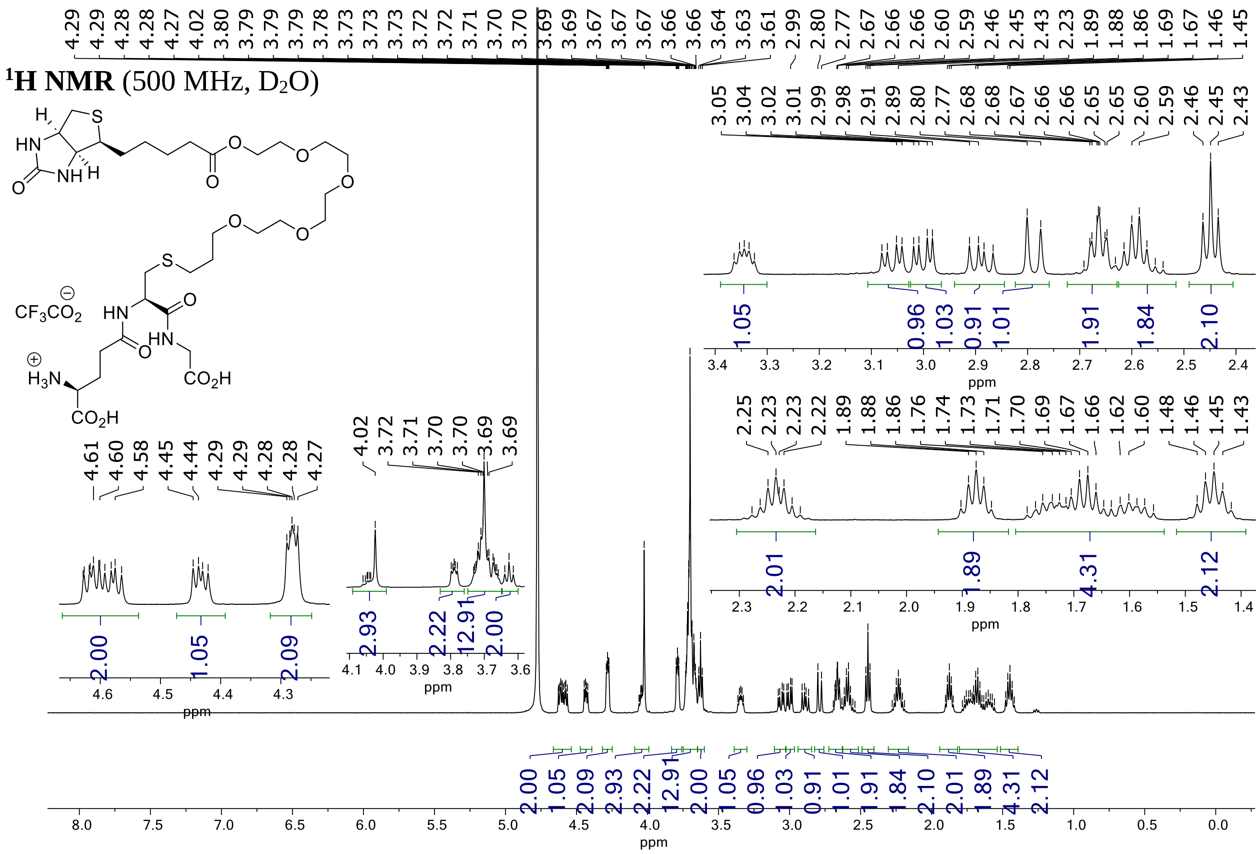

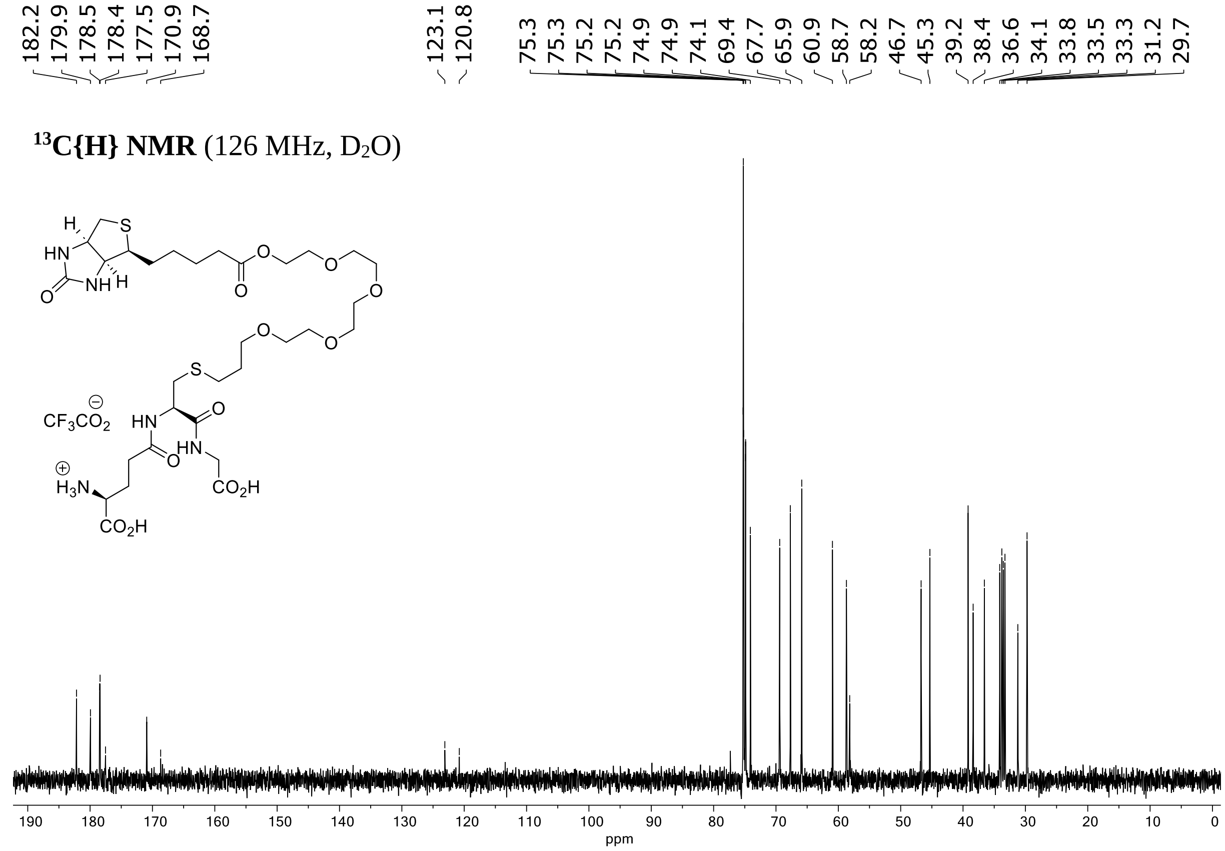

^{19}F NMR (470 MHz, D_2O)

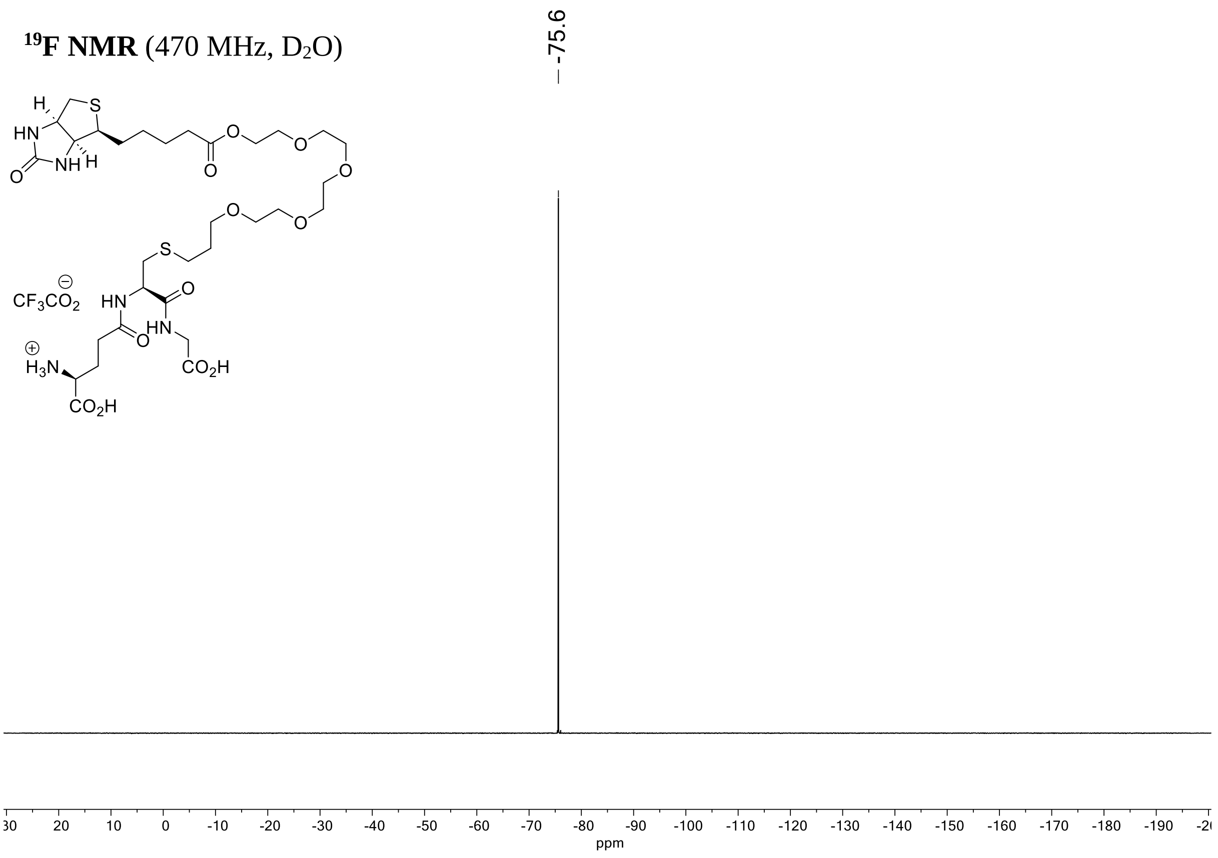

Trifluoroacetate salt of S-(3-(3-carboxypropanamido)propyl)-L-homocysteine (13·TFA)

^{1}H NMR (500 MHz, D_2O)

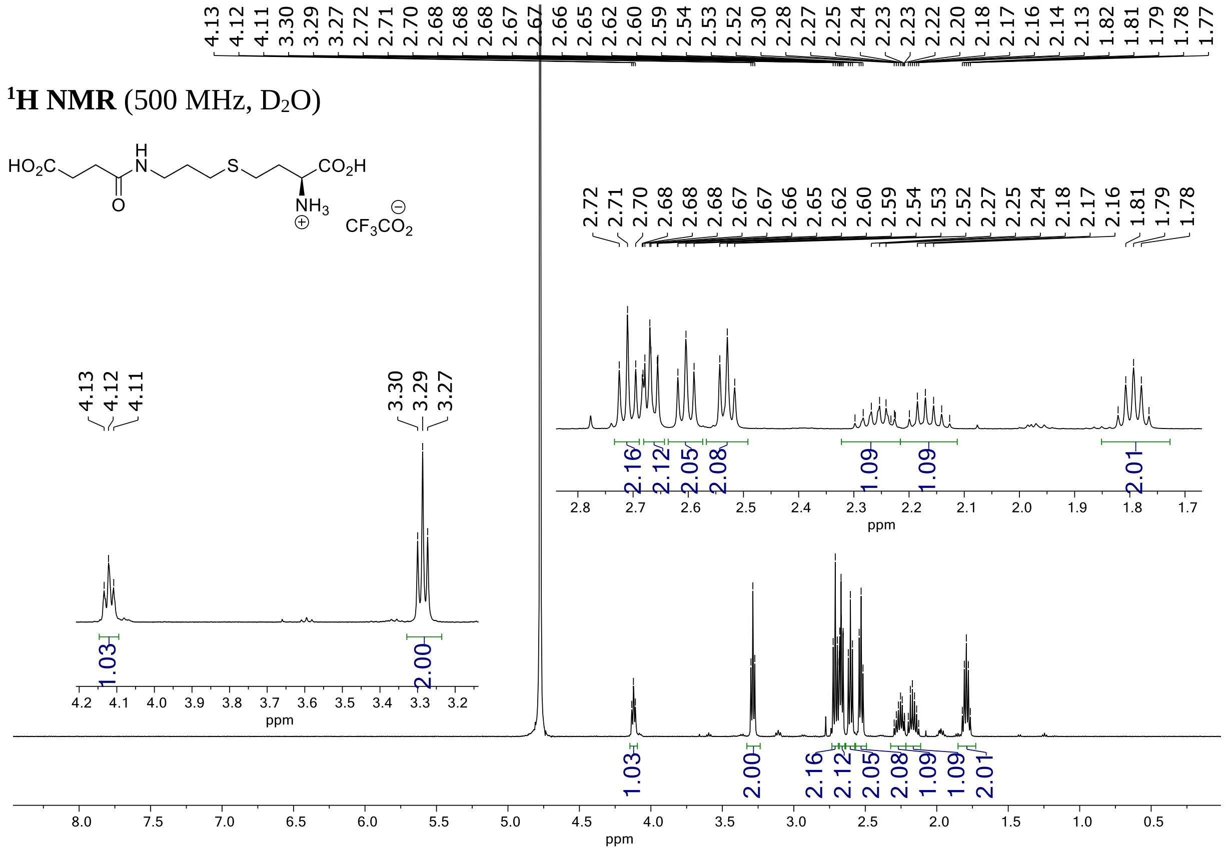

^{13}C{H} NMR (126 MHz, D_2O)

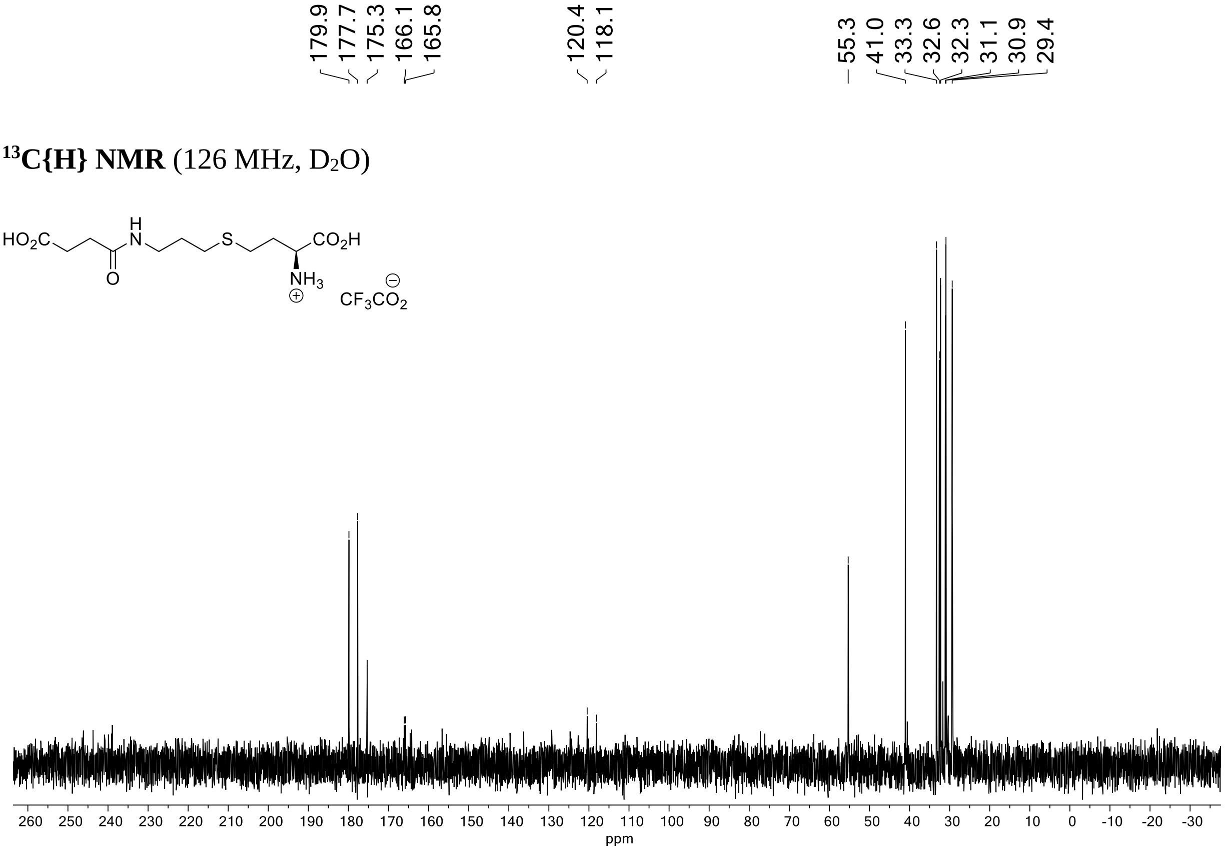

^{19}F NMR (470 MHz, D_2O)

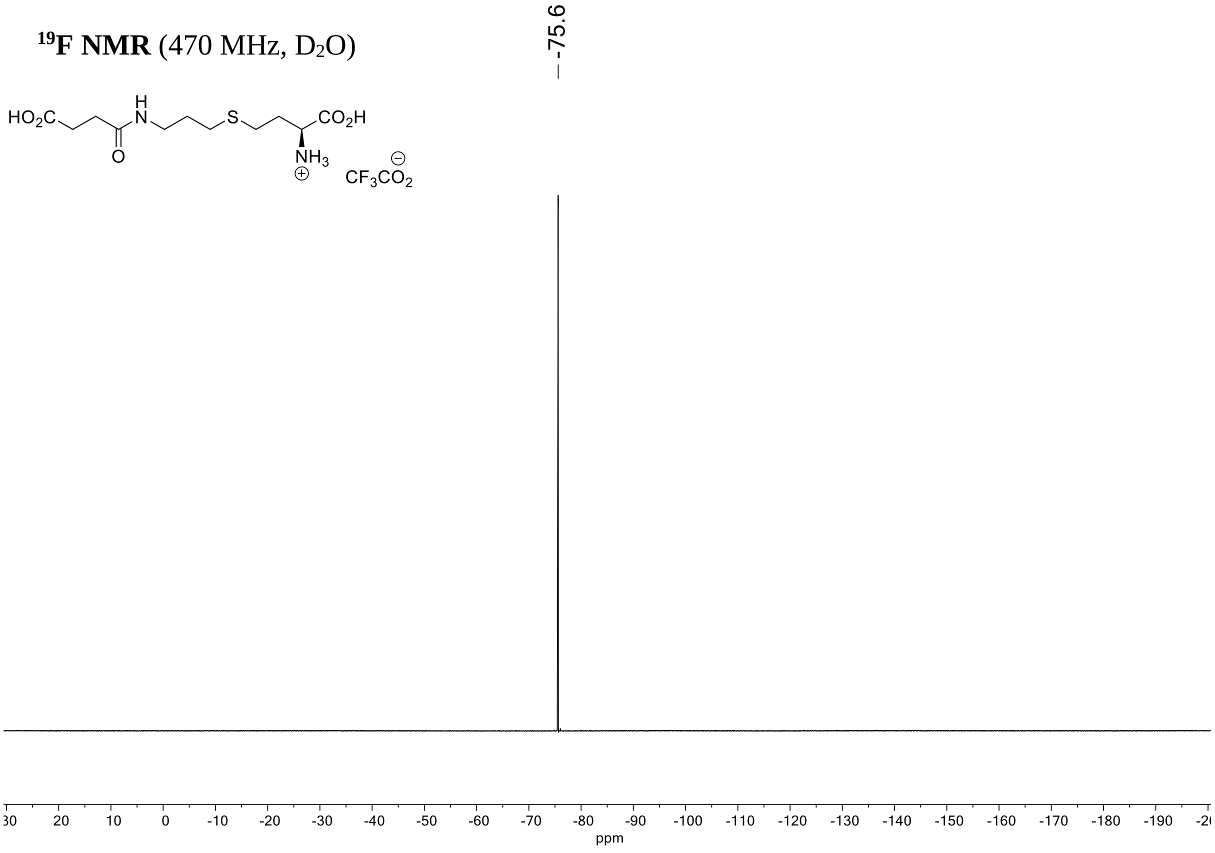

Trifluoroacetate salt of 4-((3-((2-aminoethyl)thio)propyl)amino)-4-oxobutanoic acid (14·TFA)

^{1}H NMR (500 MHz, D_2O)

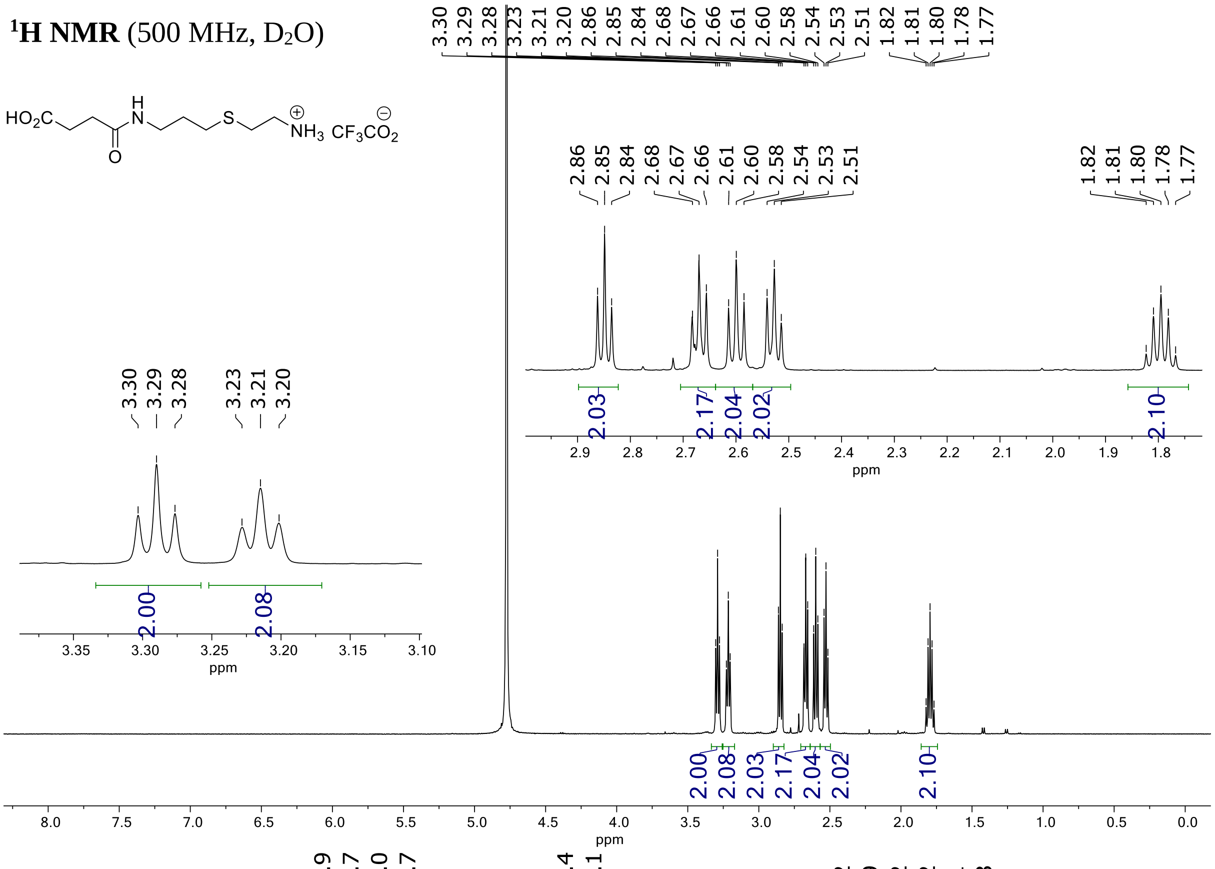

^{13}C{H} NMR (126 MHz, D_2O)

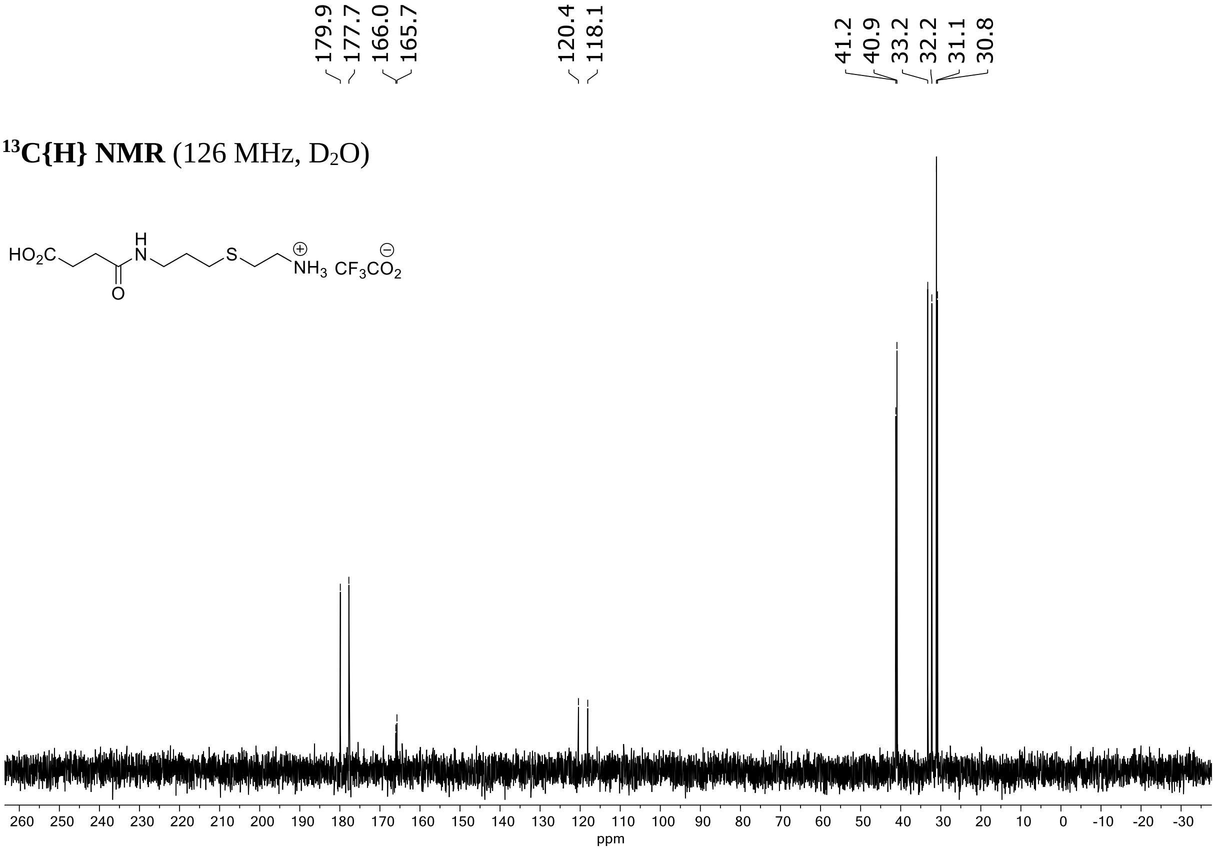

^{19}F NMR (470 MHz, D_2O)

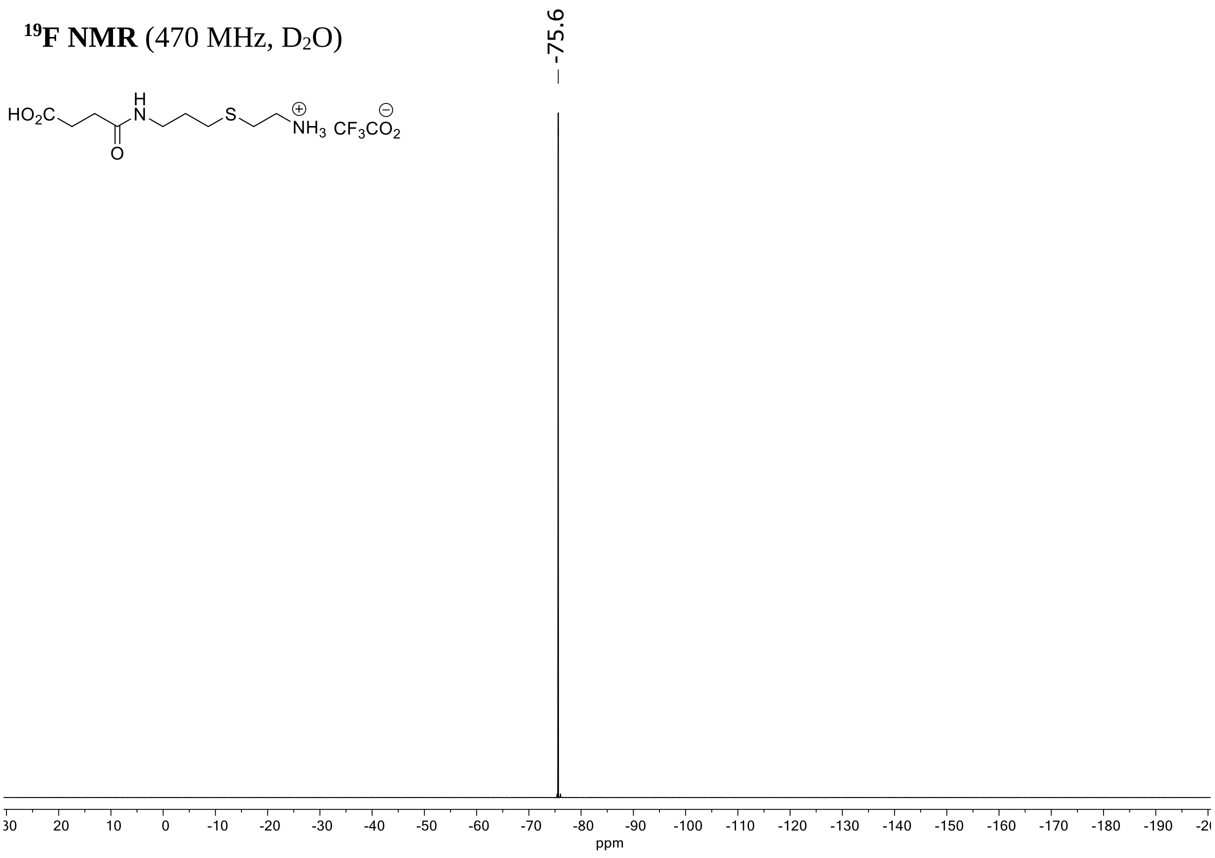

((*R*)-3-((3-hydroxy-3-methylbutyl)thio)-2-methylpropanoyl)-D-proline (15·TFA)

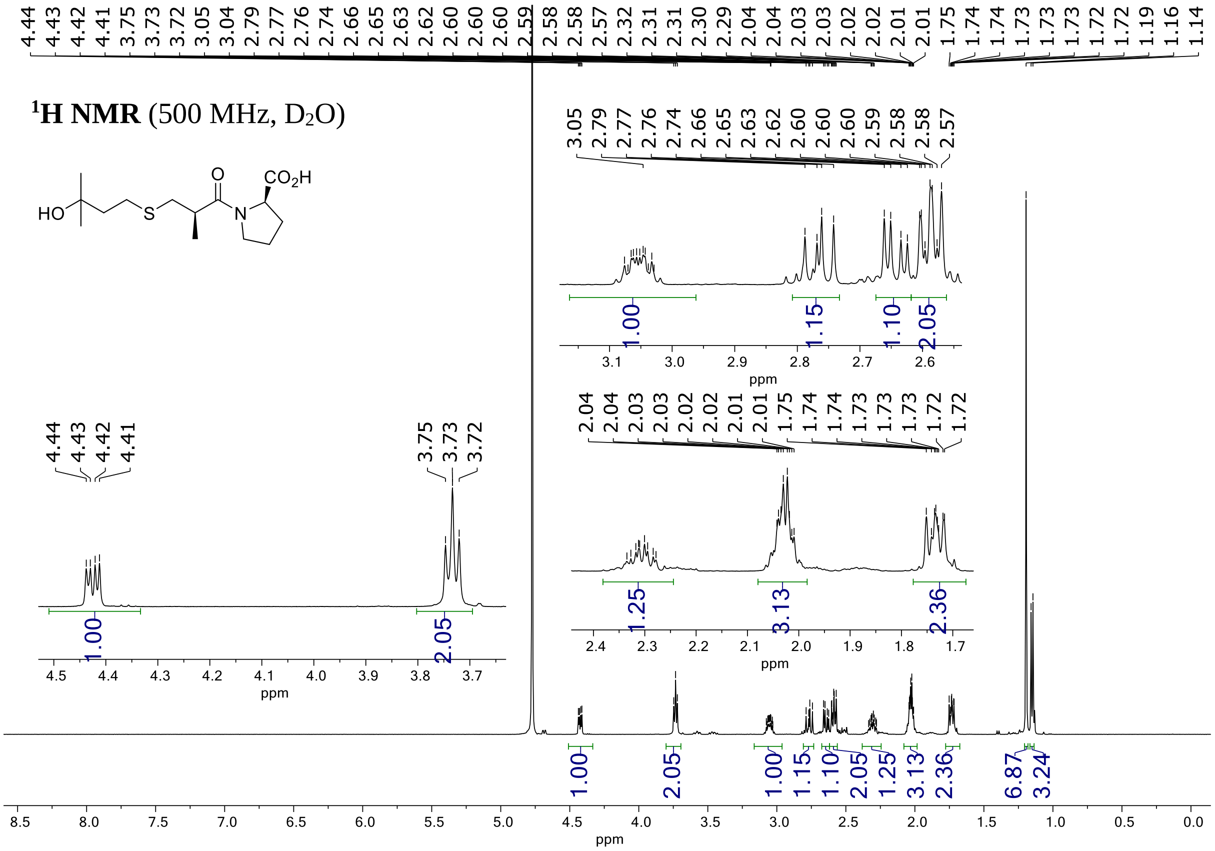

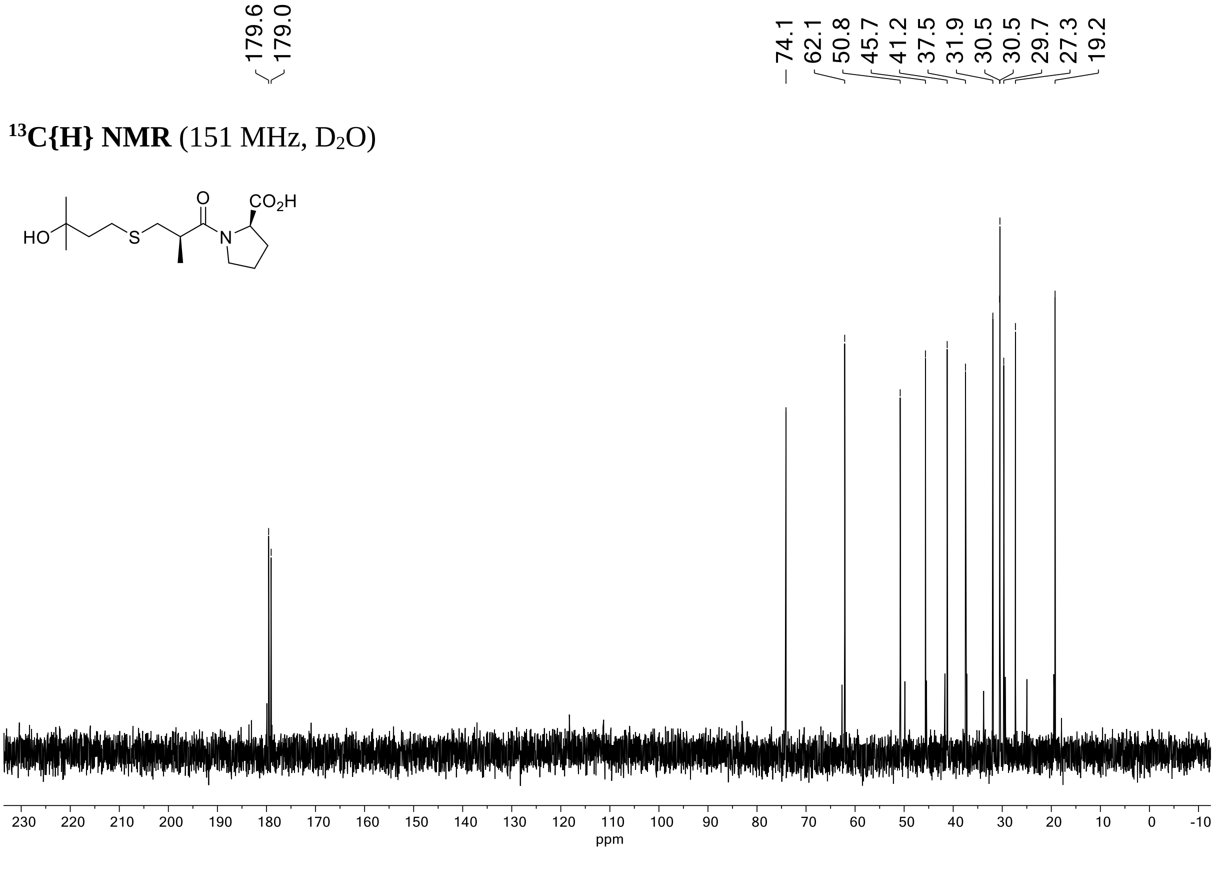

Trifluoroacetate salt of CoA conjugate (16·TFA)

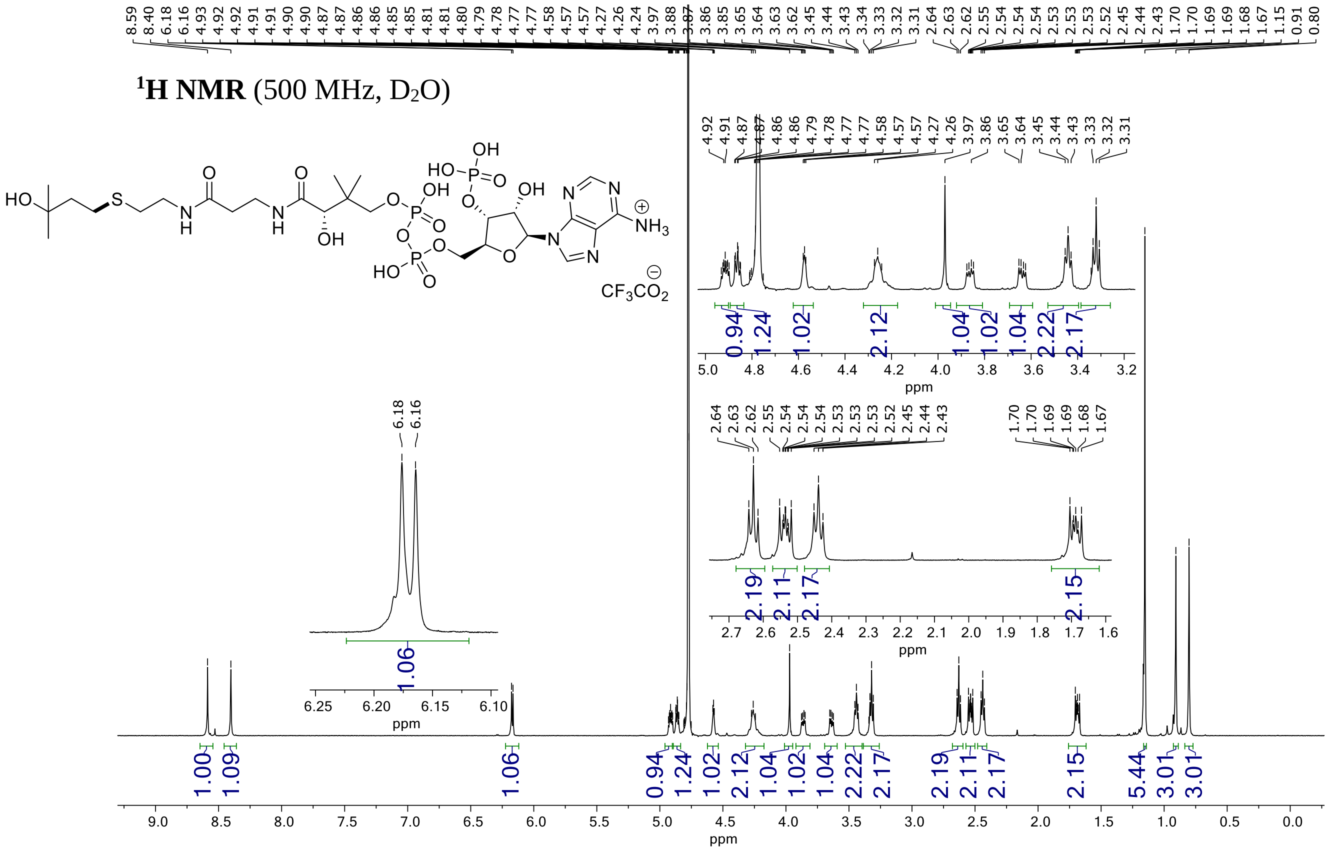

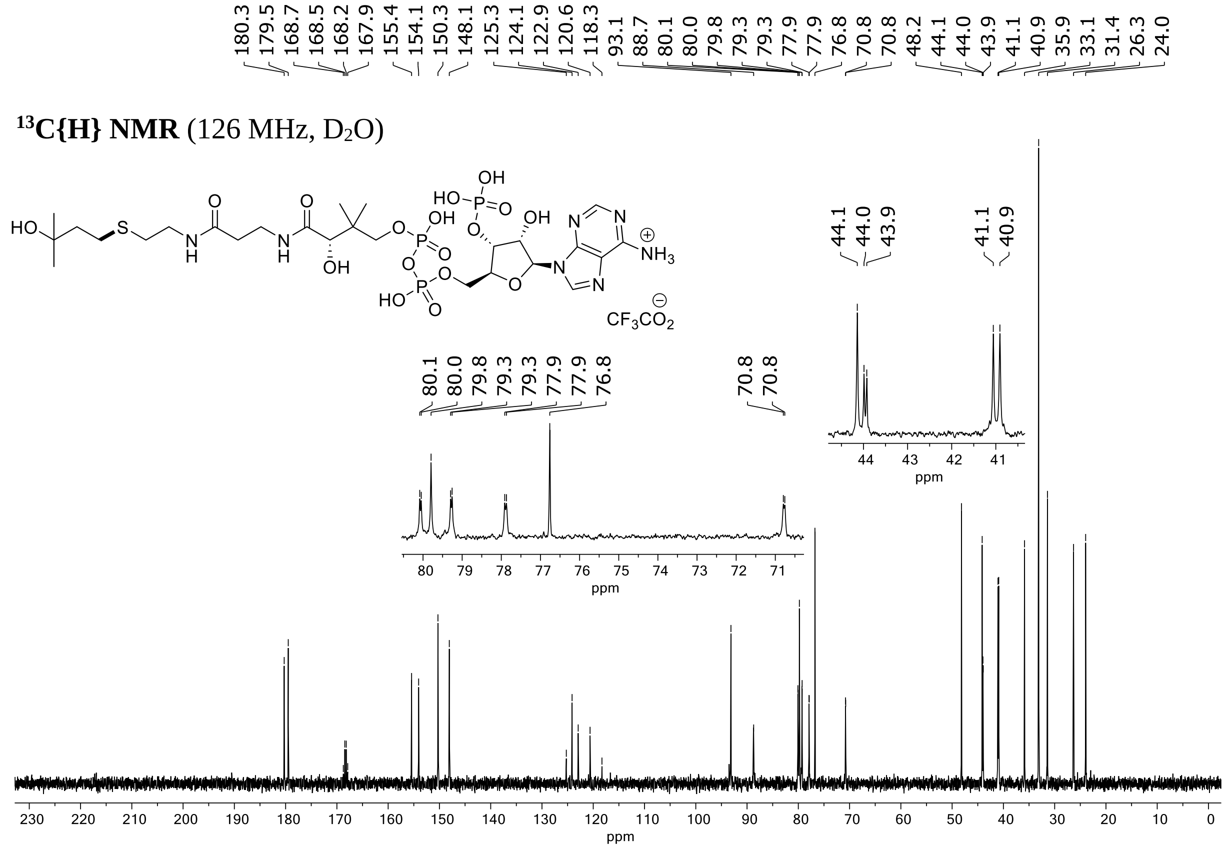

^{19}F NMR (470 MHz, D_2O)

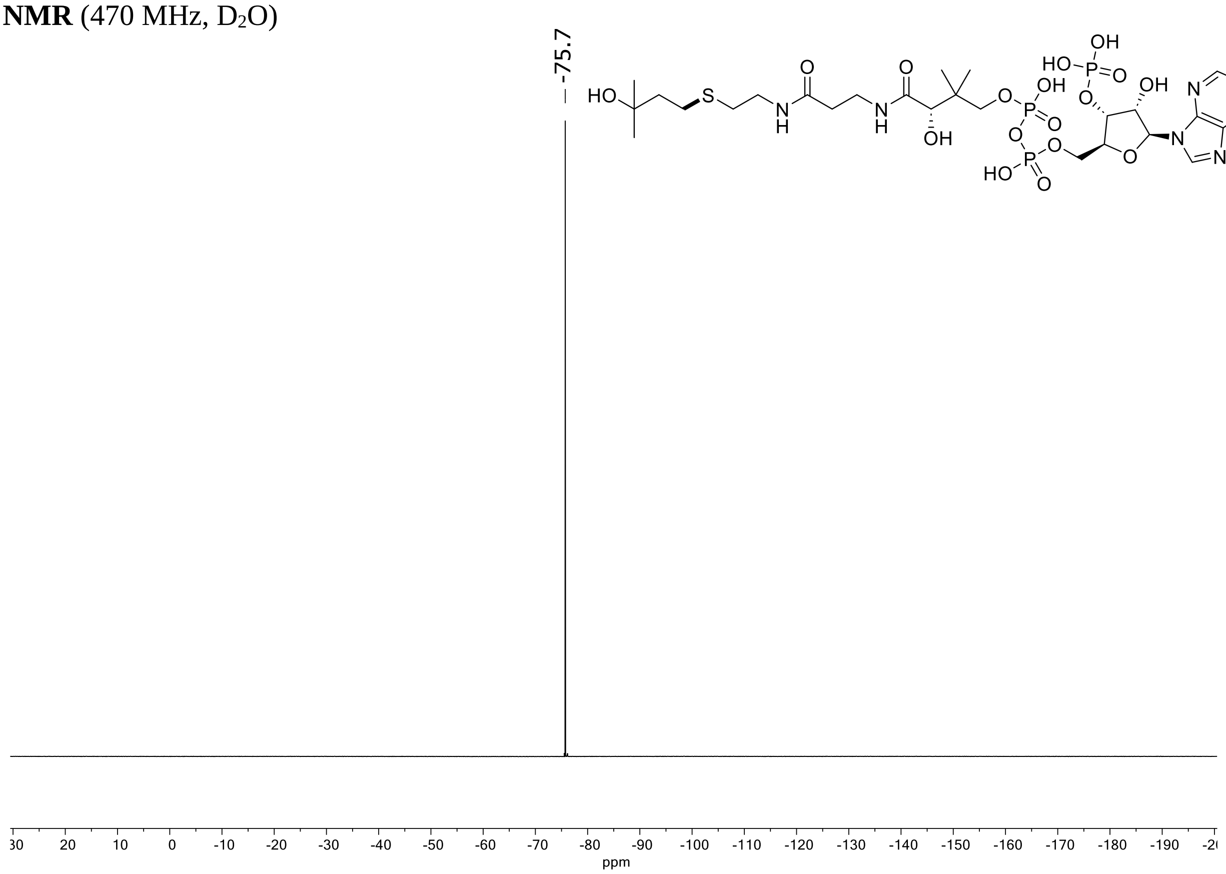

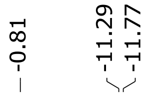

^{31}P NMR (202 MHz, D_2O)

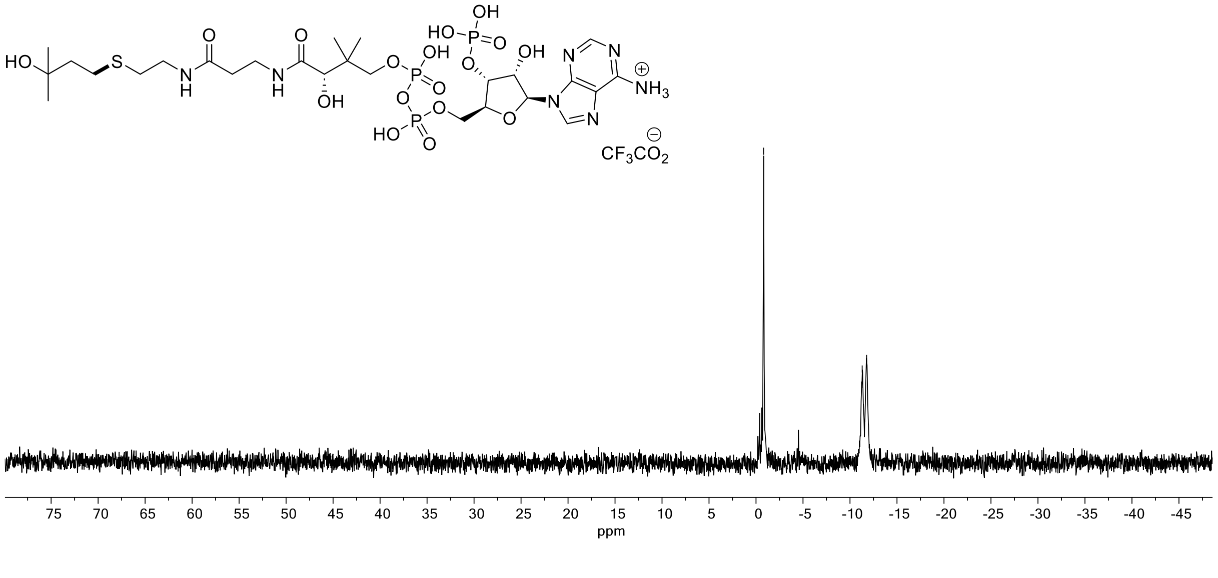

Supplement: Supplementary file 1 [file gg5c00025_si_001.pdf]
